# Molecular insights into the *VIRESCENS* amino acid sequence and its implication in anthocyanin production in red- and yellow-fruited cultivars of date palm

**DOI:** 10.1038/s41598-023-47604-9

**Published:** 2023-11-24

**Authors:** Nadia M. Alsuhaimi, Nadia S. Al-Kaff

**Affiliations:** Biology Department, College of Science, Taibah University, PO Box 30002, 14177 Al-Madinah Al-Munawarah, Kingdom of Saudi Arabia

**Keywords:** Plant genetics, Genetics, Plant sciences

## Abstract

This study examined the amino acid sequence of the *VIRESCENS* gene (*VIR*), which regulates the production of anthocyanin in 12 cultivars of the date palm (*Phoenix dactylifera* L.), grown in Al-Madinah Al-Munawarah of the Kingdom of Saudi Arabia. The gene products were amplified via polymerase chain reactions, amplifying both exons and introns. The products were sequenced for the reconstruction of a phylogenetic tree, which used the associated amino acid sequences. The ripening stages of Khalal, Rutab, and Tamar varied among the cultivars. Regarding *VIR* genotype, the red date had the wild-type gene (*VIR*^+^), while the yellow date carried a dominant mutation (*VIR*^*IM*^), i.e., long terminal repeat retrotransposons (LTR-RTs). The DNA sequence of *VIR*^*IM*^ revealed that the insertion length of the LTR-RTs ranged between 386 and 476 bp. The R2 and R3 motifs in both *VIR*^+^ and *VIR*^*IM*^ were conserved. The C-terminus motifs S6A, S6B, and S6C were found in the *VIR*^+^ protein sequence. However, the amino acids at positions 123, 161, 166, and 168 differed between *VIR*^+^ and *VIR*^*IM*^, and were not included in the C-terminus motifs. Within the *VIR*^+^ allele, the lysine at position 187 in the C-terminus was located immediately after S6B, with a protein binding score of 0.3, which was unique to the dark, red-fruited cultivars Ajwah, Anbarah, and Safawi. In the lighter, red-fruited cultivars, the presence of glutamic acid at the same position suggested that the anthocyanin regulation of date palm might be outside the R2 and R3 domains in the N-terminus.

## Introduction

The date palm *Phoenix dactylifera* L. (Family Arecaceae) is an archaic tree grown since the beginning of human civilisation^1^. The fruit consists of an exocarp, a fleshy mesocarp, a membranous endocarp, and a bone-like seed^2,3^. The fruit passes through five ripening stages within 6–7 months^2,4^, and the third one, known as Khalal, is the watershed for the exocarp colour variation among the cultivars of the date palm^5^. In general, the cultivars of the date palm are distinguished by their fruit colouration; the anthocyanin of the exocarp serves as an indicator of its ripeness and its categorisation as a fresh or dried fruit.

Anthocyanin is the primary pigment of various plant parts, such as flowers and fruits^6^. Within a plant, it is synthesised through the transcriptional regulation of the *R2R3-MYB* transcription factor (TF) family^7–12^. This TF family is part of the eminent myeloblastosis (*MYB*) gene family^13^; in its proteins, there are two highly conserved DNA binding motifs in the N-terminal, i.e., R2 and R3, and highly variable motifs in the C-terminal^14^. These motifs are imperfect repeats encoding three α-helices, while the R2 and R3 motifs fold as three helices, forming a binding structure for DNA^15^. In general, *R2R3-MYB* genes regulate several biological processes in plants, such as anthocyanin production and biotic and abiotic stress responses^16–19^. Genome analysis showed that 198 genes were of the *MYB* family and 126 genes were *R2R3-MYB*^20^. In particular, the Colored Aleurone 1 (C1) proteins produced from *R2R3-MYB* are required for seed colouring in maize^21^, while Purple Plant 1 (Pl) is responsible for the colouration of other plant tissues, such as leaves and flowers^22^.

Meanwhile, anthocyanin production in the date palm is regulated by the *R2R3-MYB* gene known as *VIRESCENS* (*VIR*)^8,11,23^, with a close association between the anthocyanin colour and the specific *MYB* TFs which regulate this process. In addition, these TFs control the biosynthesis, stability, and accumulation of anthocyanin^8,10,11,21,24–33^. The *VIR* gene regulates the exocarp colouration of the date, and it is expressed at the Khalal stage by the 105th day after fertilisation^11^. The date colour changes from apple green to dark red and light yellow, depending on the quantity of anthocyanin produced^18^. A lack of anthocyanins is related to the insertion of DNA in the *VIR* gene^9,18^. The wild-type cultivar is the red date, and the absence of anthocyanins gives rise to yellow-fruited cultivars^9,18^.

The genome size of the date palm is 772.3 Mb, comprising 28,595 genes^18^ across 18 chromosomes (2N = 36)^34^. The *VIR* gene is located on the 4th chromosome between loci 24,051,178 and 24,054,765 on the date palm genome I^18^. The first allele is the wild-type (*VIR*^+^) that regulates the genetic expression of a large amount of anthocyanin, leading to the colouring of the exocarp in various red grades^11^. Based on the genomic DNA and cDNA of the Khenezi cultivar (NCBI Gene ID: KT734805.1)^11^, the entire sequence of the *VIR*^+^ allele represents the red-fruited cultivars^11^. This *VIR*^+^ allele is 1653 bp in length and consists of three exons and two introns. The three exons encode the regions of the R2R3-MYB protein.

The wild-type VIR^+^ protein contains two helix-turn-helix (HTH) motifs (R2 and R3) with 234 amino acids^11^. In comparison, the yellow-fruited cultivar Lulu has a second *VIR* allele known as *VIR*^*IM*^ (previously called (*VIR*^*copia*^)^11^. This allele acts as a dominant negative mutation that inactivates the synthesis and accumulation of anthocyanin^11^. Like *VIR*^+^, the *VIR*^*IM*^ allele has three exons and two introns*.* However, the third exon is truncated by a premature stop codon at position 169, introduced by the insertion of the *IM-*like long terminal repeat retrotransposon (LTR-RT)^9^. The carboxy-terminal amino acids are then trimmed, causing a dominant negative mutation that inhibits the synthesis of anthocyanin synthesis while giving rise to the genotypes of *VIR*^+^/*VIR*^*IM*^ and *VIR*^*IM*^/*VIR*^*IM*^^18^. Recently, the *IM-*like (*VIR*^*IM*^) mutant allele was studied using a male date palm genome derived from a backcross with the Barhee cultivar^23^, and the complete sequence of the *IM* retrotransposon (LTR-RT) was genotyped. It was found to be 11.7 kb in size with 469 bp-long terminal repeats. A target duplication site of 5 bp was also identified^23^. Another allele was identified with the start codon polymorphism caused by a G to A change called *VIR*^*saf*^^23^.

Although the date palm is a valuable fruit crop in the Kingdom of Saudi Arabia (KSA), little is known about the genetic diversity of these cultivars. Therefore, this study aimed to examine the gene diversity of *VIR* in the regulation of the production of anthocyanin. Specifically, this study evaluated 12 date palm cultivars vary in their colour at Khalal stage. Genomic DNA was extracted to sequence the *VIR* gene from these cultivars. The gene sequences of *VIR* were then analysed with the published related sequences.

## Materials and methods

### Sampling the date palm and morphometric variation

Date palm fruits and juvenile leaflets were sampled from three orchards in Al-Madinah Al-Munawarah (Al’Awali, Sayyid Ash Shuhada, and Valley of Thalamah village) of the KSA; they encompassed 12 female cultivars. These cultivars were Ajwah, Anbarah, Baydh, Hilwah, Jebeli, Khalas, Labana, Rabiah, Rothanah, Safawi, Shalaby, and Sukkary. Additionally, one male date palm (Rabiah) was included in this study.

The date palms were harvested at the last three ripening stages, Khalal, Rutab, and Tamar, and 60 date palms were sampled randomly from 3 palm trees of each cultivar (i.e., 20 dates per palm tree). The date palms were then placed in labelled plastic bags and stored in the fridge for 24–48 h for further analysis. The ripening at these three stages, i.e., the changes in fruit colour, was also observed in the field. The exocarps of all of the cultivars changed from red and yellow at the Khalal stage, or to black or brown at Rutab and Tamar stages, depending on the cultivars. However, the exocarps of the Labana dates changed partially, from yellow at the Khalal stage to brown at the Tamar stage. Therefore, the mid-height width (MHW) and mesocarp width (MW) of each date palm were measured for Labana and compared to those of Khalas using the software application Tomato Analyser (TA; version 3, developer)^35,36^ and the Electronic Digital Caliper (EDC), respectively, at the Khalal, Rutab, and Tamar stages. These measurements were indicative of the changes from one ripening stage to another due to moisture reduction^37^. The dates were first halved, then scanned with a scanner imager (HP Deskjet 1510) and saved in the JPEG file format for the TA and EDC measurements.

Additionally, juvenile leaflets were collected from the crown heart of the date palm for DNA extraction. The new leaflets were cut into small pieces and dried in an oven at 30 ± 5 °C for seven days. The dried leaflets were ground to powder using an electric grinder (SF Stardust Model: CM-1400 MKII), and the powdered samples were stored in labelled aluminium foil envelopes at room temperature. For each cultivar, all of the leaflet and fruit samples were collected from the same palm tree.

### DNA sequences from databases

Sequences of the *VIR* gene were identified from the Khalas genome (Gene National Center for Biotechnology Information (NCBI) ID: Loc103717680)^38^, and they were used to query the NCBI database (www.ncbi.nlm.nih.gov) via the blast search tool. Altogether, three sequences with a high identity (> 90%) were downloaded from the NCBI database. These sequences included two *VIR* homologs and one orthologous gene. The red cultivar Khenezi (KT734805.1) and the yellow cultivar Lulu (KT734804.1)^11^ comprised the homologs; the orthologous gene belonged to the oil palm (*Elaeis guineensis*) genome (KJ789862.1)^8^, which was syntenic to that of the date palm. Additionally, a fourth sequence from the Barhee cultivar of the date palm (BC4 Male Pdac_HC_chr4T0137100)^23^ was downloaded using the JBrowse browser tool (www.datepalmgenomehub.abudhabi.nyu.edu/).

### DNA extraction and purification

For the ten cultivars Ajwah, Anbarah, Baydh, Hilwah, Jebeli, Khalas, Labana, Rothanah, Safawi, and Sukkary, DNA was extracted from 100 mg of the dried ground sample of each juvenile leaflet using a modified cetyl-trimethylammonium bromide (CTAB) method^39^. For the remaining two cultivars, i.e., Shalaby and Rabiah, including the male date palm Rabiah, DNA was extracted using the GeneJET Plant Genomic DNA Purification Mini Kit (Thermo Fisher Scientific) with the addition of polyvinylpyrrolidone. DNA purity was quantified using a spectrophotometer (NanoDrop™ 2000c, Thermo Fisher Scientific). DNA quality was electrophoresed via a 0.8% agarose gel and visualised alongside a 1 kb Plus DNA ladder, 100–10 kb (Cleaver Scientific Ltd) using the omniDOC™ Gel Documentation System (Cleaver Scientific Ltd).

### Primer design and polymerase chain reaction (PCR)

The primers were designed by aligning the nucleotide sequences of the three known *VIR* homologous genes from the Khenezi, Khalas, and Lulu cultivars^11,40^ using the software Clustal Omega (www.ebi.ac.uk)^41,42^. The alignment showed high similarity among these sequences. The sequences of the designed primers were sent to Macrogen Inc. (Seoul, South Korea) for synthesis (Table 1 and S1).

**Table 1:** Primer sets that were used for the amplification of VIR^+^ and VIR^IM^ genes, product size of PCR, and the actual annealing temperature (Ta) used.

Primer sets	Expected PCR size products (bp)	Coverage	Ta °C
DPVIRF1-DPVIRR1	671	Exons 1 and 2 and part of Intron 2	47
DPVIRF2-DPVIRR2	579	Intron 2	47
DPVIRF2-DPVIRR3R	1014	Intron 2 and exon 3	50
DPVIRF2-DPVIRR3Y	1195	Intron 2 and exon 3	50
DPVIRF3-DPVIRR3R	454	Exon 3	50
DPVIRF3-DPVIRR3Y	640	Exon 3	50

Each PCR mixture was prepared in a 15 µl final volume reaction with a hot start master mix of 7.5µl (Thermo Fisher Scientific™ DreamTaq™ Hot Start Green PCR Master Mix (2×) Kit). Primers (forward and reverse) were used at 0.2 µM each, with a DNA template of 25–50 ng; the reaction volume was completed with nuclease-free water. The PCR amplification was performed using a thermal cycler (Applied Biosystems *Veriti*™ Thermal Cycler) with a specific annealing temperature for each primer set (Table 1) for 25–30 cycles. The PCR products were electrophoresed in a 0.8% agarose gel at 80–95 V for 45 min to confirm their sizes, using a DNA marker of 10 kb (Cleaver Scientific). PCR was also used to establish the genotype of the VIR gene for each cultivar.

### PCR product sequencing

The PCR products were sent to Macrogen Inc. (Seoul, South Korea) for sequencing, using forward and reverse primers with three replicates each (three reactions with forward and three with reverse primers). The 87 raw chromatographic DNA files were edited using the software BioEdit (v7.0.5.3)^43^. The DNA sequences of both the *VIR*^+^ and the *VIR*^*IM*^ alleles were translated into amino acid sequences using the translation tool Expasy (web.expasy.org). Both the DNA and the amino acid sequences were aligned using Clustal Omega for multiple sequence alignment (www.ebi.ac.uk) and homolog reference sequences^11,23,40^. Different nucleotide and amino acid sequences of the *VIR* orthologs were obtained from NCBI (www.ncbi.nlm.nih.gov). Alignments of the orthologs with the date palms were carried out using the MUSCLE software^44^.

A phylogenetic tree of orthologs was reconstructed based on the complete amino acid sequences using the maximum likelihood (ML) method and the Poisson correction model^45^, implemented in the software of MEGA, version 11^46^. The phylogenetic tree was bootstrapped with 1000 replicates for statistical reliability^47^. Meanwhile, the web-based application WebLogo (https://weblogo.berkeley.edu/)^48,49^ was used to compare the amino acid sequences for R2 and R3, the DNA binding domains (DBDs). Altogether, the amino acid sequences of the DBDs were compared for 32 species to reconstruct the phylogenetic tree of the *VIR* orthologs, monocots, and dicots.

The motifs of the C-terminus were found and characterised based on the findings of other studies^50,51^. The online software programs IUPred2A and DISOPRED3^52,53^ were used to identify the intrinsically disordered regions (IDRs) in order to predict the motifs in the C-terminus in the *VIR*^+^ allele of the date palm. Clustal Omega^41^ was used to align the *VIR*^+^ alleles of Ajwah and Anbarah with the identified amino acid mutations and to compare them with Jebeli, *E. guineensis*, and three other sequences of R2R3-MYB from the R2R3 subgroup 6 (S6) of another study^51^, i.e., MdMYB10, VvMYB1r, and AcMYB110^50^.

### Statistical analysis

The data were then statistically analysed with the one-way analysis of variance (ANOVA) test and Tukey’s pairwise comparison test, using the software Minitab (version 19, Minitab, LLC) (www.minitab.com) at the significance level (α) of 0.05.

### Ethics approval and consent to participate

Dates and leaflets from different cultivars were collected from date palm orchards; this was permitted by the date palm orchard owners. The plant collection and the study complied with local and national (Kingdom of Saudi Arabia) regulations. The MSc proposal for this study was approved by the Biology Department Council, College of Science, Taibah University, Kingdom of Saudi Arabia. All the methods in this manuscript were carried out in accordance with relevant guidelines and regulations.

## Results

### The colouration development of the date palm at various ripening stages

**Figure 1:**
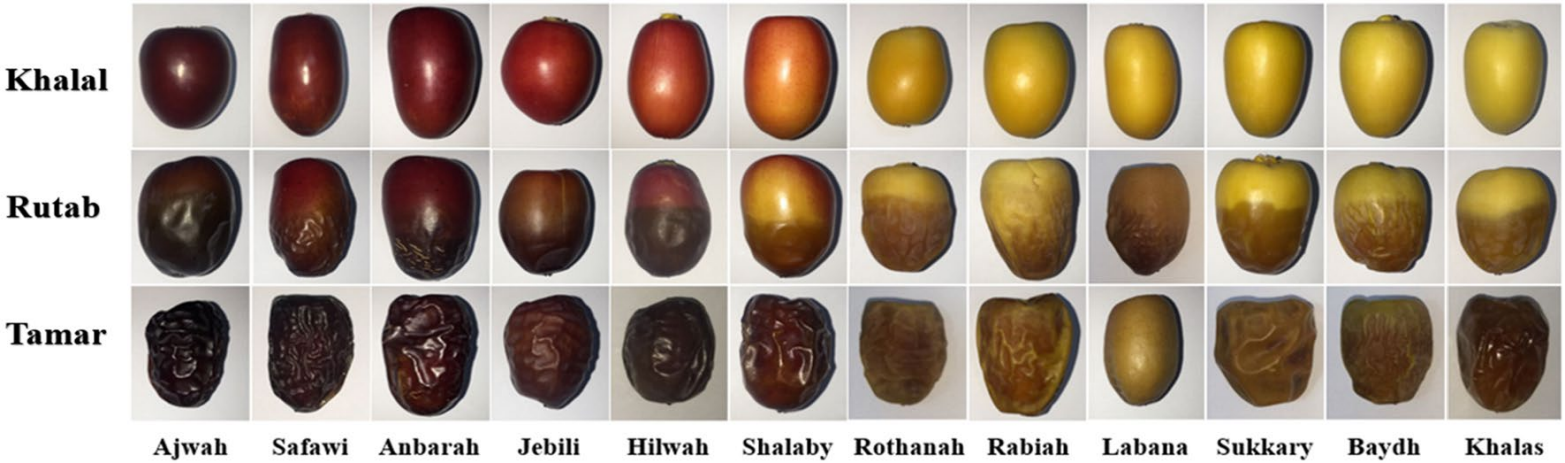
The ripening stages of Khalal, Rutab, and Tamar for the dates sampled from 12 cultivars: Ajwah, Anbarah, Baydh, Hilwah, Jebeli, Khalas, Labana, Rabiah, Rothanah, Safawi, Shalaby, and Sukkary.

Figure 1 shows the colour variation in the date palms of the 12 cultivars at the ripening stages of Khalal, Rutab, and Tamar. In general, the entire date turned red or yellow at Khalal. In comparison, at Rutab, the tip started turning black or brown with a slight reduction in textural firmness, depending on the cultivar. At Tamar, the entire date turned black or brown (Fig. 1) with a soft texture. The cultivars of Baydh, Jebeli, Khalas, Labana, Rabiah, and Sukkary were edible at the ripening stages of Rutab and Tamar, and they were yellow, except for Jebeli (red). The other cultivars, such as Ajwah, Anbarah, Safawi, and Shalaby were edible at Tamar, and they were red; Hilwah (red) and Rothanah (yellow) were consumable at Khalal and Rutab, respectively (Table 2).

**Table 2:** The number of days between different ripening stages in the sampled dates.

Date colour	Cultivar	Interval between different ripening date stages when collected (days)		
		Khalal–Rutab	Rutab–Tamar	Khalal–Tamar
Red dates	Ajwah^T^	47	0*	47
	Anbarah^T^	15	7	22
	Safawi^T^	35	*	35
	Jebeli^RT^	24	12	36
	Hilwah^K^	16	0*	16
	Shalaby^T^	0*	8	08
Yellow dates	Rothanah^R^	12	0*	12
	Rabiah^RT^	12	06	18
	Labana^RT^	18	23	41
	Sukkary^RT^	0*	08	08
	Baydh^RT^	02	0*	02
	Khalas^T^	06	0*	06

*Fruits from both stages were collected on the same day. Superscripts K, R, and T denote the edible stage for a particular cultivar at the different ripening stages of Khalal^(K)^, Rutab^(R)^, and Tamar^(T)^.

The cultivars also varied distinctively in terms of the duration (days) between ripening stages (Table 2 and Fig. 1). Some cultivars, such as Shalaby and Sukkary, bore fruits simultaneously at two ripening stages, i.e., Khalal and Rutab, on the palm bunch. Other cultivars, i.e., Ajwah, Baydh, Hilwah, Khalas, Rothanah, and Safawi, also carried dates at two stages simultaneously, but during Rutab and Tamar (Table 2; Fig. 1). On average, the red-fruited cultivars took a longer time to ripen from the Khalal to Tamar stages (27.3 ± 14.5 days; range: 22–47 days), but the red Shalaby took just 8 days to mellow. In general, the yellow-fruited cultivars matured faster from Khalal to Tamar (an average of 14.5 ± 14.1 days), but were more variable (2 to 18 days). However, the yellow-fruited Labana took 41 days to mature from Khalal to Tamar (Table 2).

**Figure 2:**
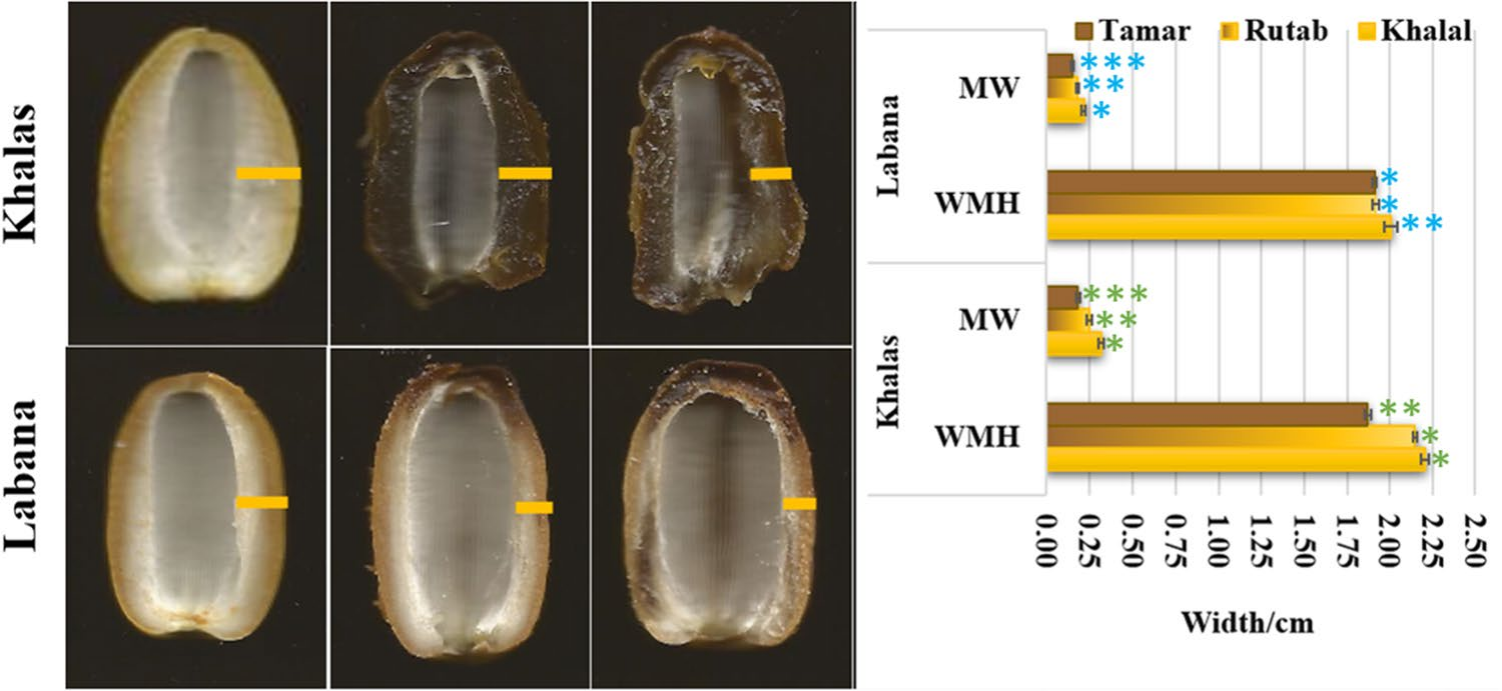
Morphometric variation of mid-height width (MWH) and mesocarp width (MW) in the Khalas and Labana dates at Khalal, Rutab, and Tamar ripening stages. The yellow line is the MW measurement; the number of asterisks (*) represents statistical significance at α = 0.05**.**

Changes in date colouration from the Rutab to Tamar stages began at the fruit tip and moved to the base with a gradual spread of the darker colour. The exocarp gradually changed from red to black or from yellow to brown. The colouration spread inwardly from the darker parts, i.e., the exocarp and mesocarp, to the entire date at the Tamar stage, except for the cultivar Labana (Fig. 2). Some of the Labana dates entered into the Rutab stage with a partial brown colour, but the others showed no changes. Thus, to distinguish the ripening stages of the Labana dates, their MHW and MW values were compared to those of the Khalas dates.

For the Khalas dates, their MHW measurements showed no significant difference (*p* > 0.81) (Table S2) between the Khalal (2.20 ± 0.02 mm) and Rutab (2.14 ± 0.13 mm) ripening stages, but the dates at these two stages differed significantly (*p* = 0.00) (Table S2) from those at the Tamar stage (1.86 ± 0.10 mm). However, the MHW measurements of the Labana dates differed significantly (*p* = 0.00) between Khalal (2.00 ± 0.10 mm), Rutab (1.92 ± 0.06 mm), and Tamar (1.91 ± 0.10 mm). No significant differences were found between the last two stages of the Labana dates (Fig. 2 and Table 2). In general, the mesocarps of the Khalas dates turned dark brown and soft at the Rutab and Tamar stages (Fig. 2). Expectedly, the mesocarps of the Labana dates partially changed to brown even at the last stage of ripening, and their exocarps partly stayed yellow. Meanwhile, the mesocarp texture of the Labana dates was dry compared to that of the Khalas dates, particularly if the exocarps of the Labana dates did not change colour and remained yellow. Overall, the MW measurements decreased at Rutab and Tamar for both cultivars but more so for the Khalas than for the Labana dates (Fig. 2; Table 2).

### Molecular analysis of the VIR gene in date palm cultivars

**Figure 3:**
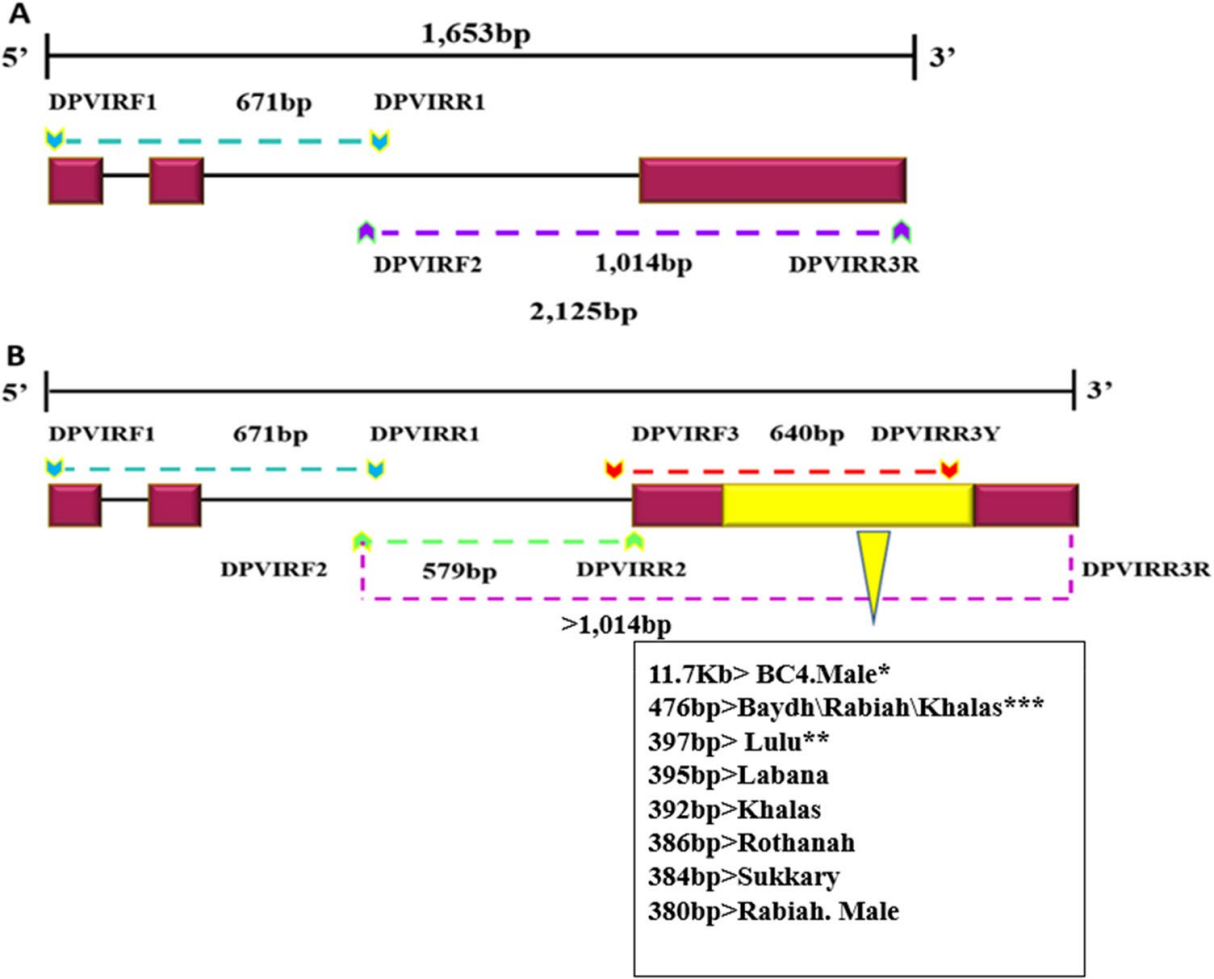
The structure of the VIR gene of the date palm cultivars: (**A**) the red VIR^+^ allele, and (**B**) the yellow VIR^IM^ alleles. Solid black lines at the top of each figure denote the full size of the gene. Maroon boxes indicate the locations of exons, and the yellow box represents the insertion of the IM LTR retrotransposon at the C-terminus region of exon 3. Black lines between the maroon boxes represent introns, while the coloured hash lines denote fragments produced by PCR and sequenced, each starting and ending with primer names shown by arrows. Also shown is the size of the LTR-RT in each of the sequenced yellow cultivars. Cultivars with *, **, and *** are extracted from References^11,23,40^. See also Table 3 for the size of the LTR-RT.

The *VIR*^+^ allele in the red cultivars (Ajwah, Anbarah, Hilwah, Jebeli, Safawi, and Shalaby) was sequenced using different primer sets. Specifically, the primer set of DPVIRF1-DPVIRR1 amplified exons 1 and 2 and part of intron 2, yielding a gene fragment of 671 bp, while primers DPVIRF2-DPVIRR3R covered intron 2 and exon 3 with a PCR product of 1014 bp (Table 1; Fig. 3).

By contrast, the *VIR*^*IM*^ allele of the yellow cultivars (Baydh, Khalas, Labana, Rabiah, Rothanah, and Sukkary), inclusive of the male date palm, was sequenced using five primer sets. The first product, generated from the amplification of the DPVIRF1-DPVIRR1 primers, was 671 bp, encompassing exons 1 and 2 and part of intron 2. The primer set of DPVIRF2-DPVIRR3Y produced the second PCR fragment with 1195 bp for the sequencing of the cultivars of Baydh, Rabiah, Rothanah, and Sukkary (Table 1; Fig. 3A and B). The third and fourth primer sets, DPVIRF2-DPVIRR2 and DPVIRF3-DPVIRR3Y, produced PCR fragments of 579 bp and 640 bp, respectively, for the sequencing of the cultivars of Labana, Khalas, and the male Rabiah (Table 1; Fig. 3B). The last primer set, DPVIRF2-DPVIRR3R, produced a 1014 bp PCR fragment for the sequencing of the cultivars of Baydh, Labana, female Rabiah, and Rothanah, for the identification of the end of the *VIR*^*IM*^ gene, after the locus of the insertion of the *IM* retrotransposon (Table 1 and Fig. 3B). All of the amplified fragments conformed to the expected sizes.

**Figure 4:**
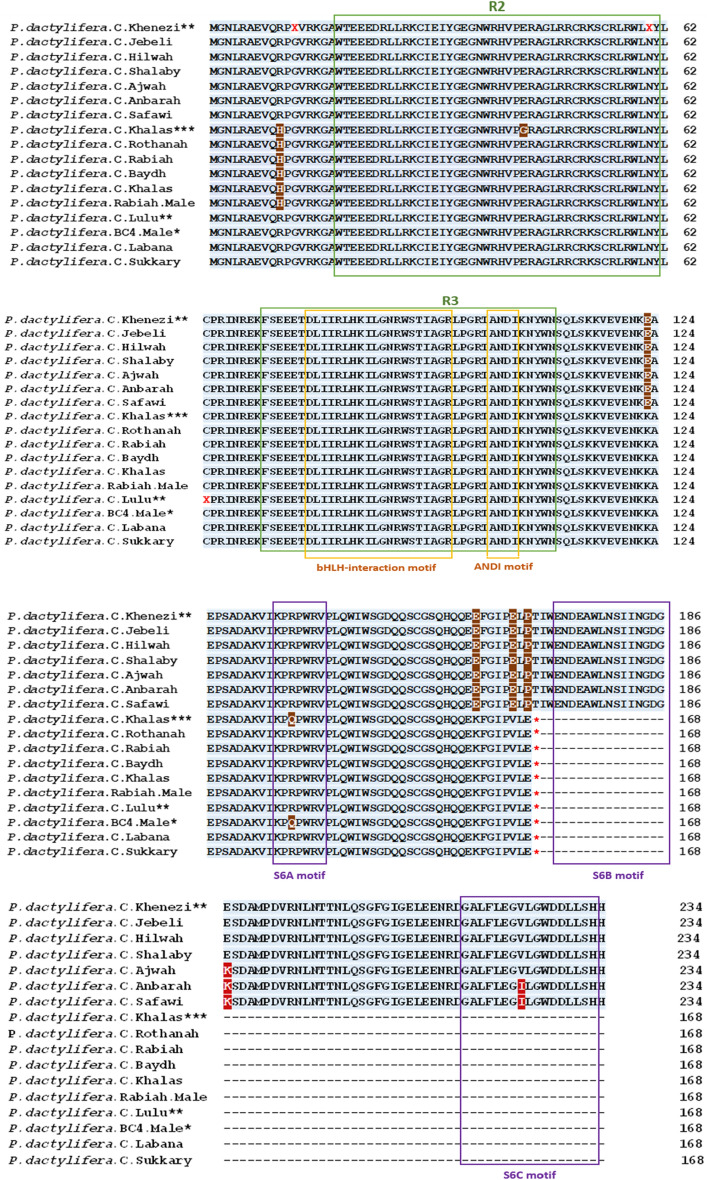
Comparison of amino acid alignment for the red- and yellow-fruited cultivars of this study with published *VIR* alleles of cultivars Khenezi^11^, as a reference for *VIR*^+^, and Khalas^40^, Lulu^11^, and BC4 Male*^23^ as references for the *VIR*^*IM*^ allele. Amino acids in light green boxes are the R2 and R3 DNA binding site domains^11^. Amino acids in dark brown varied between *VIR*^+^ and *VIR*^*IM*^. Amino acids in dark red varied within *VIR*^+^. The motifs in the R3 domains are based on those of another study. Motifs in the C-terminus motifs (S6A, S6B, and S6C highlighted in boxes with a purple border) are based on those of other studies^50,51^. The asterisk (*) indicates the occurrence of stop codons at position 169 in the* VIR*^*IM*^ sequence^11^*.*

The sequence alignments of the various *VIR*^+^ alleles showed that most nucleotide differences occurred in introns 1 and 2 (Figure S1). However, the exons were also found to contain nucleotide variations, some of which were missense substitutions, while others changed the amino acids (Figure 4). Additionally, there were two nucleotide changes in exon 3 at positions 1510 and 1621 that caused a substitution in amino acids (as discussed later in the section entitled “Variation of the amino acid sequences in the VIR protein”).

The sequences of the *VIR*^*IM*^ allele (the yellow-fruited cultivars) were aligned in two parts. The first part consisted of exons 1, 2, and 3, including the insertion of the *IM* retrotransposon, and introns 1 and 2 (Figure S2). The second part comprised the *IM* retrotransposon sequence only (Figure S3). The alignments showed high similarity in the exons and introns of *VIR*^*IM*^ (Figure S2). However, a single substitution (at the tenth amino acid) in the red cultivars was similar to that of two other yellow cultivars, i.e., Sukkary and Labana (Table 4; Figure 4). Also, there was a deletion of four nucleotides in the second intron of the yellow-fruited cultivars, except for Labana (Figure S2). For Labana, this specific deletion was similar to that of the red cultivars (Figure S1). Also, a deletion of 15 bp at the end of exon 3 occurred in the Baydh cultivar but not in the other yellow-fruited cultivars (Figure S2). 

In general, the sequence length of *IM* varied among the yellow-fruited cultivars. Rabiah and Baydh showed the longest complete sequence of *VIR*^*IM*^, i.e., 476 bp, followed by Labana, Khalas, Rothanah, Sukkary, and the male Rabiah with 395, 392, 386, 384, and 380 bp, respectively. All of these yellow-fruited cultivars showed high similarity in the *IM* retrotransposon nucleotide sequences with deletions of 90bp and 81bp in Rothanah and Labana, respectively (Table 3; Fig. 3).

**Table 3:** Variation of the *VIR*^*IM*^ sequence length in various yellow-fruited cultivars.

Cultivar	*IM* LTR-RT insertion (bp)
Female Rabiah	476
Baydh	476
Labana	395
Khalas*	392
Rothanah	386
Sukkary*	384
Male Rabiah*	380

Cultivars with an asterisk (*) were partially sequenced.

Table S3 shows that all of the red-fruited cultivars were homozygotes (*VIR*^+^/*VIR*^+^). In comparison, the yellow-fruited cultivars comprised both homozygotes *VIR*^*IM*^/*VIR*^*IM*^ (Baydh, Sukkary, Khalas, and the male Rabiah) and heterozygotes *VIR*^+^/*VIR*^*IM*^ (Rothanah, the female Rabiah, and Labana).

### Variation of the amino acid sequences in the VIR protein

Figure 4 compares the amino acids between the *VIR*^+^ and *VIR*^*IM*^ alleles and those marked with R2 and R3 domains^11^. The motifs were named following the method of another study^54^. Each allele had four unique amino acids at positions 123, 161, 166, and 168. Interestingly, two of these four amino acids changed from glutamic acid (E) to lysine (K). The third changed from proline (P) to E, and in the last one, E was converted to valine (V). In the N-terminus, the arginine (R) at position 10 changed to histidine (H) in *VIR*^+^, two *VIR*^*IM*^ cultivars (Labana and Sukkary), and two references, i.e., BC4 Male and Lulu^11,23^ (Table 4; Figure 4). Within the *VIR*^+^ allele, few amino acids differed. The darker-coloured Ajwah, Anbarah, and Safawi had K at position 187 in the N-terminus, while the rest of the *VIR*^+^ cultivars had E at that position. The cultivars Anbarah and Safawi had another unique change with isoleucine (I) at position 224, while the rest of the *VIR*^+^ cultivars had V.

**Table 4:** Summary of the polymorphic amino acids between the red- and yellow-fruited date palm cultivars.

Date colour	Cultivar	Amino acids								
		10	43	123	136	161	166	168	187	224
Red dates	*P. dactylifera*.C.Ajwah	R	E	E	R	E	E	P	**K**	V
	*P. dactylifera*.C.Anbarah	R	E	E	R	E	E	P	**K**	**I**
	*P. dactylifera*.C.Safawi	R	E	E	R	E	E	P	**K**	**I**
	*P. dactylifera*.C.Khenezi^11^	R	E	E	R	E	E	P	E	V
	*P. dactylifera*.C.Jebeli	R	E	E	R	E	E	P	E	V
	*P. dactylifera*.C.Hilwah	R	E	E	R	E	E	P	E	V
	P. dactylifera.C.Shalaby	R	E	E	R	E	E	P	E	V
Yellow dates	*P. dactylifera*.BC4 Male^22^	**R**	E	K	**Q**	K	V	E		
	*P. dactylifera*.C.Khalas^40^	H	**G**	K	**Q**	K	V	E		
	*P. dactylifera*.C.Rothanah	H	E	K	R	K	V	E		
	*P. dactylifera*.C.Rabiah	H	E	K	R	K	V	E		
	*P. dactylifera*.C.Baydh	H	E	K	R	K	V	E		
	*P. dactylifera*.C.Khalas	H	E	K	R	K	V	E		
	*P. dactylifera*.Rabiah.Male	H	E	K	R	K	V	E		
	*P. dactylifera*.C.Lulu^11^	**R**	E	K	R	K	V	E		
	*P. dactylifera*.C.Labana	**R**	E	K	R	K	V	E		
	*P. dactylifera*.C.Sukkary	**R**	E	K	R	K	V	E		

Note: polymorphic amino acids are bold with each colour date phenotype.

All of the amino acid changes happened to be outside the R2 and R3 domains and their motifs. The R2 domain was located between exons 1 and 2, while R3 was between exons 2 and 3 (Fig. 4). One amino acid, i.e., glycine (G), was reported^40^ to occur uniquely in Khalas at position 43 within the R2 domain. However, as with the other yellow-fruited cultivars, the Khalas Al-Madinah sequenced in this study had the same amino acid (E) at this position (Table 4; Fig. 4). Another amino acid, the glutamine (Q) at position 136, also differed in Khalas and the BC4 Male references, according to the findings of other studies^23,40^. However, none of the cultivars sequenced in this study had this change.

Based on the findings of other studies^50–53^, selected date palm VIR^+^ cultivars Jebeli, Ajwah and Anbarah, and the related genes from the anthocyanin R2-R3-MYB subgroup S6 AcMYB110, VvMYBBA1r, and MdMYB10 published in another study^51^, were aligned (Figure S4). The first motif identified was S6A, which was located from amino acids 133 to 140. In this study, all of the amino acids within this region were conserved in *VIR*^+^ and *VIR*^*IM*^ for the 12 cultivars (Fig. 4). The second motif, S6B, was assigned between 172 and 186, and it was conserved in the *VIR*^+^ allele of this study with a content of 60% hydrophobic and acidic amino acids. The third motif, S6C, was located from 217 to 233 with 70.5% hydrophobic and acidic amino acids (Figs. 4 and S4). In S6A, the amino acid P at positions 134 and 136 was conserved among the various species compared. The amino acid tryptophan (W) at 177 was conserved in S6B, and semi-conserved in S6C at position 227 (Figs. 4 and S4).

### Protein alignment of date palm *VIR* gene with R2R3-MYB orthologs

**Figure 5:**
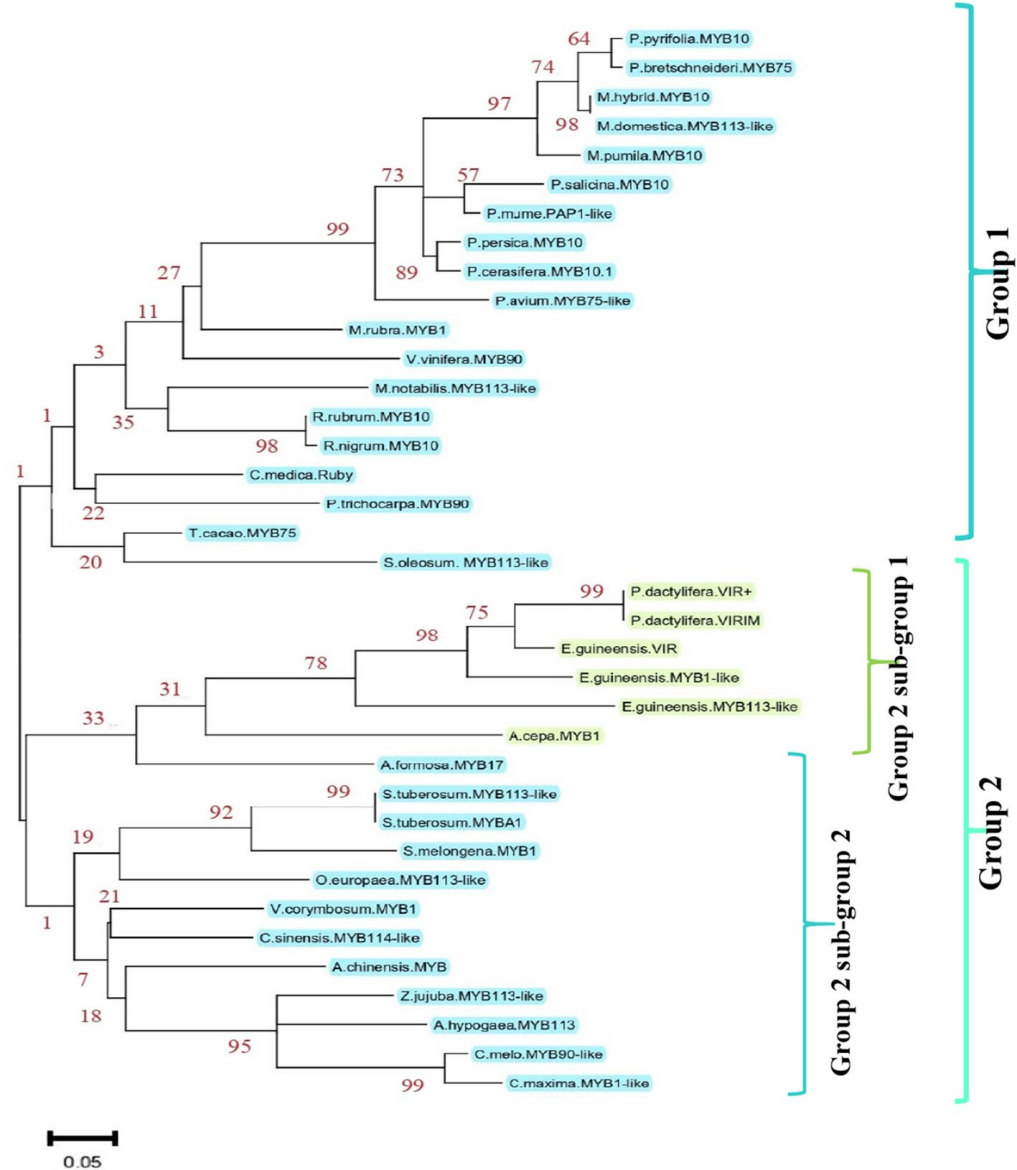
Phylogenetic relationships of the date palm and 32 plant species based on the R2R3-MYB gene product. GenBank accession numbers of various species and alignments are given in Table S4 and Figure S5. The sky-blue blocked species are dicots, and the light green ones are monocots.

The R2R3-MYB-like protein sequence of the cultivar Ajwah (*VIR*^+^) was searched within the NCBI blast database, and the first 33 plant species, including the date palm and oil palm, represented monocots, including A*.cepa.MYB1*. The species were divided into two main groups: group 1, comprised dicots, and group 2, which had two subgroups, S1 and S2; S1 comprised date palm *VIR*^+^, *VIR*^*IM*^, monocots, and one dicot. The closest member to the *VIR* gene of the date palm was the *VIR* and MYB1-like of oil palm^8^. The second closest member to the date palm was the *R2R3-MYB* of onion (*Allium MYB1-like cepa* L.). In addition, the onion *MYB1* has been shown to regulate the biosynthesis of anthocyanin^25^. The nearest *R2R3-MYB* gene from the dicots to the monocots was *MYB1-like* from the crimson columbine *Aquilegia formosa* (Figure 5 and S5), and this gene was suggested as a regulator in the pathway of anthocyanin biosynthesis in flowers^55^.

**Figure 6:**
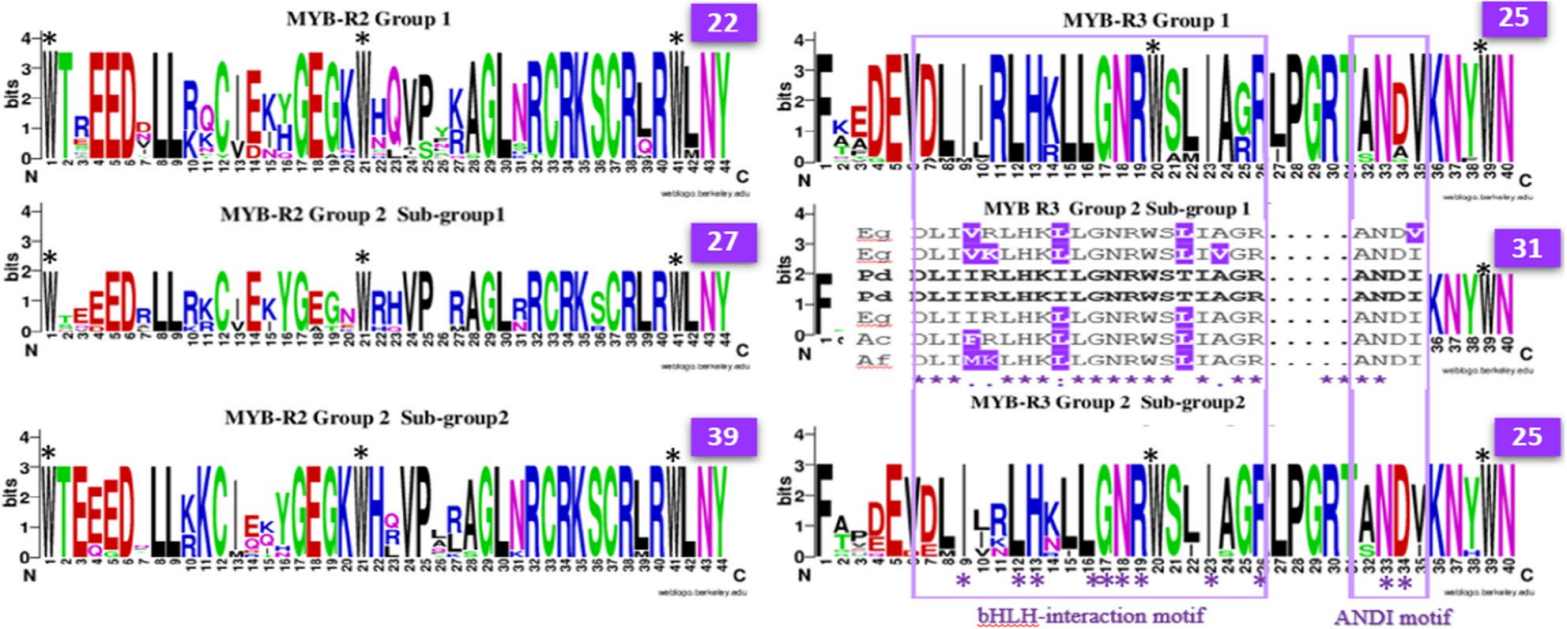
WebLogo shows the conserved amino acids in the DNA binding domains (DBDs) of the R2R3 MYB from different species of groups 1 and 2. Black asterisks denote the conserved amino acid residue tryptophan (W) in the DBDs. Light blue rectangles represent R3 bHLH and ANDI motifs, based on another study^54^. Purple asterisks denote the conserved amino acid in motifs within R3 between the groups. Numbers in purple squares indicate the conserved amino acid number for R2 and R3 in groups 1 and 2. Alignments of the R3 amino acid sequence for the date palm (Pd), oil palm (Eg)*,* onion (Ac), and crimson columbine (Af) were inserted within the WebLogo for subgroup 1 of group 2. Amino acid polymorphisms within the motifs of *VIR*^+^ and *VIR*^*IM*^ for Pd and other species in this subgroup are highlighted in purple.

The alignment with WebLogo identified three conserved W residues in R2 and two in R3, respectively, in all of the species, regardless of grouping. The calculated conserved amino acid percentages for group 1, subgroup 1, and subgroup 2 were 50%, 61%, and 68%, respectively, for R1 and R2, and for R3 were 25%, 31%, and 25%, respectively (Fig. 6).

The bHLH motif of R3 showed a 45% content of conserved amino acids between group 1 and the two subgroups of group 2. However, a higher content of 75% conserved amino acids was calculated for bHLH for the plant species in subgroup 1 compared to those in group 2 (Fig. 6). Additionally, two conserved amino acids were found in the second motif, i.e., the ANDI motif of R3 (Fig. 6), in all of the aligned species. The amino acid sequences of both R3 motifs for *VIR*^+^ and *VIR*^*IM*^ were compared for orthologs in subgroup 1 of group 2. The closest *VIR* gene in the bHLH motif was MYB1-like for the oil palm. Meanwhile, the ANDI motif was conserved in all members of this subgroup except for the oil palm MYB113-like (Fig. 6).

## Discussion

The dates at the three ripening stages of Khalal, Rutab, and Tamar contain different moisture contents, i.e., 50%, 30 to 35%, and 10 to 30%, respectively. The mesocarp usually shrinks when the dates ripen from the Khalal to Tamar stages, due to a reduction in moisture content^37^. In this study, the cultivar Khalas showed a higher reduction in MW than Labana, indicating an overall shrinkage in WMH (Fig. 2). Meanwhile, the colour of the pericarp (skin) determines whether a date is to be used as fresh fruit or processed as dried food. However, in Labana, the yellow colour persisted as patches in the exocarp at the Rutab and Tamar stages, with brown colouration as patches on the outer part of the mesocarp (Fig. 2). Tamar is the longest ripening stage and starts with the soft phase. However, dates could be left for a longer time on palms to become semi-dried or dried.

A recent molecular model^23^ suggested that date palm colour was regulated by three alleles: the red wild-type *VIR*^+^, the yellow *VIR*^*IM*^, and *VIR*^*saf*^. The *VIR*^*IM*^ allele introduced a premature stop in exon three due to an insertion of an LTR-RT, while the *VIR*^*saf*^ allele interrupted the start code. These mutations caused a change in pericarp colour from red to yellow. Meanwhile, the VIR protein that regulates the anthocyanin biosynthesis in plants belongs to the TFs of the MYB and bHLH families^56–58^. Although these MYB proteins vary functionally in eukaryotes, they primarily comprise two conserved DBDs, i.e., R2 and R3^59^. Interestingly, the orthologs of date palm R2R3-MYB with similar functions are also identified in other fruit trees, such as oil palm^8^, grape^60^, apple^61^, and citrus^62^.

In this study, the complete *IM* LTR-RT of the *VIR*^*IM*^ was sequenced for four yellow-fruited cultivars, Rabiah, Labana, Rothanah, and Baydh, with fragment sizes ranging from 386 to 476 bp. In addition, the entire *VIR*^*IM*^ gene was sequenced for Rabiah (2125 bp) and Labana (2048 bp) only. The remaining partially sequenced yellow-fruited cultivars (Khalas, Sukkary, and male Rabiah), may have extended the *IM* retrotransposon insertion sequence found in the BC4 Male^23^ cultivar. The length variation in the LTR-RT may suggest an evolutionary role^63^.

Date palm genes are mostly heterozygous. In this study, the homozygosity of the *VIR* gene was confined to the red date cultivars (*VIR*^+^*/VIR*^+^) and four yellow-fruited cultivars (*VIR*^*IM*^*/VIR*^*IM*^), i.e., Sukkary, Baydh, Khalas, and the male Rabiah. Heterozygosity was identified in Labana, the female Rabiah, and Rothanah. Strangely, none of the yellow cultivars examined in this study had the *VIR*^*saf*^ allele that was identified in another study^23^, with the start codon ATG mutated to ATA^23^. It is possible that increasing the sequence number (sample size) of each cultivar might enhance the identification of heterozygosity in these cultivars, especially those with light-coloured fruits, such as Shalaby and Labana, as found for similarly coloured cultivars in another published work^11^.

The sequence alignment of the amino acids among the 12 cultivars in this study revealed two changes within the wild-type allele (*VIR*^+^*/VIR*^+^) of the dark red Ajwah, Anbarah, and Safawi cultivars. The first amino acid alteration happened at position 187 in exon 3, with K in the dark red-coloured cultivar but E in the other light red-coloured cultivars. This alteration might be related to the accumulation or stability of anthocyanin biosynthesis, suggesting that, besides R2 and R3, other segments of the *VIR*^+^ allele might also be crucial in regulating its expression. In particular, when serving as TF genes, some MYB proteins might contain intrinsically disordered regions (IDRs)^64^ outside of the DBD motifs.

The second amino acid alteration happened at position 224, where V changed to I in exon 3 of *VIR*^+^ in the Anbarah and Safawi cultivars*.* Other red-fruited cultivars, including Ajwah, which yielded dark red dates at the Khalal stage and black fruits at Tamar, had V at this position. In general, the amino acid changes in *VIR*^+^ occurred at the IDR region with three motifs in the C-terminus activating the anthocyanin in the R2-R3-MYBs region of subgroup 6 (S6). These motifs comprised a mixture of hydrophobic and acidic amino acids in relatively good order^51^. In general, hydrophobic amino acids contribute to protein core stabilisation. By contrast, no amino acid alteration occurred in AcMYB110, VvMYBBA1r, and MdMYB10^51^.

The amino acid position of 187 was located right after S6B, which began at amino acid 172 and ended at 186 (Fig. 4). The IUPred score was 0.38 (i.e., < 0.5) for K in Ajwah, Anbarah, and Safawi, and 0.41 for E in the other *VIR*^+^ cultivars, i.e., Jebeli, Hilwah, and Shalaby (Table S4). This position and this score might indicate the possible importance of this amino acid in S6B, even though it is right after S6B, when compared to the selected R2-R3-MYB from the S6 group^51^. However, the amino acid at position 224 was included in S6C of Anbarah and Safawi, which began from position 217 and ended at 233 of the date palm (Figs. 4 and S4). It also had the semi-conserved W^51^ at position 227 in all of the *VIR*^+^ alleles sequenced in this study (Fig. 4). The motif S6A was conserved in all of the *VIR*^+^ and *VIR*^*IM*^ cultivars, except for one amino acid in the published Khalas^40^ and BC4 Male^23^ sequences, at position 136. This amino acid (136) was also found in other orthologs, i.e., the R2R3 MYB of the strong and moderate anthocyanin activities, AcMYB110, AcMYB310, and MdMYB10^51^.

The alignment of the R2R3-MYB orthologous proteins in 32 plant species from both monocots and dicots (Table S4; Figure S5) with protein sequences of wild-type (*VIR*^+^) and mutant (*VIR*^*IM*^) alleles showed similarity in the R2 and R3 motifs. Interestingly, the alteration of I in the S6C of Anbarah and Safawi *VIR*^+^ (Fig. 4) was also identified in some plant species, such as the purple potato *Solanum tuberosum* L. (NCBI gene ID: KP317177) and the eggplant *S. melongena* L. (NCBI gene ID: KT259043.1)^31^. However, the amino acid at position 187, located right after S6B, was unique to the date palm in all of the dark red-coloured cultivars. Expectedly, the monocots would be assigned to a group and a subgroup.

The sequence comparison among the wild-type *VIR*^+^, *IM* retrotransposon *VIR*^*IM*^, Khenezi (red), Lulu (yellow)^11^, Khalas (yellow)^40^, BC4 Male (yellow)^23^, wild-type oil palm allele (*Nigrescens*), and mutant alleles^8^ revealed high similarity at the amino acid levels between the date palm and oil palm. Interestingly, there were changes in the amino acids in both the R2 and R3 MYB motifs in the date palm and oil palm. Specifically, four different amino acids were identified between the date and oil palms in the R2 and only two in the R3 motifs of VIR MYB. However, these two motifs were conserved within the same species (Figure S4).

## Conclusion

In this study, ripening at the Rutab and Tamar stages differed with regard to the spread of the dark colour and mesocarp firmness. The LTR-RT insertion at exon 3 of the *VIR*^*IM*^ varied in size in some of the sequenced cultivars. The C-terminus motifs S6A, S6B, and S6C were found in the VIR^+^ protein sequence. The protein alignment of the different cultivars suggested an alteration of the amino acid in the dark-coloured dates outside of the R2 and R3 domains, and it was located immediately after S6B. The amino acid had a lower binding score, suggesting that it was relatively ordered with a crucial role in anthocyanin regulation and accumulation. Understanding the genetic code of anthocyanin biosynthesis and accumulation in date palm cultivars might contribute to our understanding of fruit colour variation, which might impact the importance of this palm as a nutrient source.

### Supplementary Information

Supplementary Information.

## Data Availability

All nucleotide sequences have been deposited in NCBI GenBank and can be found under accession numbers No: MN587858.1, MN587859.1, MN587860.1, MN587861.1, MN587862.1, MN587863.1, MN587864.1, MN587865.1, MN587866.1, MN587867.1, MN587868.1, MN587869.1, MN587870.1 (https://www.ncbi.nlm.nih.gov/popset/?term=2171233756).
