# Supplementary material for: Molecular insights into the VIRESCENS amino acid sequence and its implication in anthocyanin production in red- and yellow-fruited cultivars of date palm
Source: Sci Rep. 2023 Nov 24;13:20688. doi: 10.1038/s41598-023-47604-9 (PMC10673830; doi:10.1038/s41598-023-47604-9)
Supplement: Supplementary file 1 — Supplementary Information. [file 41598_2023_47604_MOESM1_ESM.docx]

**Supplemental Material**

**Table S1**. VIR gene primers ID used, nucleotide sequences, and advised annealing temperature by Macrogen Inc, South Korea.

| # | Primer ID | Sequence | Annealing temperature ℃ |
| --- | --- | --- | --- |
| 1 | DPVIRF1 | 5' - AAG TTT TGG CCT CAA TGG G - 3' | 55.2℃ |
| 2 | DPVIRR1 | 5' - CTG AAG AGA TGC GAG AAT - 3' | 51.6℃ |
| 3 | DPVIRF2 | 5' – ATT CTC GGA TCT CTT CAG - 3' | 51.6℃ |
| 4 | DPVIRR2 | 5' - CCT GAT TGA CCG AAT TCA G - 3' | 55.2℃ |
| 5 | DPVIRF3 | 5' - CTG AAT TCG GTC AAT CAG G - 3' | 55.2℃ |
| 6 | DPVIRR3R | 5' - ATG GTG GCT AAG CAA GTC G - 3' | 57.3℃ |
| 7 | DPVIRR3Y | 5' – GAA GAC ATA TTG AAT ACG - 3' | 47.0℃ |

**Table S2.** ANOVA One-Way analysis of variance for width measurements of dates at mid-height and mesocarp.

| Source | DF | Khalas | | | | Labana | | | |
| --- | --- | --- | --- | --- | --- | --- | --- | --- | --- |
|  |  | **Width Mid Height** | | **Mesocarp width** | | **Width Mid Height** | | **Mesocarp width** | |
|  |  | **F-Value** | **P-Value** | **F-Value** | **P-Value** | **F-Value** | **P-Value** | **F-Value** | **P-Value** |
| Ripening stage | 2 | 22.16 | 0.000 | 191.25 | 0.000 | 13.55 | 0.000 | 25.37 | 0.000 |
| Halves | 5 | 0.59 | 0.814 | 0.79 | 0.651 | 0.11 | 0.989 | 0.37 | 0.866 |

Note: Significant level α = 0.05.

**Table S3.** Primer sets used for VIR genotyping heterogenicity identification

| Cultivar | Primer set | PCR product | +\- | Genotype |
| --- | --- | --- | --- | --- |
| Ajwah | DPVIRF1-DPVIRR1 | 2 | + | *VIR^+^\VIR^+^* |
|  | DPVIRF2-DPVIRR2 | 2^*^ | + |  |
|  | DPVIRF2-DPVIRR3R | 2 | + |  |
|  | DPVIRF2-DPVIRR3Y | 0 | - |  |
|  | DPVIRF3-DPVIRR3Y | 0 | - |  |
| Anbarah | DPVIRF1-DPVIRR1 | 2 | + | *VIR^+^\VIR^+^* |
|  | DPVIRF2-DPVIRR2 | 2^*^ | + |  |
|  | DPVIRF2-DPVIRR3R | 2 | + |  |
|  | DPVIRF2-DPVIRR3Y | 0 | - |  |
|  | DPVIRF3-DPVIRR3Y | 0 | - |  |
| Safawi | DPVIRF1-DPVIRR1 | 3 | + | *VIR^+^\VIR^+^* |
|  | DPVIRF2-DPVIRR2 | 3^*^ | + |  |
|  | DPVIRF2-DPVIRR3R | 3 | + |  |
|  | DPVIRF2-DPVIRR3Y | 0 | - |  |
|  | DPVIRF3-DPVIRR3Y | 0 | - |  |
| Jebeli | DPVIRF1-DPVIRR1 | 3 | + | *VIR^+^\VIR^+^* |
|  | DPVIRF2-DPVIRR2 | 3^*^ | + |  |
|  | DPVIRF2-DPVIRR3R | 3 | + |  |
|  | DPVIRF2-DPVIRR3Y | 0 | - |  |
|  | DPVIRF3-DPVIRR3Y | 0 | - |  |
| Hilwah | DPVIRF1-DPVIRR1 | 2 | + | *VIR^+^\VIR^+^* |
|  | DPVIRF2-DPVIRR2 | 2^*^ | + |  |
|  | DPVIRF2-DPVIRR3R | 2 | + |  |
|  | DPVIRF2-DPVIRR3Y | 0 | - |  |
|  | DPVIRF3-DPVIRR3Y | 0 | - |  |
| Shalaby | DPVIRF1-DPVIRR1 | 3 | + | *VIR^+^\VIR^+^* |
|  | DPVIRF2-DPVIRR2 | 3^*^ | + |  |
|  | DPVIRF2-DPVIRR3R | 3 | + |  |
|  | DPVIRF2-DPVIRR3Y | 0 | - |  |
|  | DPVIRF3-DPVIRR3Y | 0 | - |  |
| Rothanah | DPVIRF1-DPVIRR1 | 3 | + | *VIR^+^/VIR^IM^* |
|  | DPVIRF2-DPVIRR2 | 3^*^ | + |  |
|  | DPVIRF2-DPVIRR3R | 3 | + |  |
|  | DPVIRF2-DPVIRR3Y | 3 | + |  |
|  | DPVIRF3-DPVIRR3Y | 2^*^ | + |  |
| Rabiah | DPVIRF1-DPVIRR1 | 3 | + | *VIR^+^/VIR^IM^* |
|  | DPVIRF2-DPVIRR2 | 3^*^ | + |  |
|  | DPVIRF2-DPVIRR3R | 3 | + |  |
|  | DPVIRF2-DPVIRR3Y | 3 | + |  |
|  | DPVIRF3-DPVIRR3Y | 3^*^ | + |  |
| Labana | DPVIRF1-DPVIRR1 | 2 | + | *VIR^+^/VIR^IM^* |
|  | DPVIRF2-DPVIRR2 | 2 | + |  |
|  | DPVIRF2-DPVIRR3R | 2 | + |  |
|  | DPVIRF2-DPVIRR3Y | 1^*^ | + |  |
|  | DPVIRF3-DPVIRR3Y | 2 | + |  |
| Sukkary | DPVIRF1-DPVIRR1 | 3 | + | *VIR^IM^/VIR^IM^* |
|  | DPVIRF2-DPVIRR2 | 1^*^ | + |  |
|  | DPVIRF2-DPVIRR3R | 0 | - |  |
|  | DPVIRF2-DPVIRR3Y | 3 | + |  |
|  | DPVIRF3-DPVIRR3Y | 1^*^ | + |  |
| Baydh | DPVIRF1-DPVIRR1 | 3 | + | *VIR^IM^/VIR^IM^* |
|  | DPVIRF2-DPVIRR2 | 1^*^ | + |  |
|  | DPVIRF2-DPVIRR3R | 3 | - |  |
|  | DPVIRF2-DPVIRR3Y | 3 | + |  |
|  | DPVIRF3-DPVIRR3Y | 1^*^ | + |  |
| Khalas | DPVIRF1-DPVIRR1 | 3 | + | *VIR^IM^/VIR^IM^* |
|  | DPVIRF2-DPVIRR2 | 3 | + |  |
|  | DPVIRF2-DPVIRR3R | 0 | - |  |
|  | DPVIRF2-DPVIRR3Y | 1^*^ | + |  |
|  | DPVIRF3-DPVIRR3Y | 2 | + |  |
| Male | DPVIRF1-DPVIRR1 | 2 | + | *VIR^IM^/VIR^IM^* |
|  | DPVIRF2-DPVIRR2 | 3 | + |  |
|  | DPVIRF2-DPVIRR3R | 0 | - |  |
|  | DPVIRF2-DPVIRR3Y | 1^*^ | + |  |
|  | DPVIRF3-DPVIRR3Y | 3 | + |  |

**Note**: PCR products without Asterisks (*) sequenced in both directions. PCR products with Asterisks (*) did not use during sequence.

P.dactylifera.C.Khenezi** ATGGGTAATTTGCGTGCTGAAGTCCAACGTCCCGNNGTTCGCAAAGGTGCATGGACTGAA 60

P.dactylifera.C.Ajwah ATGGGTAATTTGCGTGCTGAAGTCCAACGTCCCGGTGTTCGCAAAGGTGCATGGACTGAA 60

P.dactylifera.C.Hilwah ATGGGTAATTTGCGTGCTGAAGTCCAACGTCCCGGTGTTCGCAAAGGTGCATGGACTGAA 60

P.dactylifera.C.Shalaby ATGGGTAATTTGCGTGCTGAAGTCCAACGTCCCGGTGTTCGCAAAGGTGCATGGACTGAA 60

P.dactylifera.C.Anbarah ATGGGTAATTTGCGTGCCGAAGTCCAACGTCCCGGTGTTCGCAAAGGTGCATGGACTGAA 60

P.dactylifera.C.Safawi ATGGGTAATTTGCGTGCCGAAGTCCAACGTCCCGGTGTTCGCAAAGGTGCATGGACTGAA 60

P.dactylifera.C.Jebeli ATGGGTAATTTGCGTGCCGAAGTCCAACGTCCCGGTGTTCGCAAAGGTGCATGGACTGAA 60

***************** **************** ************************

P.dactylifera.C.Khenezi** GAGGAGGACAGGCTTCTAAGGAAGTGCATTGAGATATATGGCGAAGGGAACTGGCGCCAT 120

P.dactylifera.C.Ajwah GAGGAGGACAGGCTTCTAAGGAAGTGCATTGAGATATATGGCGAAGGGAACTGGCGCCAT 120

P.dactylifera.C.Hilwah GAGGAGGACAGGCTTCTAAGGAAGTGCATTGAGATATATGGCGAAGGGAACTGGCGCCAT 120

P.dactylifera.C.Shalaby GAGGAGGACAGGCTTCTAAGGAAGTGCATTGAGATATATGGCGAAGGGAACTGGCGCCAT 120

P.dactylifera.C.Anbarah GAGGAGGACAGGCTTCTAAGGAAGTGCATTGAGATATATGGCGAAGGGAACTGGCGCCAT 120

P.dactylifera.C.Safawi GAGGAGGACAGGCTTCTAAGGAAGTGCATTGAGATATATGGCGAAGGGAACTGGCGCCAT 120

P.dactylifera.C.Jebeli GAGGAGGACAGGCTTCTAAGGAAGTGCATTGAGATATATGGCGAAGGGAACTGGCGCCAT 120

************************************************************

P.dactylifera.C.Khenezi** GTTCCCGAAAGGGCAGGTAAGCGACTCCTTATAGAGAGTGGAGTCTTTTATCATTCTTAA 180

P.dactylifera.C.Ajwah GTTCCCGAAAGGGCAGGTAAGCGACTCCTTATAGAGAGTGGGGTCTTTTATCATTCTTAA 180

P.dactylifera.C.Hilwah GTTCCCGAAAGGGCAGGTAAGCGACTCCTTATAGAGAGTGGGGTCTTTTATCATTCTTAA 180

P.dactylifera.C.Shalaby GTTCCCGAAAGGGCAGGTAAGCGACTCCTTATAGAGAGTGGGGTCTTTTATCATTCTTAA 180

P.dactylifera.C.Anbarah GTTCCCGAAAGGGCAGGTAAGCGACTCCTTATAGAGAGTGGAGTCTTTTATCATTCTTAA 180

P.dactylifera.C.Safawi GTTCCCGAAAGGGCAGGTAAGCGACTCCTTATAGAGAGTGGAGTCTTTTATCATTCTTAA 180

P.dactylifera.C.Jebeli GTTCCCGAAAGGGCAGGTAAGCGACTCCTTATAGAGAGTGGGGTCTTTTATCATTCTTAA 180

***************************************** ******************

P.dactylifera.C.Khenezi** GCTTACTCTGCTTTTGTGGGTTTGAAGGCCTCAGAAGGTGCCGAAAGAGCTGCCGATTGC 240

P.dactylifera.C.Ajwah GCTTACTCTGCTTTTGTGGGTTTGAAGGCCTCAGAAGGTGCCGAAAGAGCTGCCGATTGC 240

P.dactylifera.C.Hilwah GCTTACTCTGCTTTTGTGGGTTTGAAGGCCTCAGAAGGTGCCGAAAGAGCTGCCGATTGC 240

P.dactylifera.C.Shalaby GCTTACTCTGCTTTTGTGGGTTTGAAGGCCTCAGAAGGTGCCGAAAGAGCTGCCGATTGC 240

P.dactylifera.C.Anbarah GCTTACTCTGCTTTTGTGGGTTTGAAGGCCTCAGAAGGTGCCGAAAGAGCTGCCGATTGC 240

P.dactylifera.C.Safawi GCTTACTCTGCTTTTGTGGGTTTGAAGGCCTCAGAAGGTGCCGAAAGAGCTGCCGATTGC 240

P.dactylifera.C.Jebeli GCTTACTCTGCTTTTGTGGGTTTGAAGGCCTCAGAAGGTGCCGAAAGAGCTGCCGATTGC 240

************************************************************

P.dactylifera.C.Khenezi** GTTGGTTGANCTATCTCTGCCCCAGGATCAACAGGGAGAAATTTTCTGAGGAAGAAACAG 300

P.dactylifera.C.Ajwah GTTGGTTGAACTATCTCTGCCCCAGGATCAACAGGGAGAAATTTTCTGAGGAAGAAACAG 300

P.dactylifera.C.Hilwah GTTGGTTGAACTATCTCTGCCCCAGGATCAACAGGGAGAAATTTTCTGAGGAAGAAACAG 300

P.dactylifera.C.Shalaby GTTGGTTGAACTATCTCTGCCCCAGGATCAACAGGGAGAAATTTTCTGAGGAAGAAACAG 300

P.dactylifera.C.Anbarah GTTGGTTGAACTATCTCTGCCCCAGGATCAACAGGGAGAAATTTTCTGAGGAAGAAACAG 300

P.dactylifera.C.Safawi GTTGGTTGAACTATCTCTGCCCCAGGATCAACAGGGAGAAATTTTCTGAGGAAGAAACAG 300

P.dactylifera.C.Jebeli GTTGGTTGAACTATCTCTGCCCCAGGATCAACAGGGAGAAATTTTCTGAGGAAGAAACAG 300

********* **************************************************

P.dactylifera.C.Khenezi** ATCTTATCATTAGGCTTCATAAGATCTTGGGCAACAGGTAACAACAGACTATAAAGATTG 360

P.dactylifera.C.Ajwah ATCTTATCATTAGGCTTCATAAGATCTTGGGCAACAGGTAACAACAGACTATAAAGATTG 360

P.dactylifera.C.Hilwah ATCTTATCATTAGGCTTCATAAGATCTTGGGCAACAGGTAACAACAGACTATAAAGATTG 360

P.dactylifera.C.Shalaby ATCTTATCATTAGGCTTCATAAGATCTTGGGCAACAGGTAACAACAGACTATAAAGATTG 360

P.dactylifera.C.Anbarah ATCTTATCATTAGGCTTCATAAGATCTTGGGCAACAGGTAACAACAGACTATAAAGATTG 360

P.dactylifera.C.Safawi ATCTTATCATTAGGCTTCATAAGATCTTGGGCAACAGGTAACAACAGACTATAAAGATTG 360

P.dactylifera.C.Jebeli ATCTTATCATTAGGCTTCATAAGATCTTGGGCAACAGGTAACAACAGACTATAAAGATTG 360

************************************************************

P.dactylifera.C.Khenezi** CCTTGGTTACAGTGACCATGTATTTGTTTTCTATCAAGCGATTCCAAAAGTTTCGCTTCC 420

P.dactylifera.C.Ajwah CCTTGGTTACAGTGACCATGTATTTGTTTTCTATCAAGCGATTCCAAAAGTTTCGCTTCC 420

P.dactylifera.C.Hilwah CCTTGGTTACAGTGACCATGTATTTGTTTTCTATCAAGCGATTCCAAAAGTTTCGCTTCC 420

P.dactylifera.C.Shalaby CCTTGGTTACAGTGACCATGTATTTGTTTTCTATCAAGCGATTCCAAAAGTTTCGCTTCC 420

P.dactylifera.C.Anbarah CCTTGGTTACAGTGACCATGTATTTGTTTTCTATCAAGCGATTCCAAAAGTTTCGCTTCC 420

P.dactylifera.C.Safawi CCTTGGTTACAGTGACCATGTATTTGTTTTCTATCAAGCGATTCCAAAAGTTTCGCTTCC 420

P.dactylifera.C.Jebeli CCTTGGTTACAGTGACCATGTATTTGTTTTCTATCAAGCGATTCCAAAAGTTTCGCTTCC 420

************************************************************

P.dactylifera.C.Khenezi** CTTNCCTTCATATTCCTTACCTGAGANAGTATAAGAAAATAACTACGAGATCATTCATTG 480

P.dactylifera.C.Ajwah CTTACCTTCATATTCCTTACCTGAGATAGTATAAGAAAATAACTACGAGATCATTCATTG 480

P.dactylifera.C.Hilwah CTTTCCTTCATATTCCTTACCTGAGATAGTATAAGAAAATAACTACGAGATCATTCATTG 480

P.dactylifera.C.Shalaby CTTTCCTTCATATTCCTTACCTGAGATAGTATAAGAAAATAACTACGAGATCATTCATTG 480

P.dactylifera.C.Anbarah CTTTCCTTCATATTCCTTACCTGAGATAGTATAAGAAAATAACTACGAGATCATTCATTA 480

P.dactylifera.C.Safawi CTTTCCTTCATATTCCTTACCTGAGATAGTATAAGAAAATAACTACGAGATCATTCATTG 480

P.dactylifera.C.Jebeli CTTTCCTTCATATTCCTTACCTGAGATAGTATAAGAAAATAACTACGAGATCATTCATTA 480

*** ********************** ********************************

P.dactylifera.C.Khenezi** TATACATGTTTCTTTTAGTGCAGCTGTCAGCTCTCGTGGTTGGCACCTCTCTCCTAAGGA 540

P.dactylifera.C.Ajwah TATACATGTTTCTTTTAGTGCAGCTGTCAGCTCTCGTGGTTGGCACCTCTCTCCTAAGGA 540

P.dactylifera.C.Hilwah TATACATGTTTCTTTTAGTGCAGCTGTCAGCTCTCGTGGTTGGCACCTCTCTCCTAAGGA 540

P.dactylifera.C.Shalaby TATACATGTTTCTTTTAGTGCAGCTGTCAGCTCTCGTGGTTGGCACCTCTCTCCTAAGGA 540

P.dactylifera.C.Anbarah TATACATGTTTCTTTTAGTGCAGCTGTCAGCTCTCGTGGTTGGCACCTCTCTCCTAAGGA 540

P.dactylifera.C.Safawi TATACATGTTTCTTTTAGTGCAGCTGTCAGCTCTCGTGGTTGGCACCTCTCTCCTAAGGA 540

P.dactylifera.C.Jebeli TATACATGTTTCTTTTAGTGCAGCTGTCAGCTCTCGTGGTTGGCACCTCTCTCCTAAGGA 540

************************************************************

P.dactylifera.C.Khenezi** GTTGTCTTTATGGCATATAAAGTTCAAATCGTAAATAAAGTTAGTTTTCAAAAAAATACN 600

P.dactylifera.C.Ajwah GTTGTCTTTATGGCATATAAAGTTCAAATCGTAAATAAAGTTAGTTTTCAAAAAAATACG 600

P.dactylifera.C.Hilwah GTTGTCTTTATGGCATATAAAGTTCAAATCGTAAATAAAGTTAGTTTTCAAAAAAATACG 600

P.dactylifera.C.Shalaby GTTGTCTTTATGGCATATAAAGTTCAAATCGTAAATAAAGTTAGTTTTCAAAAAAATACG 600

P.dactylifera.C.Anbarah GTTGTCTTTATGGCATATAAAGTTCAAATCGTAAATAAAGTTAGTTTTCAAAAAAATACA 600

P.dactylifera.C.Safawi GTTGTCTTTATGGCATATAAAGTTCAAATCGTAAATAAAGTTAGTTTTCAAAAAAATACA 600

P.dactylifera.C.Jebeli GTTGTCTTTATGGCATATAAAGTTCAAATCGTAAATAAAGTTAGTTTTCAAAAAAATACA 600

***********************************************************

P.dactylifera.C.Khenezi** AAAAGATATGATATGTTTAAAACATTATGTATTTTTAAAATTCTCGCATCTCTTCAGTTT 660

P.dactylifera.C.Ajwah AAAAGATATGATATGTTTAAAACATTATGTATTTTTAAAATTCTCGGATCTCTTCAGTTT 660

P.dactylifera.C.Hilwah AAAAGATATGATATGTTTAAAACATTATGTATTTTTAAAATTCTCGCATCTCTTCAGTTT 660

P.dactylifera.C.Shalaby AAAAGATATGATATGTTTAAAACATTATGTATTTTTAAAATTCTCGCATCTCTTCAGTTT 660

P.dactylifera.C.Anbarah AAAAGATATGGTATGTTTAAAACATTATGTATTTTTAAAATTCTCGGATCTCTTCAGTTT 660

P.dactylifera.C.Safawi AAAAGATATGGTATGTTTAAAACATTATGTATTTTTAAAATTCTCGGATCTCTTCAGTTT 660

P.dactylifera.C.Jebeli AAAAGATATGGTATGTTTAAAACATTATGTATTTTTAAAATTCTCGGATCTCTTCAGTTT 660

********** *********************************** *************

P.dactylifera.C.Khenezi** TCCAAATAGAGTCTATAAANANACTTCTCTANANATTAATTCAAAAAAACAATCTGATNA 720

P.dactylifera.C.Ajwah TCCAAATAGAGTCTATAAAAACACTTCTCTAGGATTAATTCAAAAAAACAATCTGATCAT 720

P.dactylifera.C.Hilwah TCCAAATAGAGTCTATAAAAACACTTCTCTAGGATTAATTCAAAAAAACAATCTGATCAT 720

P.dactylifera.C.Shalaby TCCAAATAGAGTCTATAAAAACACTTCTCTAGGATTAATTCAAAAAAACAATCTGATCAT 720

P.dactylifera.C.Anbarah TCCAAATAGAGTCTATAAAAACACTTCTCTAGGATTAATTCAAAAAAACAATCTGACTAT 720

P.dactylifera.C.Safawi TCCAAATAGAGTCTATAAATATACTTCTCTAGGATTAATTCAAAAAAACAATCTGATCAT 720

P.dactylifera.C.Jebeli TCCAAATAGAGTCTATAAATATACTTCTCTAGGATTAATTCAAAAAAACAATCTGATCAT 720

******************* * ********* * * * ****** *

P.dactylifera.C.Khenezi** TTTTCTTTTTTACTGNACTCTGTCTTCTGAGGATTAATTCACTGNTACTCTTTCTCACTG 780

P.dactylifera.C.Ajwah TTTCTTTTTAACTGGTACTCTGTCTTCTGAGGATTAATTCACTGCTACTCTTTCTCACTG 780

P.dactylifera.C.Hilwah TTTCTTTTTTACTGGTACTCTGTCTTCTGAGGATTAATTCAATGCTACTCTTTCTCACTG 780

P.dactylifera.C.Shalaby TTTCTTTTTTACTGGTACTCTGTCTTCTGAGGATTAATTCAATGCTACTCTTTCTCACTG 780

P.dactylifera.C.Anbarah TTTCTTTTTAACTGGTACTCTGTCTTCTGAGGATTAATTCACTGCTACTCTTTCTCACTG 780

P.dactylifera.C.Safawi TTTCTTTTTTACTGGTACTCTGTCTTCTGAGGATTAATTCACTGCTACTCTTTCTCACTG 780

P.dactylifera.C.Jebeli TTTCTTTTTAACTGGTACTCTGTCTTCTGAGGATTAATTCACTGCTACTCTTTCTCACTG 780

*** **** * ************************* ** ***************

P.dactylifera.C.Khenezi** CTAATTTGAGCAAACGAGACCTTGCTCCAAGATTCAGANAAAGGGGCTGCTCATAAAATT 840

P.dactylifera.C.Ajwah CTAATTTGAGCAAACGAGACCTTGCTCCAAGATTCAGACAAAGGGGCTGCTCATAAAATT 840

P.dactylifera.C.Hilwah CTAATTTGAGCAAACGAGACCTTGCTCCAAGATTCAGACAAAGGGGCTGCTCATAAAATT 840

P.dactylifera.C.Shalaby CTAATTTGAGCAAACGAGACCTTGCTCCAAGATTCAGACAAAGGGGCTGCTCATAAAATT 840

P.dactylifera.C.Anbarah CTAATTTGAGCAAACGAGACCTTGCTCCAAGATTCAGACAAAGGGGCTGCTCATAAAATT 840

P.dactylifera.C.Safawi CTAATTTGAGCAAACGAGACCTTGCTCCAAGATTCAGACAAAGGGGCTGCTCATAAAATT 840

P.dactylifera.C.Jebeli CTAATTTGAGCAAACGAGACCTTGCTCCAAGATTCAGACAAAGGGGCTGCTCATAAAATT 840

************************************** *********************

P.dactylifera.C.Khenezi** CCTTTAGGAAAACTGCAACACATCACCTTCTAATAAAAGGAACCAATGCATTTAAACCTG 900

P.dactylifera.C.Ajwah CCTTTAGGAAAACTGCAACACATCACCTTCTAATAAAAGGAACCAATGCATTTAAACCTG 900

P.dactylifera.C.Hilwah CCTTTAGGAAAACTGCAACACATCACCTTCTAATAAAAGGAACCAATGCATTTAAACCTG 900

P.dactylifera.C.Shalaby CCTTTAGGAAAACTGCAACACATCACCTTCTAATAAAAGGAACCAATGCATTTAAACCTG 900

P.dactylifera.C.Anbarah CCTTTAGGAAAACTGCAACACATCACCTTCTAATAAAAGGAACCAATGCATTTAAACCTG 900

P.dactylifera.C.Safawi CCTTTAGGAAAACTGCAACACATCACCTTCTAATAAAAGGAACCAATGCATTTAAACCTG 900

P.dactylifera.C.Jebeli CCTTTAGGAAAACTGCAACACATCACCTTCTAATAAAAGGAACCAATGCATTTAAACCTG 900

************************************************************

P.dactylifera.C.Khenezi** NCCAGAGGAAGTTGTCTTTTGGGCCTAAAGAAAATAAGTGATAATATCAACACATCTTCA 960

P.dactylifera.C.Ajwah TCCAGAGGAAGTTGTCTTTTGGGCCTAAAGAAAATAAGTGATAATATCAACACATCTTCA 960

P.dactylifera.C.Hilwah TCCAGAGGAAGTTGTCTTTTGGGCCTAAAGAAAATAAGTGATAATATCAACACATCTTCA 960

P.dactylifera.C.Shalaby TCCAGAGGAAGTTGTCTTTTGGGCCTAAAGAAAATAAGTGATAATATCAACACATCTTCA 960

P.dactylifera.C.Anbarah TCCAGAGGAAGTTGTCTTTTGGGCCTAAAGAAAATAAGTGATAATATCAACACATCTTCA 960

P.dactylifera.C.Safawi TCCAGAGGAAGTTGTCTTTTGGGCCTAAAGAAAATAAGTGATAATATCAACACATCTTCA 960

P.dactylifera.C.Jebeli TCCAGAGGAAGTTGTCTTTTGGGCCTAAAGAAAATAAGTGATAATATCAACACATCTTCA 960

***********************************************************

P.dactylifera.C.Khenezi** GTCTATTTTTCCCGTACAATTTTTTTGGAATCTCCACCCCATTTTTTTCATACAATTGTA 1020

P.dactylifera.C.Ajwah GTCTATTTTTCCCGTACAATTTTTTTGGAATCTCCACCCCATTTTTTTCATACAATTGTA 1020

P.dactylifera.C.Hilwah GTCTATTTTTCCCGTACAATTTTTTTGGAATCTCCACCCCATTTTTTTCATACAATTGTA 1020

P.dactylifera.C.Shalaby GTCTATTTTTCCCGTACAATTTTTTTGGAATCTCCACCCCATTTTTTTCATACAATTGTA 1020

P.dactylifera.C.Anbarah GTCTATTTTTCCCGTACAATTTTTTTGGAATCTCCACCCCATTTTTTTCATACAATTGTA 1020

P.dactylifera.C.Safawi GTCTATTTTTCCCGTACAATTTTTTTGGAATCTCCACCCCATTTTTTTCATACAATTGTA 1020

P.dactylifera.C.Jebeli GTCTATTTTTCCCGTACAATTTTTTTGGAATCTCCACCCCATTTTTTTCATACAATTGTA 1020

************************************************************

P.dactylifera.C.Khenezi** TCTTTGATTTCATTCTTGAGATCTTTCCACTTACTCCAAATGCAATGGGGTATGCAACCA 1080

P.dactylifera.C.Ajwah TCTTTGATTTCATTCTTGAGATCTTTCCACTTACTCCAAATGCAATGGGGTATGCAACCA 1080

P.dactylifera.C.Hilwah TCTTTGATTTCATTCTTGAGATCTTTCCACTTACTCCAAATGCAATGGGGTATGCAACCA 1080

P.dactylifera.C.Shalaby TCTTTGATTTCATTCTTGAGATCTTTCCACTTACTCCAAATGCAATGGGGTATGCAACCA 1080

P.dactylifera.C.Anbarah TCTTTGATTTCATTCTTGAGATCTTTCCACTTACTCCAAATGCAATGGGGTATGCAACCA 1080

P.dactylifera.C.Safawi TCTTTGATTTCATTCTTGAGATCTTTCCACTTACTCCAAATGCAATGGGGTATGCAACCA 1080

P.dactylifera.C.Jebeli TCTTTGATTTCATTCTTGAGATCTTTCCACTTACTCCAAATGCAATGGGGTATGCAACCA 1080

************************************************************

P.dactylifera.C.Khenezi** CCGGGCTCTAGCAATATTTGCTGTGTAGAGATGTTCGTGTACCGTACTTTTCTTGAATCT 1140

P.dactylifera.C.Ajwah CCGGGCTCTAGCAATATTTGCTGTGTAGAGATGTTCGTGTACCGTACTTTTCTTGAATCT 1140

P.dactylifera.C.Hilwah CCGGGCTCTAGCAATATTTGCTGTGTAGAGATGTTCGTGTACCGTACTTTTCTTGAATCT 1140

P.dactylifera.C.Shalaby CCGGGCTCTAGCAATATTTGCTGTGTAGAGATGTTCGTGTACCGTACTTTTCTTGAATCT 1140

P.dactylifera.C.Anbarah CCGGGCTCTAGCAATATTTGCTGTGTAGAGATGTTCGTGTACCGTACTTTTCTTGAATCT 1140

P.dactylifera.C.Safawi CCGGGCTCTAGCAATATTTGCTGTGTAGAGATGTTCGTGTACCGTACTTTTCTTGAATCT 1140

P.dactylifera.C.Jebeli CCGGGCTCTAGCAATATTTGCTGTGTAGAGATGTTCGTGTACCGTACTTTTCTTGAATCT 1140

************************************************************

P.dactylifera.C.Khenezi** CTCGTCCTGCAAGGGACCCTCTCGTCACAGTTGAACACAAACAATTCAACTAATGAGCTC 1200

P.dactylifera.C.Ajwah CTCGTCCTGCAAGGGACCCTCTCGTCACAGTTGAACACAAACAATTCAACTAATGAGCTC 1200

P.dactylifera.C.Hilwah CTCGTCCTGCAAGGGACCCTCTCGTCACAGTTGAACACAAACAATTCAACTAATGAGCTC 1200

P.dactylifera.C.Shalaby CTCGTCCTGCAAGGGACCCTCTCGTCACAGTTGAACACAAACAATTCAACTAATGAGCTC 1200

P.dactylifera.C.Anbarah CTCGTCCTGCAAGGGACCCTCTCGTCACAGTTGAACACAAACAATTCAACTAATGAGCTC 1200

P.dactylifera.C.Safawi CTCGTCCTGCAAGGGACCCTCTCGTCACAGTTGAACACAAACAATTCAACTAATGAGCTC 1200

P.dactylifera.C.Jebeli CTCGTCCTGCAAGGGACCCTCTCGTCACAGTTGAACACAAACAATTCAACTAATGAGCTC 1200

************************************************************

P.dactylifera.C.Khenezi** TGAATTCGGTCAATCAGGTGGTCAACAATTGCAGGTAGGCTTCCAGGCAGGACAGCAAAT 1260

P.dactylifera.C.Ajwah TGAATTCGGTCAATCAGGTGGTCAACAATTGCAGGTAGGCTTCCAGGCAGGACAGCAAAT 1260

P.dactylifera.C.Hilwah TGAATTCGGTCAATCAGGTGGTCAACAATTGCAGGTAGGCTTCCAGGCAGGACAGCAAAT 1260

P.dactylifera.C.Shalaby TGAATTCGGTCAATCAGGTGGTCAACAATTGCAGGTAGGCTTCCAGGCAGGACAGCAAAT 1260

P.dactylifera.C.Anbarah TGAATTCGGTCAATCAGGTGGTCAACAATTGCAGGTAGGCTTCCAGGCAGGACAGCAAAT 1260

P.dactylifera.C.Safawi TGAATTCGGTCAATCAGGTGGTCAACAATTGCAGGTAGGCTTCCAGGCAGGACAGCAAAT 1260

P.dactylifera.C.Jebeli TGAATTCGGTCAATCAGGTGGTCAACAATTGCAGGTAGGCTTCCAGGCAGGACAGCAAAT 1260

************************************************************

P.dactylifera.C.Khenezi** GACATCAAGAACTACTGGAATTCTCAGCTGAGCAAGAAAGTAGAAGTTGAAAACAAGGAG 1320

P.dactylifera.C.Ajwah GACATCAAGAACTACTGGAATTCTCAGCTGAGCAAGAAAGTAGAAGTTGAAAACAAGGAG 1320

P.dactylifera.C.Hilwah GACATCAAGAACTACTGGAATTCTCAGCTGAGCAAGAAAGTAGAAGTTGAAAACAAGGAG 1320

P.dactylifera.C.Shalaby GACATCAAGAACTACTGGAATTCTCAGCTGAGCAAGAAAGTAGAAGTTGAAAACAAGGAG 1320

P.dactylifera.C.Anbarah GACATCAAGAACTACTGGAATTCTCAGCTGAGCAAGAAAGTAGAAGTTGAAAACAAGGAG 1320

P.dactylifera.C.Safawi GACATCAAGAACTACTGGAATTCTCAGCTGAGCAAGAAAGTAGAAGTTGAAAACAAGGAG 1320

P.dactylifera.C.Jebeli GACATCAAGAACTACTGGAATTCTCAGCTGAGCAAGAAAGTAGAAGTTGAAAACAAGGAG 1320

************************************************************

P.dactylifera.C.Khenezi** GCGGAGCCCAGTGCTGATGCCAAAGTAATTAAGCCACGGCCATGGAGAGTACCGTTGCAG 1380

P.dactylifera.C.Ajwah GCGGAGCCCAGTGCTGATGCCAAAGTAATTAAGCCACGGCCATGGAGAGTACCGTTGCAG 1380

P.dactylifera.C.Hilwah GCGGAGCCCAGTGCTGATGCCAAAGTAATTAAGCCACGGCCATGGAGAGTACCGTTGCAG 1380

P.dactylifera.C.Shalaby GCGGAGCCCAGTGCTGATGCCAAAGTAATTAAGCCACGGCCATGGAGAGTACCGTTGCAG 1380

P.dactylifera.C.Anbarah GCGGAGCCCAGTGCTGATGCCAAAGTAATTAAGCCACGGCCATGGAGAGTACCGTTGCAG 1380

P.dactylifera.C.Safawi GCGGAGCCCAGTGCTGATGCCAAAGTAATTAAGCCACGGCCATGGAGAGTACCGTTGCAG 1380

P.dactylifera.C.Jebeli GCGGAGCCCAGTGCTGATGCCAAAGTAATTAAGCCACGGCCATGGAGAGTACCGTTGCAG 1380

************************************************************

P.dactylifera.C.Khenezi** TGGATTTGGTCAGGCGATCAGCAATCATGTGGAAGTCAGCACCAGCAAGAAGAGTTTGGC 1440

P.dactylifera.C.Ajwah TGGATTTGGTCAGGCGATCAGCAATCATGTGGAAGTCAGCACCAGCAAGAAGAGTTTGGC 1440

P.dactylifera.C.Hilwah TGGATTTGGTCAGGCGATCAGCAATCATGTGGAAGTCAGCACCAGCAAGAAGAGTTTGGC 1440

P.dactylifera.C.Shalaby TGGATTTGGTCAGGCGATCAGCAATCATGTGGAAGTCAGCACCAGCAAGAAGAGTTTGGC 1440

P.dactylifera.C.Anbarah TGGATTTGGTCAGGCGATCAGCAATCATGTGGAAGTCAGCACCAGCAAGAAGAGTTTGGC 1440

P.dactylifera.C.Safawi TGGATTTGGTCAGGCGATCAGCAATCATGTGGAAGTCAGCACCAGCAAGAAGAGTTTGGC 1440

P.dactylifera.C.Jebeli TGGATTTGGTCAGGCGATCAGCAATCATGTGGAAGTCAGCACCAGCAAGAAGAGTTTGGC 1440

************************************************************

P.dactylifera.C.Khenezi** ATACCAGAACTACCGACAATCTGGGAGAATGATGAAGCTTGGCTGAATAGTATAATTAAC 1500

P.dactylifera.C.Ajwah ATACCAGAACTACCGACAATCTGGGAGAATGATGAAGCTTGGCTGAATAGTATAATTAAC 1500

P.dactylifera.C.Hilwah ATACCAGAACTACCGACAATCTGGGAGAATGATGAAGCTTGGCTGAATAGTATAATTAAC 1500

P.dactylifera.C.Shalaby ATACCAGAACTACCGACAATCTGGGAGAATGATGAAGCTTGGCTGAATAGTATAATTAAC 1500

P.dactylifera.C.Anbarah ATACCAGAACTACCGACAATCTGGGAGAATGATGAAGCTTGGCTGAATAGTATAATTAAC 1500

P.dactylifera.C.Safawi ATACCAGAACTACCGACAATCTGGGAGAATGATGAAGCTTGGCTGAATAGTATAATTAAC 1500

P.dactylifera.C.Jebeli ATACCAGAACTACCGACAATCTGGGAGAATGATGAAGCTTGGCTGAATAGTATAATTAAC 1500

************************************************************

P.dactylifera.C.Khenezi** GGAGATGGAGAGAGCGATGCAATGCCGGACGTTCGAAATCTGAACACAACGAACTTGCAG 1560

P.dactylifera.C.Ajwah GGAGATGGAAAGAGCGATGCAATGCCGGACGTTCGAAATCTGAACACAACGAACTTGCAG 1560

P.dactylifera.C.Hilwah GGAGATGGAGAGAGCGATGCAATGCCGGACGTTCGAAATCTGAACACAACGAACTTGCAG 1560

P.dactylifera.C.Shalaby GGAGATGGAGAGAGCGATGCAATGCCGGACGTTCGAAATCTGAACACAACGAACTTGCAG 1560

P.dactylifera.C.Anbarah GGAGATGGAAAGAGCGATGCAATGCCGGACGTTCGAAATCTGAACACAACGAACTTGCAG 1560

P.dactylifera.C.Safawi GGAGATGGAAAGAGCGATGCAATGCCGGACGTTCGAAATCTGAACACAACGAACTTGCAG 1560

P.dactylifera.C.Jebeli GGAGATGGAGAGAGCGATGCAATGCCGGACGTTCGAAATCTGAACACAACGAACTTGCAG 1560

********* **************************************************

P.dactylifera.C.Khenezi** AGTGGATTTGGAATAGGGGAGCTTGAGGAAAATAGAGATGGCGCACTGTTTCTGGAAGGA 1620

P.dactylifera.C.Ajwah AGTGGATTTGGAATAGGGGAGCTTGAGGAAAATAGAGATGGCGCACTGTTTCTGGAAGGA 1620

P.dactylifera.C.Hilwah AGTGGATTTGGAATAGGGGAGCTTGAGGAAAATAGAGATGGCGCACTGTTTCTGGAAGGA 1620

P.dactylifera.C.Shalaby AGTGGATTTGGAATAGGGGAGCTTGAGGAAAATAGAGATGGCGCACTGTTTCTGGAAGGA 1620

P.dactylifera.C.Anbarah AGTGGATTTGGAATAGGGGAGCTTGAGGAAAATAGAGATGGCGCACTGTTTCTGGAAGGA 1620

P.dactylifera.C.Safawi AGTGGATTTGGAATAGGGGAGCTTGAGGAAAATAGAGATGGCGCACTGTTTCTGGAAGGA 1620

P.dactylifera.C.Jebeli AGTGGATTTGGAATAGGGGAGCTTGAGGAAAATAGAGATGGCGCACTGTTTCTGGAAGGA 1620

************************************************************

P.dactylifera.C.Khenezi** GTTCTAGGATGGGACGACTTGCTTAGCCACCAT 1653

P.dactylifera.C.Ajwah GTTCTAGGATGGGACGACTTGCTTAGCCACCAT 1653

P.dactylifera.C.Hilwah GTTCTAGGATGGGACGACTTGCTTAGCCACCAT 1653

P.dactylifera.C.Shalaby GTTCTAGGATGGGATGACTTGCTTAGCCACCAT 1653

P.dactylifera.C.Anbarah ATTCTAGGATGGGACGACTTGCTTAGCCACCAT 1653

P.dactylifera.C.Safawi ATTCTAGGATGGGACGACTTGCTTAGCCACCAT 1653

P.dactylifera.C.Jebeli GTTCTAGGATGGGACGACTTGCTTAGCCACCAT 1653

************* ******************

**Figure S1**. Multiple DNA sequence alignment comparing sequence assembled for (VIR^+^) allele of different red date palm cultivars. Nucleotide sequences from red Ajwah, Anbarah, Safawi, Jebeli, Hilwah, Shalaby aligned with Khenezi** (Hazzouri et al., 2015). Asterisks (*) denote single, fully conserved residues.

P.dactylifera.BC4.Male* ATGGGTAATTTGCGTGCCGAAGTCCAACGTCCCGGTGTTCGCAAAGGTGCATGGACTGAA 60

P.dactylifera.C.Khalas*** ATGGGTAATTTGCGTGCCGAAGTCCAACATCCCGGTGTTCGCAAAGGTGCATGGACTGAA 60

P.dactylifera.C.Lulu** ATGGGTAATTTGCGTGCCGAAGTCCAACGTCCCGGTGTTCGCAAAGGTGCATGGACTGAA 60

P.dactylifera.C.Rabiah ATGGGTAATTTGCGTGCCGAAGTCCAACATCCCGGTGTTCGCAAAGGTGCATGGACTGAA 60

P.dactylifera.C.Labana ATGGGTAATTTGCGTGCCGAAGTCCAACGTCCCGGTGTTCGCAAAGGTGCATGGACTGAA 60

P.dactylifera.C.Rothanah ATGGGTAATTTGCGTGCCGAAGTCCAACATCCCGGTGTTCGCAAAGGTGCATGGACTGAA 60

P.dactylifera.C.Baydh ATGGGTAATTTGCGTGCCGAAGTCCAACATCCCGGTGTTCGCAAAGGTGCATGGACTGAA 60

P.dactylifera.C.Khalas ATGGGTAATTTGCGTGCCGAAGTCCAACATCCCGGTGTTCGCAAAGGTGCATGGACTGAA 60

P.dactylifera.C.Sukkary ATGGGTAATTTGCGTGCCGAAGTCCAACGTCCCGGTGTTCGCAAAGGTGCATGGACTGAA 60

P.dactylifera.Rabiah.Male ATGGGTAATTTGCGTGCCGAAGTCCAACATCCCGGTGTTCGCAAAGGTGCATGGACTGAA 60

**************************** *******************************

P.dactylifera.BC4.Male* GAGGAGGACAGGCTTCTAAGGAAGTGCATTGAGATATATGGCGAAGGGAACTGGCGCCAT 120

P.dactylifera.C.Khalas*** GAGGAGGACAGGCTTCTAAGGAAGTGCATTGAGATATATGGCGAAGGGAACTGGCGCCAT 120

P.dactylifera.C.Lulu** GAGGAGGACAGGCTTCTAAGGAAGTGCATTGAGATATATGGCGAAGGGAACTGGCGCCAT 120

P.dactylifera.C.Rabiah GAGGAGGACAGGCTTCTAAGGAAGTGCATTGAGATATATGGCGAAGGGAACTGGCGCCAT 120

P.dactylifera.C.Labana GAGGAGGACAGGCTTCTAAGGAAGTGCATTGAGATATATGGCGAAGGGAACTGGCGCCAT 120

P.dactylifera.C.Rothanah GAGGAGGACAGGCTTCTAAGGAAGTGCATTGAGATATATGGCGAAGGGAACTGGCGCCAT 120

P.dactylifera.C.Baydh GAGGAGGACAGGCTTCTAAGGAAGTGCATTGAGATATATGGCGAAGGGAACTGGCGCCAT 120

P.dactylifera.C.Khalas GAGGAGGACAGGCTTCTAAGGAAGTGCATTGAGATATATGGCGAAGGGAACTGGCGCCAT 120

P.dactylifera.C.Sukkary GAGGAGGACAGGCTTCTAAGGAAGTGCATTGAGATATATGGCGAAGGGAACTGGCGCCAT 120

P.dactylifera.Rabiah.Male GAGGAGGACAGGCTTCTAAGGAAGTGCATTGAGATATATGGCGAAGGGAACTGGCGCCAT 120

************************************************************

P.dactylifera.BC4.Male* GTTCCCGAAAGGGCAGGTAAGCGACTCCTTATAGAGAGTGGAGTCTTTTATCATTCTTAA 180

P.dactylifera.C.Khalas*** GTTCCCGGAAGGGCAGGTAAGCGACTCCTTATAGAGAGTGGGGTCTTTTATCATTCTTGA 180

P.dactylifera.C.Lulu** GTTCCCGAAAGGGCAGGTAAGCGACTCCTTATAGAGAGTGGAGTCTTTTATCATTCTTGA 180

P.dactylifera.C.Rabiah GTTCCCGAAAGGGCAGGTAAGCGACTCCTTATAGAGAGTGGAGTCTTTTATCATTCTTGA 180

P.dactylifera.C.Labana GTTCCCGAAAGGGCAGGTAAGCGACTCCTTATAGAGAGTGGAGTCTTTTATCATTCTTAA 180

P.dactylifera.C.Rothanah GTTCCCGAAAGGGCAGGTAAGCGACTCCTTATAGAGAGTGGGGTCTTTTATCATTCTTGA 180

P.dactylifera.C.Baydh GTTCCCGAAAGGGCAGGTAAGCGACTCCTTATAGAGAGTGGGGTCTTTTATCATTCTTGA 180

P.dactylifera.C.Khalas GTTCCCGAAAGGGCAGGTAAGCGACTCCTTATAGAGAGTGGGGTCTTTTATCATTCTTGA 180

P.dactylifera.C.Sukkary GTTCCCGAAAGGGCAGGTAAGCGACTCCTTATAGAGAGTGGGGTCTTTTATCATTCTTGA 180

P.dactylifera.Rabiah.Male GTTCCCGAAAGGGCAGGTAAGCGACTCCTTATAGAGAGTGGGGTCTTTTATCATTCTTGA 180

******* ********************************* **************** *

P.dactylifera.BC4.Male* GCTTACTCTGCTTTTGTGGGTTTGAAGGCCTCAGAAGGTGCCGAAAGAGCTGCCGATTGC 240

P.dactylifera.C.Khalas*** GCTTACTCTGCTTTTGTGGGTTTGAAGGCCTCAGAAGGTGCCGAAAGAGCTGCCGATTGC 240

P.dactylifera.C.Lulu** GCTTACTCTGNTTTTGTGGGTTTGAAGGCCTCAGAAGGTGCCGAAAGAGCTGCCGATTGC 240

P.dactylifera.C.Rabiah GCTTACTCTGCTTTTGTGGGTTTGAAGGCCTCAGAAGGTGCCGAAAGAGCTGCCGATTGC 240

P.dactylifera.C.Labana GCTTACTCTGCTTTTGTGGGTTTGAAGGCCTCAGAAGGTGCCGAAAGAGCTGCCGATTGC 240

P.dactylifera.C.Rothanah GCTTACTCTGCTTTTGTGGGTTTGAAGGCCTCAGAAGGTGCCGAAAGAGCTGCCGATTGC 240

P.dactylifera.C.Baydh GCTTACTCTGCTTTTGTGGGTTTGAAGGCCTCAGAAGGTGCCGAAAGAGCTGCCGATTGC 240

P.dactylifera.C.Khalas GCTTACTCTGCTTTTGTGGGTTTGAAGGCCTCAGAAGGTGCCGAAAGAGCTGCCGATTGC 240

P.dactylifera.C.Sukkary GCTTACTCTGCTTTTGTGGGTTTGAAGGCCTCAGAAGGTGCCGAAAGAGCTGCCGATTGC 240

P.dactylifera.Rabiah.Male GCTTACTCTGCTTTTGTGGGTTTGAAGGCCTCAGAAGGTGCCGAAAGAGCTGCCGATTGC 240

********** *************************************************

P.dactylifera.BC4.Male* GTTGGTTGAACTATCTCTGCCCCAGGATCAACAGGGAGAAATTTTCTGAGGAAGAAACAG 300

P.dactylifera.C.Khalas*** GTTGGTTGAACTATCTCTGCCCCAGGATCAACAGGGAGAAATTTTCTGAGGAAGAAACAG 300

P.dactylifera.C.Lulu** GTTGGTTGAACTATCTCTGNCCCAGGATCAACAGGGAGAAATTTTCTGAGGAAGAAACAG 300

P.dactylifera.C.Rabiah GTTGGTTGAACTATCTCTGCCCCAGGATCAACAGGGAGAAATTTTCTGAGGAAGAAACAG 300

P.dactylifera.C.Labana GTTGGTTGAACTATCTCTGCCCCAGGATCAACAGGGAGAAATTTTCTGAGGAAGAAACAG 300

P.dactylifera.C.Rothanah GTTGGTTGAACTATCTCTGCCCCAGGATCAACAGGGAGAAATTTTCTGAGGAAGAAACAG 300

P.dactylifera.C.Baydh GTTGGTTGAACTATCTCTGCCCCAGGATCAACAGGGAGAAATTTTCTGAGGAAGAAACAG 300

P.dactylifera.C.Khalas GTTGGTTGAACTATCTCTGCCCCAGGATCAACAGGGAGAAATTTTCTGAGGAAGAAACAG 300

P.dactylifera.C.Sukkary GTTGGTTGAACTATCTCTGCCCCAGGATCAACAGGGAGAAATTTTCTGAGGAAGAAACAG 300

P.dactylifera.Rabiah.Male GTTGGTTGAACTATCTCTGCCCCAGGATCAACAGGGAGAAATTTTCTGAGGAAGAAACAG 300

******************* ****************************************

P.dactylifera.BC4.Male* ATCTTATCATTAGGCTTCATAAGATCTTGGGCAACAGGTAACAACAGACTATAAAGATTG 360

P.dactylifera.C.Khalas*** ATCTTATCATTAGGCTTCATAAGATCTTGGGTAACAGGTAACAACAGACTATAAAGATTG 360

P.dactylifera.C.Lulu** ATCTTATCATTAGGCTTCATAAGATCTTGGGCAACAGGTAACAACAGACTATAAAGATTG 360

P.dactylifera.C.Rabiah ATCTTATCATTAGGCTTCATAAGATCTTGGGCAACAGGTAACAACAGACTATAAAGATTG 360

P.dactylifera.C.Labana ATCTTATCATTAGGCTTCATAAGATCTTGGGCAACAGGTAACAACAGACTATAAAGATTG 360

P.dactylifera.C.Rothanah ATCTTATCATTAGGCTTCATAAGATCTTGGGCAACAGGTAACAACAGACTATAAAGATTG 360

P.dactylifera.C.Baydh ATCTTATCATTAGGCTTCATAAGATCTTGGGCAACAGGTAACAACAGACTATAAAGATTG 360

P.dactylifera.C.Khalas ATCTTATCATTAGGCTTCATAAGATCTTGGGTAACAGGTAACAACAGACTATAAAGATTG 360

P.dactylifera.C.Sukkary ATCTTATCATTAGGCTTCATAAGATCTTGGGCAACAGGTAACAACAGACTATAAAGATTG 360

P.dactylifera.Rabiah.Male ATCTTATCATTAGGCTTCATAAGATCTTGGGCAACAGGTAACAACAGACTATAAAGATTG 360

******************************* ****************************

P.dactylifera.BC4.Male* CCTTGGTTACAGTGACCATGTATTTGTTTTCTATCAAGCGATTCCAAAAGTTTCGCTTCC 420

P.dactylifera.C.Khalas*** CCTTGGTTACAGTGACCATGTATTTGTTTTCTATCAAGCGATTCCAAAAGTTTCGCTTCC 420

P.dactylifera.C.Lulu** CCTTGGTTACAGTGACCATGTATTTGTTTTCTATCAAGCGATTCCAAAAGTTTCGCTTCC 420

P.dactylifera.C.Rabiah CCTTGGTTACAGTGACCATGTATTTGTTTTCTATCAAGCGATTCCAAAAGTTTCGCTTCC 420

P.dactylifera.C.Labana CCTTGGTTACAGTGACCATATATTTGTTTTCTATCAAGCGATTCCAAAAGTTTCGCTTCC 420

P.dactylifera.C.Rothanah CCTTGGTTACAGTGACCATGTATTTGTTTTCTATCAAGCGATTCCAAAAGTTTCGCTTCC 420

P.dactylifera.C.Baydh CCTTGGTTACAGTGACCATGTATTTGTTTTCTATCAAGCGATTCCAAAAGTTTCGCTTCC 420

P.dactylifera.C.Khalas CCTTGGTTACAGTGACCATGTATTTGTTTTCTATCAAGCGATTCCAAAAGTTTCGCTTCC 420

P.dactylifera.C.Sukkary CCTTGGTTACAGTGACCATGTATTTGTTTTCTATCAAGCGATTCCAAAAGTTTCGCTTCC 420

P.dactylifera.Rabiah.Male CCTTGGTTACAGTGACCATGTATTTGTTTTCTATCAAGCGATTCCAAAAGTTTCGCTTCC 420

******************* ****************************************

P.dactylifera.BC4.Male* CTTACCTTCATATTCCTTACCTGAGATAGTATAAGAAAATAACTACGAGATCATTCATTG 480

P.dactylifera.C.Khalas*** CTTACCTTCATATTCCTTACCTGAGATAGTATAAGAAAATAACTACGAGATCATTCATTG 480

P.dactylifera.C.Lulu** CTTNCCTTCATATTCCTTACCTGAGATAGTATAANAAAATAACTACGAGATCATTCATTG 480

P.dactylifera.C.Rabiah CTTTCCTTCATATTCCTTACCTGAGATAGTATAAGAAAATAACTACGAGATCATTCATTA 480

P.dactylifera.C.Labana CTTTCCTTCATATTCCTTACCTGAGATAGTATAAGAAAATAACTACGAGATCATTCATTA 480

P.dactylifera.C.Rothanah CTTACCTTCATATTCCTTACCTGAGATAGTATAAGAAAATAACTACGAGATCATTCATTG 480

P.dactylifera.C.Baydh CTTACCTTCATATTCCTTACCTGAGATAGTATAAGAAAATAACTACGAGATCATTCATTG 480

P.dactylifera.C.Khalas CTTACCTTCATATTCCTTACCTGAGATAGTATAAGAAAATAACTACGAGATCATTCATTG 480

P.dactylifera.C.Sukkary CTTACCTTCATATTCCTTACCTGAGATAGTATAAAAAAATAACTACGAGATCATTCATTA 480

P.dactylifera.Rabiah.Male CTTACCTTCATATTCCTTACCTGAGATAGTATAAGAAAATAACTACGAGATCATTCATTG 480

*** ****************************** ************************

P.dactylifera.BC4.Male* TATACATGTTTCTTTTAGTGCAGCTGTCAGCTCTCGTGGTTGGCACCTCTCTCCTAAGGA 540

P.dactylifera.C.Khalas*** TATACATGTTTCTTTTAGTGCAGCTGTCAGCTCTCGTGGTTGGCACCTCTCTCCTAAGGA 540

P.dactylifera.C.Lulu** TATACATGTTTCTTTTAGTGCAGCTGTCAGCTCTCGTGGTTGGCACCTCTCTCCTAAGGA 540

P.dactylifera.C.Rabiah TATACATGTTTCTTTTAGTGCAGCTGTCAGCTCTCGTGGTTGGCACCTCTCTCCTAAGGA 540

P.dactylifera.C.Labana TATACATGTTTCTTTTAGTGCAGCTGTCAGCTCTCGTGGTTGGCACCTCTCTCCTAAGGA 540

P.dactylifera.C.Rothanah TATACATGTTTCTTTTAGTGCAGCTGTCAGCTCTCGTGGTTGGCACCTCTCTCCTAAGGA 540

P.dactylifera.C.Khalas TATACATGTTTCTTTTAGTGCAGCTGTCAGCTCTCGTGGTTGGCACCTCTCTCCTAAGGA 540

P.dactylifera.C.Baydh TATACATGTTTCTTTTAGTGCAGCTGTCAGCTCTCGTGGTTGGCACCTCTCTCCTAAGGA 540

P.dactylifera.C.Sukkary TATACATGTTTCTTTTAGTGCAGCTGTCAGCTCTCGTGGTTGGCACCTCTCTCCTAAGGA 540

P.dactylifera.Rabiah.Male TATACATGTTTCTTTTAGTGCAGCTGTCAGCTCTCGTGGTTGGCACCTCTCTCCTAAGGA 540

************************************************************

P.dactylifera.BC4.Male* GTTGTCTTTATGGCATATAAAGTTCAAATCGTAAATAAAGTTAGTTTTCAAAAAAATACG 600

P.dactylifera.C.Khalas*** GTTGTCTTTATGGCATATAAAGTTCAAATCGTAAATAAAGTTAGTTTTCAAAAAAATACG 600

P.dactylifera.C.Lulu** GTTGTCTCTATGGCATATAAAGTTCAAATCNTAAATAAAGTTAGTTTTCAGAAAAATACG 600

P.dactylifera.C.Rabiah GTTGTCTTTATGGCATATAAAGTTCAAATCGTAAATAAAGTTAGTTTTCAAAAAAATACG 600

P.dactylifera.C.Labana GTTGTCTTTATGGCATATAAAGTTCAAATCGTAAATAAAGTTAGTTTTCAAAAAAATACA 600

P.dactylifera.C.Rothanah GTTGTCTTTATGGCATATAAAGTTCAAATCGTAAATAAAGTTAGTTTTCAAAAAAATACG 600

P.dactylifera.C.Baydh GTTGTCTTTATGGCATATAAAGTTCAAATCGTAAATAAAGTTAGTTTTCAAAAAAATACG 600

P.dactylifera.C.Khalas GTTGTCTTTATGGCATATAAAGTTCAAATCGTAAATAAAGTTAGTTTTCAAAAAAATACG 600

P.dactylifera.C.Sukkary GTTGTCTTTATGGCATATAAAGTTCAAATCGTAAATAAAGTTAGTTTTCAAAAAAATACG 600

P.dactylifera.Rabiah.Male GTTGTCTTTATGGCATATAAAGTTCAAATCGTAAATAAAGTTAGTTTTCAAAAAAATACG 600

******* ********************** ******************* ********

P.dactylifera.BC4.Male* AAAAGATATGATATGTTTAAAACATTATGCATTTTTAAAATTCTCGCATCTCTTCAGTTT 660

P.dactylifera.C.Khalas*** AAAAGATATGATATGTTTAAAACATTATGCATTTTTAAAATTCTCGGATCTCTTCAGTTT 660

P.dactylifera.C.Lulu** AAAAGATATGATATGTTTAAAACATTATGCATTTTTAAAATTCTCGCATCTCTTCAGTTT 660

P.dactylifera.C.Rabiah AAAAGATATGGTATGTTTAAAACATTATGTATTTTTAAAATTCTCGGATCTCTTCAGTTT 660

P.dactylifera.C.Labana AAAAGATATGGTATGTTTAAAACATTATGTATTTTTAAAATTCTCGCATCTCTTCAGTTT 660

P.dactylifera.C.Rothanah AAAAGATATGATATGTTTAAAACATTATGCATTTTTAAAATTCTCGCATCTCTTCAGTTT 660

P.dactylifera.C.Baydh AAAAGATATGATATGTTTAAAACATTATGCATTTTTAAAATTCTCGCATCTCTTCAGTTT 660

P.dactylifera.C.Khalas AAAAGATATGATATGTTTAAAACATTATGCATTTTTAAAATTCTCGGATCTCTTCAGTTT 660

P.dactylifera.C.Sukkary AAAAGATATGGTATGTTTAAAACATTATGTATTTTTAAAATTCTCGCATCTCTTCAGTTT 660

P.dactylifera.Rabiah.Male AAAAGATATGATATGTTTAAAACATTATGCATTTTTAAAATTCTCGCATCTCTTCAGTTT 660

********** ****************** **************** *************

P.dactylifera.BC4.Male* TCCAAATAAAGTCTATAAAAACACTTCTCTAGGATTAATTCAAAAAAACAATCTGATCAT 720

P.dactylifera.C.Khalas*** TCCAAATAAAGTCTATAAAAACACTTCTCTAGGATTAATTCAAAAAAACAATCTGATCAT 720

P.dactylifera.C.Lulu** TCCAAATAGAGCCTATAAATACACTTCTCTANGATTAATTCAAAAAAACAATCTGATCAT 720

P.dactylifera.C.Rabiah TCCAAATAAAGTCTATAAAAACACTTCTCTAGGATTAATTCAAAAAAACAATCTGATCAT 720

P.dactylifera.C.Labana TCCAAATAGAGTCTATAAATATACTTCTCTAGGATTAATTCAAAAAAACAATCTGATCAT 720

P.dactylifera.C.Rothanah TCCAAATAAAGTCTATAAAAACACTTCTCTAGGATTAATTCAAAAAAACAATCTGATCAT 720

P.dactylifera.C.Baydh TCCAAATAAAGTCTATAAAAACACTTCTCTAGGATTAATTCAAAAAAACAATCTGATCAT 720

P.dactylifera.C.Khalas TCCAAATAAAGTCTATAAAAACACTTCTCTAGGATTAATTCAAAAAAACAATCTGATCAT 720

P.dactylifera.C.Sukkary TCCAAATAAAGTCTATAAATATACTTCTCTAGGATTAATTCAAAAAAACAATCTGATCAT 720

P.dactylifera.Rabiah.Male TCCAAATAAAGTCTATAAAAACACTTCTCTAGGATTAATTCAAAAAAACAATCTGATCAT 720

******** ** ******* * ********* ****************************

P.dactylifera.BC4.Male* TTTCTTTTTTACTGGTACTCTGTCTTCTGAGGATTAATTCAATGGTACTCTTTCTCACTG 780

P.dactylifera.C.Khalas*** TTTCT-TTTTACTGGTACTCTGTCTTCTGAGGATTAATTCAATGGTACTCTTTCTCACTG 779

P.dactylifera.C.Lulu** TTTCANTGTTACTGGTACTCTGTCTTCTGAGGATTAATTCACTGGTACTCTTTCTCACTG 780

P.dactylifera.C.Rabiah TTTCTTTTTTACTGGTACTCTGTCTTCTGAGGATTAATTCAATGGTACTCTTTCTCACTG 780

P.dactylifera.C.Labana TTTCTTTTTTACTGGTACTCTGTCTTCTGAGGATTAATTCACTGGTACTCTTTCTCACTG 780

P.dactylifera.C.Rothanah TTTCTTTTTTACTGGTACTCTGTCTTCTGAGGATTAATTCAATGGTACTCTTTCTCACTG 780

P.dactylifera.C.Baydh TTTCTTTTTTACTGGTACTCTGTCTTCTGAGGATTAATTCAATGGTACTCTTTCTCACTG 780

P.dactylifera.C.Khalas TTTCTTTTTTACTGGTACTCTGTCTTCTGAGGATTAATTCAATGGTACTCTTTCTCACTG 780

P.dactylifera.C.Sukkary TTTCTTTTTTACTGGTACTCTGTCTTCTGAGGATTAATTCAATGGTACTCTTTCTCACTG 780

P.dactylifera.Rabiah.Male TTTCTTTTTTACTGGTACTCTGTCTTCTGAGGATTAATTCAATGGTACTCTTTCTCACTG 780

**** * ********************************* ******************

P.dactylifera.BC4.Male* ATAATTTGAGCAAACGAAACCTTGCTCCAAAATTCAGACAAAGGGGCTGCTCATAAAATT 840

P.dactylifera.C.Khalas*** ATAATTTGAGCAAACGAAACCTTGCTCCAAAATTCAGACAAAGGGGCTGCTCATAAAATT 839

P.dactylifera.C.Lulu** CTAATTTGAGCAAACGAAACCTTGCTCCAAAATTCAGACAAAGGGGCTGCTCATAAAATT 840

P.dactylifera.C.Rabiah ATAATTTGAGCAAACGAAACCTTGCTCCAAAATTCAGACAAAGGGGCTGCTCATAAAATT 840

P.dactylifera.C.Labana CTAATTTGAGCAAACGAGACCTTGCTCCAAGATTCAGACAAAGGGGCTGCTCATAAAATT 840

P.dactylifera.C.Rothanah ATAATTTGAGCAAACGAAACCTTGCTCCAAAATTCAGACAAAGGGGCTGCTCATAAAATT 840

P.dactylifera.C.Baydh ATAATTTGAGCAAACGAAACCTTGCTCCAAAATTCAGACAAAGGGGCTGCTCATAAAATT 840

P.dactylifera.C.Khalas ATAATTTGAGCAAACGAAACCTTGCTCCAAAATTCAGACAAAGGGGCTGCTCATAAAATT 840

P.dactylifera.C.Sukkary CTAATTTGAGCAAACGAAACCTTGCTCCAAAATTCAGACAAAGGGGCTGCTCATAAAATT 840

P.dactylifera.Rabiah.Male ATAATTTGAGCAAACGAAACCTTGCTCCAAAATTCAGACAAAGGGGCTGCTCATAAAATT 840

**************** ************ *****************************

P.dactylifera.BC4.Male* CCTTAAGGAAAACTGCAACAAATCACCTTCTAAAAAAAGGAACCAATGCATTTAAACCTG 900

P.dactylifera.C.Khalas*** CCTTAAGGAAAACTGCAACAAATCACCTTCTAAAAAAAGGAACCAATGCATTTAAACCTG 899

P.dactylifera.C.Lulu** CCTTAAGGAAAACTGCAACAAATCACCTTCTAAAAAAAGGAACCAATGCATTTAAACCTG 900

P.dactylifera.C.Rabiah CCTTAAGGAAAACTGCAACAAATCACCTTCTAAAAAAAGGAACCAATGCATTTAAACCTG 900

P.dactylifera.C.Labana CCTTTAGGAAAACTGCAACACATCACCTTCTAATAAAAGGAACCAATGCATTTAAACCTG 900

P.dactylifera.C.Rothanah CCTTAAGGAAAACTGCAACAAATCACCTTCTAAAAAAAGGAACCAATGCATTTAAACCTG 900

P.dactylifera.C.Baydh CCTTAAGGAAAACTGCAACAAATCACCTTCTAAAAAAAGGAACCAATGCATTTAAACCTG 900

P.dactylifera.C.Khalas CCTTAAGGAAAACTGCAACAAATCACCTTCTAAAAAAAGGAACCAATGCATTTAAACCTG 900

P.dactylifera.C.Sukkary CCTTAAGGAAAACTGCAACAAATCACCTTCTAAAAAAAGGAACCAATGCATTTAAACCTG 900

P.dactylifera.Rabiah.Male CCTTAAGGAAAACTGCAACAAATCACCTTCTAAAAAAAGGAACCAATGCATTTAAACCTG 900

**** *************** ************ **************************

P.dactylifera.BC4.Male* TACAGAGGAAATAGTTTTTTGGGCCTAAAGAAAATAAGTGATAACATCAACACATCTTCA 960

P.dactylifera.C.Khalas*** TACAGAGGAAGTAGTCTTTTGGGCCTAAAGAAAATAAGTGATAACATCAACACATCTTCA 959

P.dactylifera.C.Lulu** TACAGAGGAAGTAGTCTTTTGGGCCTAAAGAAAATAAGTGATAACATCAACACATCTTCA 960

P.dactylifera.C.Rabiah TACAGAGGAAGTAGTCTTTTGGGCCTAAAGAAAATAAGTGATAACATCAACACATCTTCA 960

P.dactylifera.C.Labana TCCAGAGGAAGTTGTCTTTTGGGCCTAAAGAAAATAAGTGATAATATCAACACATCTTCA 960

P.dactylifera.C.Rothanah TACAGAGGAAGTAGTCTTTTGGGCCTAAAGAAAATAAGTGATAACATCAACACATCTTCA 960

P.dactylifera.C.Baydh TACAGAGGAAGTAGTCTTTTGGGCCTAAAGAAAATAAGTGATAACATCAACACATCTTCA 960

P.dactylifera.C.Khalas TACAGAGGAAGTAGTCTTTTGGGCCTAAAGAAAATAAGTGATAACATCAACACATCTTCA 960

P.dactylifera.C.Sukkary TACAGAGGAAGTAGTCTTTTGGGCCTAAAGAAAATAAGTGATAACATCAACACATCTTCA 960

P.dactylifera.Rabiah.Male TACAGAGGAAGTAGTCTTTTGGGCCTAAAGAAAATAAGTGATAACATCAACACATCTTCA 960

* ******** * ** **************************** ***************

P.dactylifera.BC4.Male* GTCTATTTTTTCCGTACAATTTTTTTGGAATCTCCATCCTCATTTTTTCATTCAATTGTA 1020

P.dactylifera.C.Khalas*** GTCTATTTTTTCCGTACAATTTTTTTGGAATCTCCATCCCCATTTTTTCATTCAATTGTA 1019

P.dactylifera.C.Lulu** GTCTATTTTTTCCGTACAATTTTTTTGGAATCTCCATCCNCATTTTTTCATTCAATTGTA 1020

P.dactylifera.C.Rabiah GTCTATTTTTTCCGTACAATTTTTTTGGAATCTCCATCCCCATTTTTTCATTCAATTGTA 1020

P.dactylifera.C.Labana GTCTATTTTTCCCGTACAATTTTTTTGGAATCTCCACCCCATTTTTTTCATACAATTGTA 1020

P.dactylifera.C.Rothanah GTCTATTTTTTCCGTACAATTTTTTTGGAATCTCCATCCCCATTTTTTCATTCAATTGTA 1020

P.dactylifera.C.Baydh GTCTATTTTTTCCGTACAATTTTTTTGGAATCTCCATCCCCATTTTTTCATTCAATTGTA 1020

P.dactylifera.C.Khalas GTCTATTTTTTCCGTACAATTTTTTTGGAATCTCCATCCCCATTTTTTCATTCAATTGTA 1020

P.dactylifera.C.Sukkary GTCTATTTTTTCCGTACAATTTTTTTGGAATCTCCATCCCCATTTTTTCATTCAATTGTA 1020

P.dactylifera.Rabiah.Male GTCTATTTTTTCCGTACAATTTTTTTGGAATCTCCATCCCCATTTTTTCATTCAATTGTA 1020

********** ************************* ** ********* ********

P.dactylifera.BC4.Male* TCTTTGATTTCATTCTTGAGATCTTTCCACTTACTCCAAATGCAATGGGGTATGCAACCA 1080

P.dactylifera.C.Khalas*** TCTTTGATTTCATTCTTGAGATCTTTCCACTTACTCCAAATGCAATGTGGTATGCAACCA 1079

P.dactylifera.C.Lulu** TCTTTGATTTCATTCTTGAGATCTTTCCACTTACTCCAAATGCAATGNGGTATGCAACCA 1080

P.dactylifera.C.Rabiah TCTTTGATTTCATTCTTGAGATCTTTCCACTTACTCCAAATGCAATGTGGTATGCAACCA 1080

P.dactylifera.C.Labana TCTTTGATTTCATTCTTGAGATCTTTCCACTTACTCCAAATGCAATGGGGTATGCAACCA 1080

P.dactylifera.C.Rothanah TCTTTGATTTCATTCTTGAGATCTTTCCACTTACTCCAAATGCAATGTGGTATGCAACCA 1080

P.dactylifera.C.Baydh TCTTTGATTTCATTCTTGAGATCTTTCCACTTACTCCAAATGCAATGTGGTATGCAACCA 1080

P.dactylifera.C.Khalas TCTTTGATTTCATTCTTGAGATCTTTCCACTTACTCCAAATGCAATGTGGTATGCAACCA 1080

P.dactylifera.C.Sukkary TCTTTGATTTCATTCTTGAGATCTTTCCACTTACTCCAAATGCAATGTGGTATGCAACCA 1080

P.dactylifera.Rabiah.Male TCTTTGATTTCATTCTTGAGATCTTTCCACTTACTCCAAATGCAATGTGGTATGCAACCA 1080

*********************************************** ************

P.dactylifera.BC4.Male* CTGGGCTCTAGCAATATTTGCTGTGTAGAGATGTTCGTGTGCCGTACTTTTCCTGAATCT 1140

P.dactylifera.C.Khalas*** CTGGGCTCTAGCAATATTTGCTGTGTAGAGATGTTCGTGTGCCGTACTTTTCCTGAATCT 1139

P.dactylifera.C.Lulu** CTGGGCTCTAGCAATATTTGCTGTGTAGAGATGTTCGTGTACCGTACTTTTCCTGAATCT 1140

P.dactylifera.C.Rabiah CTGGGCTCTAGCAATATTTGCTGTGTAGAGATGTTCGTGTACCGTACTTTTCCTGAATCT 1140

P.dactylifera.C.Labana CCGGGCTCTAGCAATATTTGCTGTGTAGAGATGTTCGTGTACCGTACTTTTCTTGAATCT 1140

P.dactylifera.C.Rothanah CTGGGCTCTAGCAATATTTGCTGTGTAGAGATGTTCGTGTACCGTACTTTTCCTGAATCT 1140

P.dactylifera.C.Baydh CTGGGCTCTAGCAATATTTGCTGTGTAGAGATGTTCGTGTACCGTACTTTTCCTGAATCT 1140

P.dactylifera.C.Khalas CTGGGCTCTAGCAATATTTGCTGTGTAGAGATGTTCGTGTACCGTACTTTTCCTGAATCT 1140

P.dactylifera.C.Sukkary CTGGGCTCTAGCAATATTTGCTGTGTAGAGATGTTCGTGTACCGTACTTTTCCTGAATCT 1140

P.dactylifera.Rabiah.Male CTGGGCTCTAGCAATATTTGCTGTGTAGAGATGTTCGTGTACCGTACTTTTCCTGAATCT 1140

* ************************************** *********** *******

P.dactylifera.BC4.Male* CTCGTCCTGCAAGGGACCCTCTCGTCACAGTTGCAA----ACAATTCAACTAATGAGCCC 1196

P.dactylifera.C.Khalas*** CTCGTCCTGCAAGGGACCCTCTCGTCACAGTTGCAA----ACAATTCAACTAATGAGCCC 1195

P.dactylifera.C.Lulu** CTCGTCCTGCAAGGGACCCTCTCGTCACAGTTGCAA----ACAATTCAACTAATGAGCTC 1196

P.dactylifera.C.Rabiah CTCGTCCTGCAAGGGACCCTCTCGTCACAGTTGCAA----ACAATTCAACTAATGAGCTC 1196

P.dactylifera.C.Labana CTCGTCCTGCAAGGGACCCTCTCGTCACAGTTGAACACAAACAATTCAACTAATGAGCTC 1200

P.dactylifera.C.Rothanah CTCGTCCTGCAAGGGACCCTCTCGTCACAGTTGCAA----ACAATTCAACTAATGAGCTC 1196

P.dactylifera.C.Baydh CTCGTCCTGCAAGGGACCCTCTCGTCACAGTTGCAA----ACAATTCAACTAATGAGCTC 1196

P.dactylifera.C.Khalas CTCGTCCTGCAAGGGACCCTCTCGTCACAGTTGCAA----ACAATTCAACTAATGAGCTC 1196

P.dactylifera.C.Sukkary CTCGTCCTGCAAGGGACCCTCTCGTCACAGTTGCAA----ACAATTCAACTAATGAGCTC 1196

P.dactylifera.Rabiah.Male CTCGTCCTGCAAGGGACCCTCTCGTCACAGTTGCAA----ACAATTCAACTAATGAGCTC 1196

********************************* * ****************** *

P.dactylifera.BC4.Male* TGAATTCGGTCAATCAGGTGGTCAACAATTGCAGGTAGGCTTCCAGGCAGGACAGCAAAT 1256

P.dactylifera.C.Khalas*** TGAATTCGGTCAATCAGGTGGTCAACAATTGCAGGTAGGCTTCCAGGCAGGACAGCAAAT 1255

P.dactylifera.C.Lulu** TGAATTCGGTCAATCAGGTGGTCAACAATTGCAGGTAGGCTTCCAGGCAGGACAGCAAAT 1256

P.dactylifera.C.Rabiah TGAATTCGGTCAATCAGGTGGTCAACAATTGCAGGTAGGCTTCCAGGCAGGACAGCAAAT 1256

P.dactylifera.C.Labana TGAATTCGGTCAATCAGGTGGTCAACAATTGCAGGTAGGCTTCCAGGCAGGACAGCAAAT 1260

P.dactylifera.C.Rothanah TGAATTCGGTCAATCAGGTGGTCAACAATTGCAGGTAGGCTTCCAGGCAGGACAGCAAAT 1256

P.dactylifera.C.Baydh TGAATTCGGTCAATCAGGTGGTCAACAATTGCAGGTAGGCTTCCAGGCAGGACAGCAAAT 1256

P.dactylifera.C.Khalas TGAATTCGGTCAATCAGGTGGTCAACAATTGCAGGTAGGCTTCCAGGCAGGACAGCAAAT 1256

P.dactylifera.C.Sukkary TGAATTCGGTCAATCAGGTGGTCAACAATTGCAGGTAGGCTTCCAGGCAGGACAGCAAAT 1256

P.dactylifera.Rabiah.Male TGAATTCGGTCAATCAGGTGGTCAACAATTGCAGGTAGGCTTCCAGGCAGGACAGCAAAT 1256

************************************************************

P.dactylifera.BC4.Male* GACATCAAGAACTACTGGAATTCTCAGCTGAGCAAGAAAGTAGAAGTTGAAAACAAGAAG 1316

P.dactylifera.C.Khalas*** GACATCAAGAACTACTGGAATTCTCAGCTGAGCAAGAAAGTAGAAGTTGAAAACAAGAAG 1315

P.dactylifera.C.Lulu** GACATCAAGAACTACTGGAATTCTCAGCTGAGCAAGAAAGTAGAAGTTGAAAACAAGAAG 1316

P.dactylifera.C.Rabiah GACATCAAGAACTACTGGAATTCTCAGCTGAGCAAGAAAGTAGAAGTTGAAAACAAGAAG 1316

P.dactylifera.C.Labana GACATCAAGAACTACTGGAATTCTCAGCTGAGCAAGAAAGTAGAAGTTGAAAACAAGAAG 1320

P.dactylifera.C.Rothanah GACATCAAGAACTACTGGAATTCTCAGCTGAGCAAGAAAGTAGAAGTTGAAAACAAGAAG 1316

P.dactylifera.C.Baydh GACATCAAGAACTACTGGAATTCTCAGCTGAGCAAGAAAGTAGAAGTTGAAAACAAGAAG 1316

P.dactylifera.C.Khalas GACATCAAGAACTACTGGAATTCTCAGCTGAGCAAGAAAGTAGAAGTTGAAAACAAGAAG 1316

P.dactylifera.C.Sukkary GACATCAAGAACTACTGGAATTCTCAGCTGAGCAAGAAAGTAGAAGTTGAAAACAAGAAG 1316

P.dactylifera.Rabiah.Male GACATCAAGAACTACTGGAATTCTCAGCTGAGCAAGAAAGTAGAAGTTGAAAACAAGAAG 1316

************************************************************

P.dactylifera.BC4.Male* GCGGAGCCCAGTGCTGATGCCAAAGTAATTAAGCCACAGCCATGGAGAGTACCGTTGCAG 1376

P.dactylifera.C.Khalas*** GCGGAGCCCAGTGCTGATGCCAAAGTAATTAAGCCACAGCCATGGAGAGTACCGTTGCAG 1375

P.dactylifera.C.Lulu** GCGGAGCCCAGTGCTGATGCCAAAGTAATTAAGCCACGGCCATGGAGAGTACCGTTGCAG 1376

P.dactylifera.C.Rabiah GCGGAGCCCAGTGCTGATGCCAAAGTAATTAAGCCACGGCCATGGAGAGTACCGTTGCAG 1376

P.dactylifera.C.Labana GCGGAGCCCAGTGCTGATGCCAAAGTAATTAAGCCACGGCCATGGAGAGTACCGTTGCAG 1380

P.dactylifera.C.Rothanah GCGGAGCCCAGTGCTGATGCCAAAGTAATTAAGCCACGGCCATGGAGAGTACCGTTGCAG 1376

P.dactylifera.C.Baydh GCGGAGCCCAGTGCTGATGCCAAAGTAATTAAGCCACGGCCATGGAGAGTACCGTTGCAG 1376

P.dactylifera.C.Khalas GCGGAGCCCAGTGCTGATGCCAAAGTAATTAAGCCACGGCCATGGAGAGTACCGTTGCAG 1376

P.dactylifera.C.Sukkary GCGGAGCCCAGTGCTGATGCCAAAGTAATTAAGCCACGGCCATGGAGAGTACCGTTGCAG 1376

P.dactylifera.Rabiah.Male GCGGAGCCCAGTGCTGATGCCAAAGTAATTAAGCCACGGCCATGGAGAGTACCGTTGCAG 1376

************************************* **********************

P.dactylifera.BC4.Male* TGGATTTGGTCAGGAGATCAACAATCATGTGGAAGTCAGCACCAGCAAGAAAAGTTTGGC 1436

P.dactylifera.C.Khalas*** TGGATTTGGTCAGGAGATCAACAATCATGTGGAAGTCAGCACCAGCAAGAAAAGTTTGGC 1435

P.dactylifera.C.Lulu** TGGATTTGGTCAGGAGATCAACAATCATGTGGAAGTCAGCACCAGCAAGAAAAGTTTGGC 1436

P.dactylifera.C.Rabiah TGGATTTGGTCAGGAGATCAACAATCATGTGGAAGTCAGCACCAGCAAGAAAAGTTTGGC 1436

P.dactylifera.C.Labana TGGATTTGGTCAGGAGATCAACAATCATGTGGAAGTCAGCACCAGCAAGAAAAGTTTGGC 1440

P.dactylifera.C.Rothanah TGGATTTGGTCAGGAGATCAACAATCATGTGGAAGTCAGCACCAGCAAGAAAAGTTTGGC 1436

P.dactylifera.C.Baydh TGGATTTGGTCAGGAGATCAACAATCATGTGGAAGTCAGCACCAGCAAGAAAAGTTTGGC 1436

P.dactylifera.C.Khalas TGGATTTGGTCAGGAGATCAACAATCATGTGGAAGTCAGCACCAGCAAGAAAAGTTTGGC 1436

P.dactylifera.C.Sukkary TGGATTTGGTCAGGAGATCAACAATCATGTGGAAGTCAGCACCAGCAAGAAAAGTTTGGC 1436

P.dactylifera.Rabiah.Male TGGATTTGGTCAGGAGATCAACAATCATGTGGAAGTCAGCACCAGCAAGAAAAGTTTGGC 1436

************************************************************

P.dactylifera.BC4.Male* ATACCAGTGTTAGAATAATTTCAAAATATGGGCTTACACTAATTGCGTATTTTTATTTTA 1496

P.dactylifera.C.Khalas*** ATACCAGTGTTAGAATAATTTCAAAATATGGGCTTACACTAATTGCGTATTTTTATTTTA 1495

P.dactylifera.C.Lulu** ATACCAGTGTTAGAATAATTTCAAAATATGGGCTTACACTGATTGCGTATTTTTATTTTA 1496

P.dactylifera.C.Rabiah ATACCAGTGTTAGAATAATTTCAAAATATGGGCTTACACTGATTGCGTATTTTTATTTTA 1496

P.dactylifera.C.Labana ATACCAGTGTTAGAATAATTTCAAAATATGGGCTTACACTGATTGCGTATTTTTATTTTA 1500

P.dactylifera.C.Rothanah ATACCAGTGTTAGAATAATTTCAAAATATGGGCTTACACTGATTGCGTATTTTTATTTTA 1496

P.dactylifera.C.Baydh ATACCAGTGTTAGAATAATTTCAAAATATGGGCTTACACTGATTGCGTATTTTTATTTTA 1496

P.dactylifera.C.Khalas ATACCAGTGTTAGAATAATTTCAAAATATGGGCTTACACTGATTGCGTATTTTTATTTTA 1496

P.dactylifera.C.Sukkary ATACCAGTGTTAGAATAATTTCAAAATATGGGCTTACACTGATTGCGTATTTTTATTTTA 1496

P.dactylifera.Rabiah.Male ATACCAGTGTTAGAATAATTTCAAAATATGGGCTTACACTGATTGCGTATTTTTATTTTA 1496

**************************************** *******************

P.dactylifera.BC4.Male* TAAAACGGGTTAGACGTATGGCTCAGAATGGTACTATGGTTAAGCCCAATATGATGTGTG 1556

P.dactylifera.C.Khalas*** TAAAACGGGTTAGACGTATGGCTCAGAATGGTACTATGGTTAAGCCCAATATGATGTGTG 1555

P.dactylifera.C.Lulu** TAAAACGGATTAGACGTATGGCTCAGAATGGTACTATGGTTAAGCCCAATATGATGTGTG 1556

P.dactylifera.C.Rabiah TAAAACGGGTTAGACGTATGGCTCAGAATGGTACTATGGTTAAGCCCAATATGATGTGTG 1556

P.dactylifera.C.Labana TAAAACGGGTTAGACGTATGGCTCAGAATGGTACTATGGTTAAGCCCAATATGATGTGTG 1560

P.dactylifera.C.Rothanah TAAAACGGGTTAGACGTATGGCTCAGAATGGTACTATGGTTAAGCCCAATATGATGTGTG 1556

P.dactylifera.C.Baydh TAAAACGGGTTAGACGTATGGCTCAGAATGGTACTATGGTTAAGCCCAATATGATGTGTG 1556

P.dactylifera.C.Khalas TAAAACGGGTTAGACGTATGGCTCAGAATGGTACTATGGTTAAGCCCAATATGATGTGTG 1556

P.dactylifera.C.Sukkary TAAAACGGATTAGACGTATGGCTCAGAATGGTACTATGGTTAAGCCCAATATGATGTGTG 1556

P.dactylifera.Rabiah.Male TAAAACGGGTTAGACGTATGGCTCAGAATGGTACTATGGTTAAGCCCAATATGATGTGTG 1556

******** ***************************************************

P.dactylifera.BC4.Male* ATTATTTTATTTTATGGGCTTTAGTGCATCTTGGATGTATAGGATTGAGTCCTATTTAAT 1616

P.dactylifera.C.Khalas*** ATTATTTTATTTTATGGGCTTTAGTGCATCTTGGATGTATAGGATTGAGTCCTATTTAAT 1615

P.dactylifera.C.Lulu** ATTATTTTATTTTATAGGCTTTGGTGCACCTTGGATGTATAGGATTGAGTCCTATTTAAT 1616

P.dactylifera.C.Rabiah ATTATTTTATTTTATGGGCTTTAGTGCATCTTGGATGTATAGGATTGAGTCCTATTTAAT 1616

P.dactylifera.C.Labana ATTATTTTATTTTATGGGCTTTAGTGCATCTTGGATGTATAGGATTGAGTCCTATTTAAT 1620

P.dactylifera.C.Rothanah ATTATTTTATTTTATGGGCTTTAGTGCATCTTGGATGTATAGGATTGAGTCCTATTTAAT 1616

P.dactylifera.C.Baydh ATTATTTTATTTTATGGGCTTTAGTGCATCTTGGATGTATAGGATTGAGTCCTATTTAAT 1616

P.dactylifera.C.Khalas ATTATTTTATTTTATGGGCTTTAGTGCATCTTGGATGTATAGGATTGAGTCCTATTTAAT 1616

P.dactylifera.C.Sukkary ATTATTTTATTTTATAGGCTTTGGTGCATCTTGGATGTATAGGATTGAGTCCTATTTAAT 1616

P.dactylifera.Rabiah.Male ATTATTTTATTTTATGGGCTTTAGTGCATCTTGGATGTATAGGATTGAGTCCTATTTAAT 1616

*************** ****** ***** *******************************

P.dactylifera.BC4.Male* GTGGTAATTTTAGTATGTAGGCCAGGAAACCCAATTAGGATTTTGGAGCCTTTAATGGGA 1676

P.dactylifera.C.Khalas*** GTGGTAATTTTAGTATGTAGGCCAGGAAACCCAATTAGGATTTTGGAGCCTTTAATGGGA 1675

P.dactylifera.C.Lulu** GTGGTAATTTTAGTATGTAGGCCACGAAACCCAATTAGGGTTTTGGAGCCTTTAATGGGA 1676

P.dactylifera.C.Rabiah GTGGTAATTTTAGTATGTAGGCCAGGAAACCCAATTAGGATTTTGGAGCCTTTAATGGGA 1676

P.dactylifera.C.Labana GTGGTAATTTTAGTATGTAGGCCAGGAAACCCAATTAGGATTTTGGAGCCTTTAATGGGA 1680

P.dactylifera.C.Rothanah GTGGTAATTTTAGTATGTAGGCCAGGAAACCCAATTAGGATTTTGGAGCCTTTAATGGGA 1676

P.dactylifera.C.Baydh GTGGTAATTTTAGTATGTAGGCCAGGAAACCCAATTAGGATTTTGGAGCCTTTAATGGGA 1676

P.dactylifera.C.Khalas GTGGTAATTTTAGTATGTAGGCCAGGAAACCCAATTAGGATTTTGGAGCCTTTAATGGGA 1676

P.dactylifera.C.Sukkary GTGGTAATTTTAGTATGTAGGCCAGGAAACCCAATTAGGATTTTGGAGCCTTTAATGGGA 1676

P.dactylifera.Rabiah.Male GTGGTAATTTTAGTATGTAGGCCAGGAAACCCAATTAGGATTTTGGAGCCTTTAATGGGA 1676

************************ ************** ********************

P.dactylifera.BC4.Male* AAGCTCTCTTATATTTCCTATATAATGGCTAGGTCCCCCTCTCCTCTCCATATGGTTGAT 1736

P.dactylifera.C.Khalas*** AAGCTCTCTTATATTTCCTATATAAAGGCTAGGTCCCCCTCTCCTCTCCATATGGTTGAT 1735

P.dactylifera.C.Lulu** AGGCTCTCTTATATTTTCTATATAAAGGCTAGGTCCCCCTCTCCTCTCCATATGGTTGAT 1736

P.dactylifera.C.Rabiah AAGCTCTCTTATATTTCCTATATAAAGGCTAGGTCCCCCTCTCCTCTCCATATGGTTGAT 1736

P.dactylifera.C.Labana AAGCTCTCTTATATTTCCTATATAAAGGCTAGGTCCCCCTCTCCTCTCCATATGGTTGAT 1740

P.dactylifera.C.Rothanah AAGCTCTCTTATATTTCCTATATAAAGGCTAGGTCCCCCTCTCCTCTCCATATGGTTGAT 1736

P.dactylifera.C.Baydh AAGCTCTCTTATATTTCCTATATAAAGGCTAGGTCCCCCTCTCCTCTCCATATGGTTGAT 1736

P.dactylifera.C.Khalas AAGCTCTCTTATATTTCCTATATAAAGGCTAGGTCCCCCTCTCCTCTCCATATGGTTGAT 1736

P.dactylifera.C.Sukkary AAGCTCTCTTATATTTCCTATATAAAGGCTAGGTCCCCCTCTCCTCTCCATATGGTTGAT 1736

P.dactylifera.Rabiah.Male AAGCTCTCTTATATTTCCTATATAAAGGCTAGGTCCCCCTCTCCTCTCCATATGGTTGAT 1736

* ************** ******** **********************************

P.dactylifera.BC4.Male* AGCTTGCCCCATTTACGGCTATCTTTTATGAGGAGCAAGGAAGGGAAGATCCAGCCGCTA 1796

P.dactylifera.C.Khalas*** AGCTTGCCCCATTTACGGCTATCTTTTATGAGGAGCAAGGAAGGGAAGATCCAGCCGCTA 1795

P.dactylifera.C.Lulu** AGCTTGCCCCATTGACGGTTATCTTTTGTGAAGAGCAAGGAAGGGAAGATCCAGCCGCTA 1796

P.dactylifera.C.Rabiah AGCTTGCCCCATTTACGGCTATCTTTTATGAGGAGCAAGGAAGGGAAGATCCAGCCGCTA 1796

P.dactylifera.C.Labana AGCTTGCCCCATTTACGGCTATCTTTTATGAGGAGCAAGGAAGGGAAGATCCAGCCGCTA 1800

P.dactylifera.C.Rothanah AGCTTGCCCCATTTACGGCTATCTTTTATGAGGAGCAAGGAAGGGAAGATCCAGCCGCTA 1796

P.dactylifera.C.Baydh AGCTTGCCCCATTTACGGCTATCTTTTATGAGGAGCAAGGAAGGGAAGATCCAGCCGCTA 1796

P.dactylifera.C.Khalas AGCTTGCCCCATTTACGGCTATCTTTTATGAGGAGCAAGGAAGGGAAGATCCAGCCGCTA 1796

P.dactylifera.C.Sukkary AGCTTGCCCCATTTACGGCTATCTTTTGTGAGGAGCAAGGAAGGGAAGATCCAGCCGCTA 1796

P.dactylifera.Rabiah.Male AGCTTGCCCCATTTACGGCTATCTTTTATGAGGAGCAAGGAAGGGAAGATCCAGCCGCTA 1796

************* **** ******** *** ****************************

P.dactylifera.BC4.Male* TTATTCCAGGTGATTGAAGAACGTATTCAATATGTCTTCTGCAGGTATATCTCTCAAACT 1856

P.dactylifera.C.Khalas*** TTATTCCAGGTGATTGAAGAACGTATTCAATATGTCTTCTGCAGGTATATCTCTCAAACT 1855

P.dactylifera.C.Lulu** TTATTCCAGATGATTGAAGAACGTATTCAATATGTCTTCCG------------------- 1837

P.dactylifera.C.Rabiah TTATTCCAGGTGATTGAAGAACGTATTCAATATGTCTTCCGCAGGTATATCTCTCAAATT 1856

P.dactylifera.C.Labana TTATTCCAGGTGATTGAAGAACGTATTCAATATGTCTTC--------------------- 1839

P.dactylifera.C.Rothanah TTATTCCAGGTGATTGAAGAACGAGTTCA---------C--------------------- 1826

P.dactylifera.C.Baydh TTATTCCAGGTGATTGAAGAACGTATTCAATATGTCTTCCGCAGGTATATCTCTCAAATT 1856

P.dactylifera.C.Khalas TTATTCCAGGTGATTGAAGAACGTATTCAATATGTCTTC--------------------- 1835

P.dactylifera.C.Sukkary TTATTCCAGGTGATTGAAGAACGGATTCAAT----------------------------- 1827

P.dactylifera.Rabiah.Male TTATTCCAGGTGATTGAAGAACGTATT--------------------------------- 1823

********* ************* **

P.dactylifera.BC4.Male* TGAATACTGTATTATTGTGATTCTGAATCTTAGCATGTTATCATTTTTATAATTAACATT 1916

P.dactylifera.C.Khalas*** TGAATACTGTATTATTGTGATTCTGAATCTTAGCATGTTATCATTTTTATAATTAACATT 1915

P.dactylifera.C.Lulu** ------------------------------------------------------------ 1837

P.dactylifera.C.Rabiah TGAATACTTTATTATTGTGATTCTGAATCTTAGCATGTTATCATTTTTATAATTAACAAC 1916

P.dactylifera.C.Labana ------------------------------------------------------------ 1839

P.dactylifera.C.Rothanah ------------------------------------------------------------ 1826

P.dactylifera.C.Baydh TGAATACTTTATTATTGTGATTCTGAATCTTAGCATGTTATCATTTTTATAATTAACAAC 1916

P.dactylifera.C.Khalas ------------------------------------------------------------ 1835

P.dactylifera.C.Sukkary ------------------------------------------------------------ 1827

P.dactylifera.Rabiah.Male ------------------------------------------------------------ 1823

P.dactylifera.BC4.Male* TGGTTAAGGAATTTTATTTCTGGACTGAAAGTTGTTGATTCCATCTCGAGACCAATTACC 1976

P.dactylifera.C.Khalas*** TGG--------------------------------------------------------- 1918

P.dactylifera.C.Lulu** CAGAACTACCGGCAATCTGGGAGAATGATGAAGCTTGGCTGAATAGTATAATTAACGGAG 1897

P.dactylifera.C.Rabiah CAGAACTACCGACAATCTGGGAGAATGATGAAGCTTGGCTGAATAGTATAATTAACGGAG 1976

P.dactylifera.C.Labana CAGAACTACCGACAATCTGGGAGAATGATGAAGCTTGGCTGAATAGTATAATTAACGGAG 1899

P.dactylifera.C.Rothanah CAGAACTACCGACAATCTGGGAGAATGATGAAGCTTGGCTGAATAGTATAATTAACGGAG 1886

P.dactylifera.C.Baydh CAGAACTACCGGCAATCTGGGAGAATGATGAAGCTTGGCTGAATAGTATAATTAACGGAG 1976

P.dactylifera.C.Khalas ------------------------------------------------------------ 1835

P.dactylifera.C.Sukkary ------------------------------------------------------------ 1827

P.dactylifera.Rabiah.Male ------------------------------------------------------------ 1823

P.dactylifera.BC4.Male* ATCTACTGTGATAATAGTGCAGCCGTGTTCTTTTCAAAGAATAACAAGAGTTCTGGTGGC 2036

P.dactylifera.C.Khalas*** ------------------------------------------------------------ 1918

P.dactylifera.C.Lulu** ATGGAGAGAGAAGTGCAATGCC-------------------------------------- 1919

P.dactylifera.C.Rabiah ATGGAGAGAGCGATGCAATGCCGGACGTTCGAAATCTGAACACAACGAACTTGCAGAGTG 2036

P.dactylifera.C.Labana ATGGAGAGAGCGATGCAATGCCGGACGTTCGAAATCTGAACACAACGAACTTGCAGAGTG 1959

P.dactylifera.C.Rothanah ATGGAGAGAGCGATGCAATGCCGGACGTTCGAAATCTGAACACAACGAACTTGCAGAGTG 1946

P.dactylifera.C.Baydh ATGGAGAGAGAAGTGC---------------AATGCCGGACACAACGAACGTGCAGAGTG 2021

P.dactylifera.C.Khalas ------------------------------------------------------------ 1835

P.dactylifera.C.Sukkary ------------------------------------------------------------ 1827

P.dactylifera.Rabiah.Male ------------------------------------------------=----------- 1823

P.dactylifera.BC4.Male* TCTAAGCATATAGACATCAAGTACTTGGTAGTCAGAGTTAAAGTTAAAGAAGGGCAGACA 2096

P.dactylifera.C.Khalas*** ------------------------------------------------------------ 1918

P.dactylifera.C.Lulu** ------------------------------------------------------------ 1919

P.dactylifera.C.Rabiah GATTTGGAATAGGGGAGCTTGAGGAAAATAGAGATGGCGCACTGTTTCTGGAAGGAGTTC 2096

P.dactylifera.C.Labana GATTTGGAATAGGGGAGCTTGAGGAAAATAGAGATGGCGCACTGTTTCTGGAAGGAGTTC 2019

P.dactylifera.C.Rothanah GATTTGGAATAGGGGAGCTTGAGGAAAATAGAGATGGCGCACTGTTTCTGGAAGGAGTTC 2006

P.dactylifera.C.Baydh GATTTGGAATAGGAGAGCTTGAGGAAAATAGAGATGGCGCACTGTTTCTGGAAGGAGTTC 2081

P.dactylifera.C.Khalas ------------------------------------------------------------ 1835

P.dactylifera.C.Sukkary ------------------------------------------------------------ 1827

P.dactylifera.Rabiah.Male ------------------------------------------------------------ 1823

P.dactylifera.BC4.Male* AAACACTACAAGAAAATACATATTCAGCGACTAAGATTCTAGTCGCCGAATAACCATATT 2156

P.dactylifera.C.Khalas*** ------------------------------------------------------------ 1918

P.dactylifera.C.Lulu** ------------------------------------------------------------ 1919

P.dactylifera.C.Rabiah TAGGATGGGACGACTTGCTTAGCCACCAT------------------------------- 2125

P.dactylifera.C.Labana TAGGATGGGATGACTTGCTTAGCCACCAT------------------------------- 2048

P.dactylifera.C.Rothanah TAGGATGGGACGACTTGC------------------------------------------ 2024

P.dactylifera.C.Baydh TAGGAT------------------------------------------------------ 2087

P.dactylifera.C.Khalas ------------------------------------------------------------ 1835

P.dactylifera.C.Sukkary ------------------------------------------------------------ 1827

P.dactylifera.Rabiah.Male ------------------------------------------------------------ 1823

P.dactylifera.BC4.Male* TGGTCGCCGAAATCTTTTCGCGACTAGCATACCCTTCGTCGCCGAACCTTAGTCAGTGAA 2216

P.dactylifera.C.Khalas*** ------------------------------------------------------------ 1918

P.dactylifera.C.Lulu** ------------------------------------------------------------ 1919

P.dactylifera.C.Rabiah ------------------------------------------------------------ 2125

P.dactylifera.C.Labana ------------------------------------------------------------ 2048

P.dactylifera.C.Rothanah ------------------------------------------------------------ 2024

P.dactylifera.C.Baydh ------------------------------------------------------------ 2087

P.dactylifera.C.Khalas ------------------------------------------------------------ 1835

P.dactylifera.C.Sukkary ------------------------------------------------------------ 1827

P.dactylifera.Rabiah.Male ------------------------------------------------------------ 1823

P.dactylifera.BC4.Male* AGATTTTGGCGACCAATATTGTGTGGTTACCTAAGGTACACCAACAACGACCAACGTTTG 2276

P.dactylifera.C.Khalas*** ------------------------------------------------------------ 1918

P.dactylifera.C.Lulu** ------------------------------------------------------------ 1919

P.dactylifera.C.Rabiah ------------------------------------------------------------ 2125

P.dactylifera.C.Labana ------------------------------------------------------------ 2048

P.dactylifera.C.Rothanah ------------------------------------------------------------ 2024

P.dactylifera.C.Baydh ------------------------------------------------------------ 2087

P.dactylifera.C.Khalas ------------------------------------------------------------ 1835

P.dactylifera.C.Sukkary ------------------------------------------------------------ 1827

P.dactylifera.Rabiah.Male ------------------------------------------------------------ 1823

P.dactylifera.BC4.Male* GTTGCCGAAGAAAATTATCAGCGACCAAATCTGTTGTCGCTAATTTCAACGCCGACCAAA 2336

P.dactylifera.C.Khalas*** ------------------------------------------------------------ 1918

P.dactylifera.C.Lulu** ------------------------------------------------------------ 1919

P.dactylifera.C.Rabiah ------------------------------------------------------------ 2125

P.dactylifera.C.Labana ------------------------------------------------------------ 2048

P.dactylifera.C.Rothanah ------------------------------------------------------------ 2024

P.dactylifera.C.Baydh ------------------------------------------------------------ 2087

P.dactylifera.C.Khalas ------------------------------------------------------------ 1835

P.dactylifera.C.Sukkary ------------------------------------------------------------ 1827

P.dactylifera.Rabiah.Male ------------------------------------------------------------ 1823

P.dactylifera.BC4.Male* TAGTTGGTCGTGGAAGGTTTGTCTTCCGCTACCAAAACAATGGTCACTGAATGTCTATCT 2396

P.dactylifera.C.Khalas*** ------------------------------------------------------------ 1918

P.dactylifera.C.Lulu** ------------------------------------------------------------ 1919

P.dactylifera.C.Rabiah ------------------------------------------------------------ 2125

P.dactylifera.C.Labana ------------------------------------------------------------ 2048

P.dactylifera.C.Rothanah ------------------------------------------------------------ 2024

P.dactylifera.C.Baydh ------------------------------------------------------------ 2087

P.dactylifera.C.Khalas ------------------------------------------------------------ 1835

P.dactylifera.C.Sukkary ------------------------------------------------------------ 1827

P.dactylifera.Rabiah.Male ------------------------------------------------------------ 1823

P.dactylifera.BC4.Male* TCCCCTACCAATAGTTTGGTCGCAGAATGTATTGGAATGCATTGGCAACAAACATTTTTT 2456

P.dactylifera.C.Khalas*** ------------------------------------------------------------ 1918

P.dactylifera.C.Lulu** ------------------------------------------------------------ 1919

P.dactylifera.C.Rabiah ------------------------------------------------------------ 2125

P.dactylifera.C.Labana ------------------------------------------------------------ 2048

P.dactylifera.C.Rothanah ------------------------------------------------------------ 2024

P.dactylifera.C.Baydh ------------------------------------------------------------ 2087

P.dactylifera.C.Khalas ------------------------------------------------------------ 1835

P.dactylifera.C.Sukkary ------------------------------------------------------------ 1827

P.dactylifera.Rabiah.Male ------------------------------------------------------------ 1823

P.dactylifera.BC4.Male* AGTGGCAGAAAGTATTAACCTGTATTTAATTAACAATTCAAATTATAAGCTTAATATTTA 2516

P.dactylifera.C.Khalas*** ------------------------------------------------------------ 1918

P.dactylifera.C.Lulu** ------------------------------------------------------------ 1919

P.dactylifera.C.Rabiah ------------------------------------------------------------ 2125

P.dactylifera.C.Labana ------------------------------------------------------------ 2048

P.dactylifera.C.Rothanah ------------------------------------------------------------ 2024

P.dactylifera.C.Baydh ------------------------------------------------------------ 2087

P.dactylifera.C.Khalas ------------------------------------------------------------ 1835

P.dactylifera.C.Sukkary ------------------------------------------------------------ 1827

P.dactylifera.Rabiah.Male ------------------------------------------------------------ 1823

P.dactylifera.BC4.Male* TTTTTGGGCTCGTTCCAAATCCTGTACAACCAACACAGCCTAAGCCTATCCAATATCCAT 2576

P.dactylifera.C.Khalas*** ------------------------------------------------------------ 1918

P.dactylifera.C.Lulu** ------------------------------------------------------------ 1919

P.dactylifera.C.Rabiah ------------------------------------------------------------ 2125

P.dactylifera.C.Labana ------------------------------------------------------------ 2048

P.dactylifera.C.Rothanah ------------------------------------------------------------ 2024

P.dactylifera.C.Baydh ------------------------------------------------------------ 2087

P.dactylifera.C.Khalas ------------------------------------------------------------ 1835

P.dactylifera.C.Sukkary ------------------------------------------------------------ 1827

P.dactylifera.Rabiah.Male ------------------------------------------------------------ 1823

P.dactylifera.BC4.Male* ATTGCACGATACATTTGCTCATCCAAATAGAAATATACACAATGTTGGAGAATCCATAAT 2636

P.dactylifera.C.Khalas*** ------------------------------------------------------------ 1918

P.dactylifera.C.Lulu** ------------------------------------------------------------ 1919

P.dactylifera.C.Rabiah ------------------------------------------------------------ 2125

P.dactylifera.C.Labana ------------------------------------------------------------ 2048

P.dactylifera.C.Rothanah ------------------------------------------------------------ 2024

P.dactylifera.C.Baydh ------------------------------------------------------------ 2087

P.dactylifera.C.Khalas ------------------------------------------------------------ 1835

P.dactylifera.C.Sukkary ------------------------------------------------------------ 1827

P.dactylifera.Rabiah.Male ------------------------------------------------------------ 1823

P.dactylifera.BC4.Male* CATTCATTATAAATATCATCATCTAAAAGTCTAAAGTCAAGCATACCTATATATTAACCA 2696

P.dactylifera.C.Khalas*** ------------------------------------------------------------ 1918

P.dactylifera.C.Lulu** ------------------------------------------------------------ 1919

P.dactylifera.C.Rabiah ------------------------------------------------------------ 2125

P.dactylifera.C.Labana ------------------------------------------------------------ 2048

P.dactylifera.C.Rothanah ------------------------------------------------------------ 2024

P.dactylifera.C.Baydh ------------------------------------------------------------ 2087

P.dactylifera.C.Khalas ------------------------------------------------------------ 1835

P.dactylifera.C.Sukkary ------------------------------------------------------------ 1827

P.dactylifera.Rabiah.Male ------------------------------------------------------------ 1823

P.dactylifera.BC4.Male* TACATCAAAATGTTTCATCCATACCATATGTTAAGGTGATAATCATCACCAAACATATCA 2756

P.dactylifera.C.Khalas*** ------------------------------------------------------------ 1918

P.dactylifera.C.Lulu** ------------------------------------------------------------ 1919

P.dactylifera.C.Rabiah ------------------------------------------------------------ 2125

P.dactylifera.C.Labana ------------------------------------------------------------ 2048

P.dactylifera.C.Rothanah ------------------------------------------------------------ 2024

P.dactylifera.C.Baydh ------------------------------------------------------------ 2087

P.dactylifera.C.Khalas ------------------------------------------------------------ 1835

P.dactylifera.C.Sukkary ------------------------------------------------------------ 1827

P.dactylifera.Rabiah.Male ------------------------------------------------------------ 1823

P.dactylifera.BC4.Male* AAATGTAAATTAAAAATCCCAAAATATCTGATCTTGCTATTCATCCAAACAACCATCATG 2816

P.dactylifera.C.Khalas*** ------------------------------------------------------------ 1918

P.dactylifera.C.Lulu** ------------------------------------------------------------ 1919

P.dactylifera.C.Rabiah ------------------------------------------------------------ 2125

P.dactylifera.C.Labana ------------------------------------------------------------ 2048

P.dactylifera.C.Rothanah ------------------------------------------------------------ 2024

P.dactylifera.C.Baydh ------------------------------------------------------------ 2087

P.dactylifera.C.Khalas ------------------------------------------------------------ 1835

P.dactylifera.C.Sukkary ------------------------------------------------------------ 1827

P.dactylifera.Rabiah.Male ------------------------------------------------------------ 1823

P.dactylifera.BC4.Male* TAGACAAAAATATTCTTATACCAAAAACTGGATTCTGCTTGTAGACAACTCCACCATGAC 2876

P.dactylifera.C.Khalas*** ------------------------------------------------------------ 1918

P.dactylifera.C.Lulu** ------------------------------------------------------------ 1919

P.dactylifera.C.Rabiah ------------------------------------------------------------ 2125

P.dactylifera.C.Labana ------------------------------------------------------------ 2048

P.dactylifera.C.Rothanah ------------------------------------------------------------ 2024

P.dactylifera.C.Baydh ------------------------------------------------------------ 2087

P.dactylifera.C.Khalas ------------------------------------------------------------ 1835

P.dactylifera.C.Sukkary ------------------------------------------------------------ 1827

P.dactylifera.Rabiah.Male ------------------------------------------------------------ 1823

P.dactylifera.BC4.Male* CATCTGAACAAAAGCTATCCATGTACTAGAAACTAGATTTGACATGCTCATAACTCCACC 2936

P.dactylifera.C.Khalas*** ------------------------------------------------------------ 1918

P.dactylifera.C.Lulu** ------------------------------------------------------------ 1919

P.dactylifera.C.Rabiah ------------------------------------------------------------ 2125

P.dactylifera.C.Labana ------------------------------------------------------------ 2048

P.dactylifera.C.Rothanah ------------------------------------------------------------ 2024

P.dactylifera.C.Baydh ------------------------------------------------------------ 2087

P.dactylifera.C.Khalas ------------------------------------------------------------ 1835

P.dactylifera.C.Sukkary ------------------------------------------------------------ 1827

P.dactylifera.Rabiah.Male ------------------------------------------------------------ 1823

P.dactylifera.BC4.Male* ATGTATTTGAGAAAGCAAAAAAGAATACACAATCAATAACCACATATGAAAAATAATAAA 2996

P.dactylifera.C.Khalas*** ------------------------------------------------------------ 1918

P.dactylifera.C.Lulu** ------------------------------------------------------------ 1919

P.dactylifera.C.Rabiah ------------------------------------------------------------ 2125

P.dactylifera.C.Labana ------------------------------------------------------------ 2048

P.dactylifera.C.Rothanah ------------------------------------------------------------ 2024

P.dactylifera.C.Baydh ------------------------------------------------------------ 2087

P.dactylifera.C.Khalas ------------------------------------------------------------ 1835

P.dactylifera.C.Sukkary ------------------------------------------------------------ 1827

P.dactylifera.Rabiah.Male ------------------------------------------------------------ 1823

P.dactylifera.BC4.Male* CTAAAATTCAAGTATAAGTTCACAATGACATTTCGCTTAATTTGGTCCAAATCTCTAAAT 3056

P.dactylifera.C.Khalas*** ------------------------------------------------------------ 1918

P.dactylifera.C.Lulu** ------------------------------------------------------------ 1919

P.dactylifera.C.Rabiah ------------------------------------------------------------ 2125

P.dactylifera.C.Labana ------------------------------------------------------------ 2048

P.dactylifera.C.Rothanah ------------------------------------------------------------ 2024

P.dactylifera.C.Baydh ------------------------------------------------------------ 2087

P.dactylifera.C.Khalas ------------------------------------------------------------ 1835

P.dactylifera.C.Sukkary ------------------------------------------------------------ 1827

P.dactylifera.Rabiah.Male ------------------------------------------------------------ 1823

P.dactylifera.BC4.Male* AACCTACATAATATAAATGAGAATACATACCAACGAGTATTTGTACCACTACTTGAATAC 3116

P.dactylifera.C.Khalas*** ------------------------------------------------------------ 1918

P.dactylifera.C.Lulu** ------------------------------------------------------------ 1919

P.dactylifera.C.Rabiah ------------------------------------------------------------ 2125

P.dactylifera.C.Labana ------------------------------------------------------------ 2048

P.dactylifera.C.Rothanah ------------------------------------------------------------ 2024

P.dactylifera.C.Baydh ------------------------------------------------------------ 2087

P.dactylifera.C.Khalas ------------------------------------------------------------ 1835

P.dactylifera.C.Sukkary ------------------------------------------------------------ 1827

P.dactylifera.Rabiah.Male ------------------------------------------------------------ 1823

P.dactylifera.BC4.Male* ATAAACCCCTCCTCACAAAACCACTCTCATACGCACACAAATAGCACCATATCTATTCCA 3176

P.dactylifera.C.Khalas*** ------------------------------------------------------------ 1918

P.dactylifera.C.Lulu** ------------------------------------------------------------ 1919

P.dactylifera.C.Rabiah ------------------------------------------------------------ 2125

P.dactylifera.C.Labana ------------------------------------------------------------ 2048

P.dactylifera.C.Rothanah ------------------------------------------------------------ 2024

P.dactylifera.C.Baydh ------------------------------------------------------------ 2087

P.dactylifera.C.Khalas ------------------------------------------------------------ 1835

P.dactylifera.C.Sukkary ------------------------------------------------------------ 1827

P.dactylifera.Rabiah.Male ------------------------------------------------------------ 1823

P.dactylifera.BC4.Male* CAAGTATTAACCTTGATAAGAAAATTGTGCAAGAAACAGCCAGGTGATGATCAAAGTTAA 3236

P.dactylifera.C.Khalas*** ------------------------------------------------------------ 1918

P.dactylifera.C.Lulu** ------------------------------------------------------------ 1919

P.dactylifera.C.Rabiah ------------------------------------------------------------ 2125

P.dactylifera.C.Labana ------------------------------------------------------------ 2048

P.dactylifera.C.Rothanah ------------------------------------------------------------ 2024

P.dactylifera.C.Baydh ------------------------------------------------------------ 2087

P.dactylifera.C.Khalas ------------------------------------------------------------ 1835

P.dactylifera.C.Sukkary ------------------------------------------------------------ 1827

P.dactylifera.Rabiah.Male ------------------------------------------------------------ 1823

P.dactylifera.BC4.Male* GCTGCAGATTTAAGAAATAAACTTGAAAGGAAAAGAACATGATATCAGATTATAAAGGAA 3296

P.dactylifera.C.Khalas*** ------------------------------------------------------------ 1918

P.dactylifera.C.Lulu** ------------------------------------------------------------ 1919

P.dactylifera.C.Rabiah ------------------------------------------------------------ 2125

P.dactylifera.C.Labana ------------------------------------------------------------ 2048

P.dactylifera.C.Rothanah ------------------------------------------------------------ 2024

P.dactylifera.C.Baydh ------------------------------------------------------------ 2087

P.dactylifera.C.Khalas ------------------------------------------------------------ 1835

P.dactylifera.C.Sukkary ------------------------------------------------------------ 1827

P.dactylifera.Rabiah.Male ------------------------------------------------------------ 1823

P.dactylifera.BC4.Male* GCGAGCATAAATGATTAAAACTGATCTGCATCGTAGGCACCAAAATGCACATTTCTTAAG 3356

P.dactylifera.C.Khalas*** ------------------------------------------------------------ 1918

P.dactylifera.C.Lulu** ------------------------------------------------------------ 1919

P.dactylifera.C.Rabiah ------------------------------------------------------------ 2125

P.dactylifera.C.Labana ------------------------------------------------------------ 2048

P.dactylifera.C.Rothanah ------------------------------------------------------------ 2024

P.dactylifera.C.Baydh ------------------------------------------------------------ 2087

P.dactylifera.C.Khalas ------------------------------------------------------------ 1835

P.dactylifera.C.Sukkary ------------------------------------------------------------ 1827

P.dactylifera.Rabiah.Male ------------------------------------------------------------ 1823

P.dactylifera.BC4.Male* TTAATAAATGGAAACAATTGTCCATAACAAATGGAAGCTATTGTAAGTAACAGAACTGGT 3416

P.dactylifera.C.Khalas*** ------------------------------------------------------------ 1918

P.dactylifera.C.Lulu** ------------------------------------------------------------ 1919

P.dactylifera.C.Rabiah ------------------------------------------------------------ 2125

P.dactylifera.C.Labana ------------------------------------------------------------ 2048

P.dactylifera.C.Rothanah ------------------------------------------------------------ 2024

P.dactylifera.C.Baydh ------------------------------------------------------------ 2087

P.dactylifera.C.Khalas ------------------------------------------------------------ 1835

P.dactylifera.C.Sukkary ------------------------------------------------------------ 1827

P.dactylifera.Rabiah.Male ------------------------------------------------------------ 1823

P.dactylifera.BC4.Male* ATCAAGCTGTTTCCAGAGCAAATTGACTCGTTTTCCCTTGACAGGTGAGTGAAACAGACA 3476

P.dactylifera.C.Khalas*** ------------------------------------------------------------ 1918

P.dactylifera.C.Lulu** ------------------------------------------------------------ 1919

P.dactylifera.C.Rabiah ------------------------------------------------------------ 2125

P.dactylifera.C.Labana ------------------------------------------------------------ 2048

P.dactylifera.C.Rothanah ------------------------------------------------------------ 2024

P.dactylifera.C.Baydh ------------------------------------------------------------ 2087

P.dactylifera.C.Khalas ------------------------------------------------------------ 1835

P.dactylifera.C.Sukkary ------------------------------------------------------------ 1827

P.dactylifera.Rabiah.Male ------------------------------------------------------------ 1823

P.dactylifera.BC4.Male* TTTTATTGCTAATCTGCCCCTCACAAGTCTGGATATAATAAAATATCCTGATTTAAAAAG 3536

P.dactylifera.C.Khalas*** ------------------------------------------------------------ 1918

P.dactylifera.C.Lulu** ------------------------------------------------------------ 1919

P.dactylifera.C.Rabiah ------------------------------------------------------------ 2125

P.dactylifera.C.Labana ------------------------------------------------------------ 2048

P.dactylifera.C.Rothanah ------------------------------------------------------------ 2024

P.dactylifera.C.Baydh ------------------------------------------------------------ 2087

P.dactylifera.C.Khalas ------------------------------------------------------------ 1835

P.dactylifera.C.Sukkary ------------------------------------------------------------ 1827

P.dactylifera.Rabiah.Male ------------------------------------------------------------ 1823

P.dactylifera.BC4.Male* AATGGAGTTCAATTGAAACATCAATGAGTGATGATGTCCTAAGCAGGCGTGA 3588

P.dactylifera.C.Khalas*** ---------------------------------------------------- 1918

P.dactylifera.C.Lulu** ---------------------------------------------------- 1919

P.dactylifera.C.Rabiah ---------------------------------------------------- 2125

P.dactylifera.C.Labana ---------------------------------------------------- 2048

P.dactylifera.C.Rothanah ---------------------------------------------------- 2024

P.dactylifera.C.Baydh ---------------------------------------------------- 2087

P.dactylifera.C.Khalas ---------------------------------------------------- 1835

P.dactylifera.C.Sukkary ---------------------------------------------------- 1827

P.dactylifera.Rabiah.Male ---------------------------------------------------- 1823

**Figure S2.** Multiple DNA sequence alignment comparing sequence assembled for (VIR^IM^) allele of different yellow date palm cultivars. Nucleotide sequences from yellow cultivars Rothanah, Rabiah, Labana, Baydh, Sukkary, Khalas and Male originated from Rabiah aligned with BC4 Male* (Hazzouri et al., 2019), Lulu**(Hazzouri et al., 2015) and Khalas*** (Al-Mssallem et al., 2013). Asterisks (*) denote single, fully conserved residues.

P.dactylifera.BC4.Male* AAGTTTGGCATACCAGTGTTAGAATAATTTCAAAATATGGGCTTACACTAATTGCGTATT 60

P.dactylifera.C.Khalas*** AAGTTTGGCATACCAGTGTTAGAATAATTTCAAAATATGGGCTTACACTAATTGCGTATT 60

P.dactylifera.C.Rabiah AAGTTTGGCATACCAGTGTTAGAATAATTTCAAAATATGGGCTTACACTGATTGCGTATT 60

P.dactylifera.C.Baydh AAGTTTGGCATACCAGTGTTAGAATAATTTCAAAATATGGGCTTACACTGATTGCGTATT 60

P.dactylifera.C.Lulu** AAGTTTGGCATACCAGTGTTAGAATAATTTCAAAATATGGGCTTACACTGATTGCGTATT 60

P.dactylifera.C.Labana AAGTTTGGCATACCAGTGTTAGAATAATTTCAAAATATGGGCTTACACTGATTGCGTATT 60

P.dactylifera.C.Rothanah AAGTTTGGCATACCAGTGTTAGAATAATTTCAAAATATGGGCTTACACTGATTGCGTATT 60

P.dactylifera.C.Khalas AAGTTTGGCATACCAGTGTTAGAATAATTTCAAAATATGGGCTTACACTGATTGCGTATT 60

P.dactylifera.C.Sukkary AAGTTTGGCATACCAGTGTTAGAATAATTTCAAAATATGGGCTTACACTGATTGCGTATT 60

P.dactylifera.Rabiah.Male AAGTTTGGCATACCAGTGTTAGAATAATTTCAAAATATGGGCTTACACTGATTGCGTATT 60

************************************************* **********

P.dactylifera.BC4.Male* TTTATTTTATAAAACGGGTTAGACGTATGGCTCAGAATGGTACTATGGTTAAGCCCAATA 120

P.dactylifera.C.Khalas*** TTTATTTTATAAAACGGGTTAGACGTATGGCTCAGAATGGTACTATGGTTAAGCCCAATA 120

P.dactylifera.C.Rabiah TTTATTTTATAAAACGGGTTAGACGTATGGCTCAGAATGGTACTATGGTTAAGCCCAATA 120

P.dactylifera.C.Baydh TTTATTTTATAAAACGGGTTAGACGTATGGCTCAGAATGGTACTATGGTTAAGCCCAATA 120

P.dactylifera.C.Lulu** TTTATTTTATAAAACGGATTAGACGTATGGCTCAGAATGGTACTATGGTTAAGCCCAATA 120

P.dactylifera.C.Labana TTTATTTTATAAAACGGGTTAGACGTATGGCTCAGAATGGTACTATGGTTAAGCCCAATA 120

P.dactylifera.C.Rothanah TTTATTTTATAAAACGGGTTAGACGTATGGCTCAGAATGGTACTATGGTTAAGCCCAATA 120

P.dactylifera.C.Khalas TTTATTTTATAAAACGGGTTAGACGTATGGCTCAGAATGGTACTATGGTTAAGCCCAATA 120

P.dactylifera.C.Sukkary TTTATTTTATAAAACGGATTAGACGTATGGCTCAGAATGGTACTATGGTTAAGCCCAATA 120

P.dactylifera.Rabiah.Male TTTATTTTATAAAACGGGTTAGACGTATGGCTCAGAATGGTACTATGGTTAAGCCCAATA 120

***************** ******************************************

P.dactylifera.BC4.Male* TGATGTGTGATTATTTTATTTTATGGGCTTTAGTGCATCTTGGATGTATAGGATTGAGTC 180

P.dactylifera.C.Khalas*** TGATGTGTGATTATTTTATTTTATGGGCTTTAGTGCATCTTGGATGTATAGGATTGAGTC 180

P.dactylifera.C.Rabiah TGATGTGTGATTATTTTATTTTATGGGCTTTAGTGCATCTTGGATGTATAGGATTGAGTC 180

P.dactylifera.C.Baydh TGATGTGTGATTATTTTATTTTATGGGCTTTAGTGCATCTTGGATGTATAGGATTGAGTC 180

P.dactylifera.C.Lulu** TGATGTGTGATTATTTTATTTTATAGGCTTTGGTGCACCTTGGATGTATAGGATTGAGTC 180

P.dactylifera.C.Labana TGATGTGTGATTATTTTATTTTATGGGCTTTAGTGCATCTTGGATGTATAGGATTGAGTC 180

P.dactylifera.C.Rothanah TGATGTGTGATTATTTTATTTTATGGGCTTTAGTGCATCTTGGATGTATAGGATTGAGTC 180

P.dactylifera.C.Khalas TGATGTGTGATTATTTTATTTTATGGGCTTTAGTGCATCTTGGATGTATAGGATTGAGTC 180

P.dactylifera.C.Sukkary TGATGTGTGATTATTTTATTTTATAGGCTTTGGTGCATCTTGGATGTATAGGATTGAGTC 180

P.dactylifera.Rabiah.Male TGATGTGTGATTATTTTATTTTATGGGCTTTAGTGCATCTTGGATGTATAGGATTGAGTC 180

************************ ****** ***** **********************

P.dactylifera.BC4.Male* CTATTTAATGTGGTAATTTTAGTATGTAGGCCAGGAAACCCAATTAGGATTTTGGAGCCT 240

P.dactylifera.C.Khalas*** CTATTTAATGTGGTAATTTTAGTATGTAGGCCAGGAAACCCAATTAGGATTTTGGAGCCT 240

P.dactylifera.C.Rabiah CTATTTAATGTGGTAATTTTAGTATGTAGGCCAGGAAACCCAATTAGGATTTTGGAGCCT 240

P.dactylifera.C.Baydh CTATTTAATGTGGTAATTTTAGTATGTAGGCCAGGAAACCCAATTAGGATTTTGGAGCCT 240

P.dactylifera.C.Lulu** CTATTTAATGTGGTAATTTTAGTATGTAGGCCACGAAACCCAATTAGGGTTTTGGAGCCT 240

P.dactylifera.C.Labana CTATTTAATGTGGTAATTTTAGTATGTAGGCCAGGAAACCCAATTAGGATTTTGGAGCCT 240

P.dactylifera.C.Rothanah CTATTTAATGTGGTAATTTTAGTATGTAGGCCAGGAAACCCAATTAGGATTTTGGAGCCT 240

P.dactylifera.C.Khalas CTATTTAATGTGGTAATTTTAGTATGTAGGCCAGGAAACCCAATTAGGATTTTGGAGCCT 240

P.dactylifera.C.Sukkary CTATTTAATGTGGTAATTTTAGTATGTAGGCCAGGAAACCCAATTAGGATTTTGGAGCCT 240

P.dactylifera.Rabiah.Male CTATTTAATGTGGTAATTTTAGTATGTAGGCCAGGAAACCCAATTAGGATTTTGGAGCCT 240

********************************* ************** ***********

P.dactylifera.BC4.Male* TTAATGGGAAAGCTCTCTTATATTTCCTATATAATGGCTAGGTCCCCCTCTCCTCTCCAT 300

P.dactylifera.C.Khalas*** TTAATGGGAAAGCTCTCTTATATTTCCTATATAAAGGCTAGGTCCCCCTCTCCTCTCCAT 300

P.dactylifera.C.Rabiah TTAATGGGAAAGCTCTCTTATATTTCCTATATAAAGGCTAGGTCCCCCTCTCCTCTCCAT 300

P.dactylifera.C.Baydh TTAATGGGAAAGCTCTCTTATATTTCCTATATAAAGGCTAGGTCCCCCTCTCCTCTCCAT 300

P.dactylifera.C.Lulu** TTAATGGGAAGGCTCTCTTATATTTTCTATATAAAGGCTAGGTCCCCCTCTCCTCTCCAT 300

P.dactylifera.C.Labana TTAATGGGAAAGCTCTCTTATATTTCCTATATAAAGGCTAGGTCCCCCTCTCCTCTCCAT 300

P.dactylifera.C.Rothanah TTAATGGGAAAGCTCTCTTATATTTCCTATATAAAGGCTAGGTCCCCCTCTCCTCTCCAT 300

P.dactylifera.C.Khalas TTAATGGGAAAGCTCTCTTATATTTCCTATATAAAGGCTAGGTCCCCCTCTCCTCTCCAT 300

P.dactylifera.C.Sukkary TTAATGGGAAAGCTCTCTTATATTTCCTATATAAAGGCTAGGTCCCCCTCTCCTCTCCAT 300

P.dactylifera.Rabiah.Male TTAATGGGAAAGCTCTCTTATATTTCCTATATAAAGGCTAGGTCCCCCTCTCCTCTCCAT 300

********** ************** ******** *************************

P.dactylifera.BC4.Male* ATGGTTGATAGCTTGCCCCATTTACGGCTATCTTTTATGAGGAGCAAGGAAGGGAAGATC 360

P.dactylifera.C.Khalas*** ATGGTTGATAGCTTGCCCCATTTACGGCTATCTTTTATGAGGAGCAAGGAAGGGAAGATC 360

P.dactylifera.C.Rabiah ATGGTTGATAGCTTGCCCCATTTACGGCTATCTTTTATGAGGAGCAAGGAAGGGAAGATC 360

P.dactylifera.C.Baydh ATGGTTGATAGCTTGCCCCATTTACGGCTATCTTTTATGAGGAGCAAGGAAGGGAAGATC 360

P.dactylifera.C.Lulu** ATGGTTGATAGCTTGCCCCATTGACGGTTATCTTTTGTGAAGAGCAAGGAAGGGAAGATC 360

P.dactylifera.C.Labana ATGGTTGATAGCTTGCCCCATTTACGGCTATCTTTTATGAGGAGCAAGGAAGGGAAGATC 360

P.dactylifera.C.Rothanah ATGGTTGATAGCTTGCCCCATTTACGGCTATCTTTTATGAGGAGCAAGGAAGGGAAGATC 360

P.dactylifera.C.Khalas ATGGTTGATAGCTTGCCCCATTTACGGCTATCTTTTATGAGGAGCAAGGAAGGGAAGATC 360

P.dactylifera.C.Sukkary ATGGTTGATAGCTTGCCCCATTTACGGCTATCTTTTGTGAGGAGCAAGGAAGGGAAGATC 360

P.dactylifera.Rabiah.Male ATGGTTGATAGCTTGCCCCATTTACGGCTATCTTTTATGAGGAGCAAGGAAGGGAAGATC 360

********************** **** ******** *** *******************

P.dactylifera.BC4.Male* CAGCCGCTATTATTCCAGGTGATTGAAGAACGTATTCAATATGTCTTCTGCAGGTATATC 420

P.dactylifera.C.Khalas*** CAGCCGCTATTATTCCAGGTGATTGAAGAACGTATTCAATATGTCTTCTGCAGGTATATC 420

P.dactylifera.C.Rabiah CAGCCGCTATTATTCCAGGTGATTGAAGAACGTATTCAATATGTCTTCCGCAGGTATATC 420

P.dactylifera.C.Baydh CAGCCGCTATTATTCCAGGTGATTGAAGAACGTATTCAATATGTCTTCCGCAGGTATATC 420

P.dactylifera.C.Lulu** CAGCCGCTATTATTCCAGATGATTGAAGAACGTATTCAATATGTCTTCCGCAG------- 413

P.dactylifera.C.Labana CAGCCGCTATTATTCCAGGTGATTGAAGAACGTATTCAATATGTCTTC--CAG------- 411

P.dactylifera.C.Rothanah CAGCCGCTATTATTCCAGGTGATTGAAGAACGAGTTCA---------C--CAG------- 402

P.dactylifera.C.Khalas CAGCCGCTATTATTCCAGGTGATTGAAGAACGTATTCAATATGTCTTC------------ 408

P.dactylifera.C.Sukkary CAGCCGCTATTATTCCAGGTGATTGAAGAACGGATTCAAT-------------------- 400

P.dactylifera.Rabiah.Male CAGCCGCTATTATTCCAGGTGATTGAAGAACGTATT------------------------ 396

****************** ************* **

P.dactylifera.BC4.Male* TCTCAAACTTGAATACTGTATTATTGTGATTCTGAATCTTAGCATGTTATCATTTTTATA 480

P.dactylifera.C.Khalas*** TCTCAAACTTGAATACTGTATTATTGTGATTCTGAATCTTAGCATGTTATCATTTTTATA 480

P.dactylifera.C.Rabiah TCTCAAATTTGAATACTTTATTATTGTGATTCTGAATCTTAGCATGTTATCATTTTTATA 480

P.dactylifera.C.Baydh TCTCAAATTTGAATACTTTATTATTGTGATTCTGAATCTTAGCATGTTATCATTTTTATA 480

P.dactylifera.C.Lulu** ------------------------------------------------------------ 413

P.dactylifera.C.Labana ------------------------------------------------------------ 411

P.dactylifera.C.Rothanah ------------------------------------------------------------ 402

P.dactylifera.C.Khalas ------------------------------------------------------------ 408

P.dactylifera.C.Sukkary ------------------------------------------------------------ 400

P.dactylifera.Rabiah.Male ------------------------------------------------------------ 396

P.dactylifera.BC4.Male* ATTAACATTTGGTTAAGGAATTTTATTTCTGGACTGAAAGTTGTTGATTCCATCTCGAGA 540

P.dactylifera.C.Khalas*** ATTAACATTTGG------------------------------------------------ 492

P.dactylifera.C.Rabiah ATTAACAACCAG------------------------------------------------ 492

P.dactylifera.C.Baydh ATTAACAACCAG------------------------------------------------ 492

P.dactylifera.C.Lulu** ------------------------------------------------------------ 413

P.dactylifera.C.Labana ------------------------------------------------------------ 411

P.dactylifera.C.Rothanah ------------------------------------------------------------ 402

P.dactylifera.C.Khalas ------------------------------------------------------------ 408

P.dactylifera.C.Sukkary ------------------------------------------------------------ 400

P.dactylifera.Rabiah.Male ------------------------------------------------------------ 396

P.dactylifera.BC4.Male* CCAATTACCATCTACTGTGATAATAGTGCAGCCGTGTTCTTTTCAAAGAATAACAAGAGT 600

P.dactylifera.C.Khalas*** ------------------------------------------------------------ 492

P.dactylifera.C.Rabiah ------------------------------------------------------------ 492

P.dactylifera.C.Baydh ------------------------------------------------------------ 492

P.dactylifera.C.Lulu** ------------------------------------------------------------ 413

P.dactylifera.C.Labana ------------------------------------------------------------ 411

P.dactylifera.C.Rothanah ------------------------------------------------------------ 402

P.dactylifera.C.Khalas ------------------------------------------------------------ 408

P.dactylifera.C.Sukkary ------------------------------------------------------------ 400

P.dactylifera.Rabiah.Male ------------------------------------------------------------ 396

P.dactylifera.BC4.Male* TCTGGTGGCTCTAAGCATATAGACATCAAGTACTTGGTAGTCAGAGTTAAAGTTAAAGAA 660

P.dactylifera.C.Khalas*** ------------------------------------------------------------ 492

P.dactylifera.C.Rabiah ------------------------------------------------------------ 492

P.dactylifera.C.Baydh ------------------------------------------------------------ 492

P.dactylifera.C.Lulu** ------------------------------------------------------------ 413

P.dactylifera.C.Labana ------------------------------------------------------------ 411

P.dactylifera.C.Rothanah ------------------------------------------------------------ 402

P.dactylifera.C.Khalas ------------------------------------------------------------ 408

P.dactylifera.C.Sukkary ------------------------------------------------------------ 400

P.dactylifera.Rabiah.Male ------------------------------------------------------------ 396

P.dactylifera.BC4.Male* GGGCAGACAAAACACTACAAGAAAATACATATTCAGCGACTAAGATTCTAGTCGCCGAAT 720

P.dactylifera.C.Khalas*** ------------------------------------------------------------ 492

P.dactylifera.C.Rabiah ------------------------------------------------------------ 492

P.dactylifera.C.Baydh ------------------------------------------------------------ 492

P.dactylifera.C.Lulu** ------------------------------------------------------------ 413

P.dactylifera.C.Labana ------------------------------------------------------------ 411

P.dactylifera.C.Rothanah ------------------------------------------------------------ 402

P.dactylifera.C.Khalas ------------------------------------------------------------ 408

P.dactylifera.C.Sukkary ------------------------------------------------------------ 400

P.dactylifera.Rabiah.Male ------------------------------------------------------------ 396

P.dactylifera.BC4.Male* AACCATATTTGGTCGCCGAAATCTTTTCGCGACTAGCATACCCTTCGTCGCCGAACCTTA 780

P.dactylifera.C.Khalas*** ------------------------------------------------------------ 492

P.dactylifera.C.Rabiah ------------------------------------------------------------ 492

P.dactylifera.C.Baydh ------------------------------------------------------------ 492

P.dactylifera.C.Lulu** ------------------------------------------------------------ 413

P.dactylifera.C.Labana ------------------------------------------------------------ 411

P.dactylifera.C.Rothanah ------------------------------------------------------------ 402

P.dactylifera.C.Khalas ------------------------------------------------------------ 408

P.dactylifera.C.Sukkary ------------------------------------------------------------ 400

P.dactylifera.Rabiah.Male ------------------------------------------------------------ 396

P.dactylifera.BC4.Male* GTCAGTGAAAGATTTTGGCGACCAATATTGTGTGGTTACCTAAGGTACACCAACAACGAC 840

P.dactylifera.C.Khalas*** ------------------------------------------------------------ 492

P.dactylifera.C.Rabiah ------------------------------------------------------------ 492

P.dactylifera.C.Baydh ------------------------------------------------------------ 492

P.dactylifera.C.Lulu** ------------------------------------------------------------ 413

P.dactylifera.C.Labana ------------------------------------------------------------ 411

P.dactylifera.C.Rothanah ------------------------------------------------------------ 402

P.dactylifera.C.Khalas ------------------------------------------------------------ 408

P.dactylifera.C.Sukkary ------------------------------------------------------------ 400

P.dactylifera.Rabiah.Male ------------------------------------------------------------ 396

P.dactylifera.BC4.Male* CAACGTTTGGTTGCCGAAGAAAATTATCAGCGACCAAATCTGTTGTCGCTAATTTCAACG 900

P.dactylifera.C.Khalas*** ------------------------------------------------------------ 492

P.dactylifera.C.Rabiah ------------------------------------------------------------ 492

P.dactylifera.C.Baydh ------------------------------------------------------------ 492

P.dactylifera.C.Lulu** ------------------------------------------------------------ 413

P.dactylifera.C.Labana ------------------------------------------------------------ 411

P.dactylifera.C.Rothanah ------------------------------------------------------------ 402

P.dactylifera.C.Khalas ------------------------------------------------------------ 408

P.dactylifera.C.Sukkary ------------------------------------------------------------ 400

P.dactylifera.Rabiah.Male ------------------------------------------------------------ 396

P.dactylifera.BC4.Male* CCGACCAAATAGTTGGTCGTGGAAGGTTTGTCTTCCGCTACCAAAACAATGGTCACTGAA 960

P.dactylifera.C.Khalas*** ------------------------------------------------------------ 492

P.dactylifera.C.Rabiah ------------------------------------------------------------ 492

P.dactylifera.C.Baydh ------------------------------------------------------------ 492

P.dactylifera.C.Lulu** ------------------------------------------------------------ 413

P.dactylifera.C.Labana ------------------------------------------------------------ 411

P.dactylifera.C.Rothanah ------------------------------------------------------------ 402

P.dactylifera.C.Khalas ------------------------------------------------------------ 408

P.dactylifera.C.Sukkary ------------------------------------------------------------ 400

P.dactylifera.Rabiah.Male ------------------------------------------------------------ 396

P.dactylifera.BC4.Male* TGTCTATCTTCCCCTACCAATAGTTTGGTCGCAGAATGTATTGGAATGCATTGGCAACAA 1020

P.dactylifera.C.Khalas*** ------------------------------------------------------------ 492

P.dactylifera.C.Rabiah ------------------------------------------------------------ 492

P.dactylifera.C.Baydh ------------------------------------------------------------ 492

P.dactylifera.C.Lulu** ------------------------------------------------------------ 413

P.dactylifera.C.Labana ------------------------------------------------------------ 411

P.dactylifera.C.Rothanah ------------------------------------------------------------ 402

P.dactylifera.C.Khalas ------------------------------------------------------------ 408

P.dactylifera.C.Sukkary ------------------------------------------------------------ 400

P.dactylifera.Rabiah.Male ------------------------------------------------------------ 396

P.dactylifera.BC4.Male* ACATTTTTTAGTGGCAGAAAGTATTAACCTGTATTTAATTAACAATTCAAATTATAAGCT 1080

P.dactylifera.C.Khalas*** ------------------------------------------------------------ 492

P.dactylifera.C.Rabiah ------------------------------------------------------------ 492

P.dactylifera.C.Baydh ------------------------------------------------------------ 492

P.dactylifera.C.Lulu** ------------------------------------------------------------ 413

P.dactylifera.C.Labana ------------------------------------------------------------ 411

P.dactylifera.C.Rothanah ------------------------------------------------------------ 402

P.dactylifera.C.Khalas ------------------------------------------------------------ 408

P.dactylifera.C.Sukkary ------------------------------------------------------------ 400

P.dactylifera.Rabiah.Male ------------------------------------------------------------ 396

P.dactylifera.BC4.Male* TAATATTTATTTTTGGGCTCGTTCCAAATCCTGTACAACCAACACAGCCTAAGCCTATCC 1140

P.dactylifera.C.Khalas*** ------------------------------------------------------------ 492

P.dactylifera.C.Rabiah ------------------------------------------------------------ 492

P.dactylifera.C.Baydh ------------------------------------------------------------ 492

P.dactylifera.C.Lulu** ------------------------------------------------------------ 413

P.dactylifera.C.Labana ------------------------------------------------------------ 411

P.dactylifera.C.Rothanah ------------------------------------------------------------ 402

P.dactylifera.C.Khalas ------------------------------------------------------------ 408

P.dactylifera.C.Sukkary ------------------------------------------------------------ 400

P.dactylifera.Rabiah.Male ------------------------------------------------------------ 396

P.dactylifera.BC4.Male* AATATCCATATTGCACGATACATTTGCTCATCCAAATAGAAATATACACAATGTTGGAGA 1200

P.dactylifera.C.Khalas*** ------------------------------------------------------------ 492

P.dactylifera.C.Rabiah ------------------------------------------------------------ 492

P.dactylifera.C.Baydh ------------------------------------------------------------ 492

P.dactylifera.C.Lulu** ------------------------------------------------------------ 413

P.dactylifera.C.Labana ------------------------------------------------------------ 411

P.dactylifera.C.Rothanah ------------------------------------------------------------ 402

P.dactylifera.C.Khalas ------------------------------------------------------------ 408

P.dactylifera.C.Sukkary ------------------------------------------------------------ 400

P.dactylifera.Rabiah.Male ------------------------------------------------------------ 396

P.dactylifera.BC4.Male* ATCCATAATCATTCATTATAAATATCATCATCTAAAAGTCTAAAGTCAAGCATACCTATA 1260

P.dactylifera.C.Khalas*** ------------------------------------------------------------ 492

P.dactylifera.C.Rabiah ------------------------------------------------------------ 492

P.dactylifera.C.Baydh ------------------------------------------------------------ 492

P.dactylifera.C.Lulu** ------------------------------------------------------------ 413

P.dactylifera.C.Labana ------------------------------------------------------------ 411

P.dactylifera.C.Rothanah ------------------------------------------------------------ 402

P.dactylifera.C.Khalas ------------------------------------------------------------ 408

P.dactylifera.C.Sukkary ------------------------------------------------------------ 400

P.dactylifera.Rabiah.Male ------------------------------------------------------------ 396

P.dactylifera.BC4.Male* TATTAACCATACATCAAAATGTTTCATCCATACCATATGTTAAGGTGATAATCATCACCA 1320

P.dactylifera.C.Khalas*** ------------------------------------------------------------ 492

P.dactylifera.C.Rabiah ------------------------------------------------------------ 492

P.dactylifera.C.Baydh ------------------------------------------------------------ 492

P.dactylifera.C.Lulu** ------------------------------------------------------------ 413

P.dactylifera.C.Labana ------------------------------------------------------------ 411

P.dactylifera.C.Rothanah ------------------------------------------------------------ 402

P.dactylifera.C.Khalas ------------------------------------------------------------ 408

P.dactylifera.C.Sukkary ------------------------------------------------------------ 400

P.dactylifera.Rabiah.Male ------------------------------------------------------------ 396

P.dactylifera.BC4.Male* AACATATCAAAATGTAAATTAAAAATCCCAAAATATCTGATCTTGCTATTCATCCAAACA 1380

P.dactylifera.C.Khalas*** ------------------------------------------------------------ 492

P.dactylifera.C.Rabiah ------------------------------------------------------------ 492

P.dactylifera.C.Baydh ------------------------------------------------------------ 492

P.dactylifera.C.Lulu** ------------------------------------------------------------ 413

P.dactylifera.C.Labana ------------------------------------------------------------ 411

P.dactylifera.C.Rothanah ------------------------------------------------------------ 402

P.dactylifera.C.Khalas ------------------------------------------------------------ 408

P.dactylifera.C.Sukkary ------------------------------------------------------------ 400

P.dactylifera.Rabiah.Male ------------------------------------------------------------ 396

P.dactylifera.BC4.Male* ACCATCATGTAGACAAAAATATTCTTATACCAAAAACTGGATTCTGCTTGTAGACAACTC 1440

P.dactylifera.C.Khalas*** ------------------------------------------------------------ 492

P.dactylifera.C.Rabiah ------------------------------------------------------------ 492

P.dactylifera.C.Baydh ------------------------------------------------------------ 492

P.dactylifera.C.Lulu** ------------------------------------------------------------ 413

P.dactylifera.C.Labana ------------------------------------------------------------ 411

P.dactylifera.C.Rothanah ------------------------------------------------------------ 402

P.dactylifera.C.Khalas ------------------------------------------------------------ 408

P.dactylifera.C.Sukkary ------------------------------------------------------------ 400

P.dactylifera.Rabiah.Male ------------------------------------------------------------ 396

P.dactylifera.BC4.Male* CACCATGACCATCTGAACAAAAGCTATCCATGTACTAGAAACTAGATTTGACATGCTCAT 1500

P.dactylifera.C.Khalas*** ------------------------------------------------------------ 492

P.dactylifera.C.Rabiah ------------------------------------------------------------ 492

P.dactylifera.C.Baydh ------------------------------------------------------------ 492

P.dactylifera.C.Lulu** ------------------------------------------------------------ 413

P.dactylifera.C.Labana ------------------------------------------------------------ 411

P.dactylifera.C.Rothanah ------------------------------------------------------------ 402

P.dactylifera.C.Khalas ------------------------------------------------------------ 408

P.dactylifera.C.Sukkary ------------------------------------------------------------ 400

P.dactylifera.Rabiah.Male ------------------------------------------------------------ 396

P.dactylifera.BC4.Male* AACTCCACCATGTATTTGAGAAAGCAAAAAAGAATACACAATCAATAACCACATATGAAA 1560

P.dactylifera.C.Khalas*** ------------------------------------------------------------ 492

P.dactylifera.C.Rabiah ------------------------------------------------------------ 492

P.dactylifera.C.Baydh ------------------------------------------------------------ 492

P.dactylifera.C.Lulu** ------------------------------------------------------------ 413

P.dactylifera.C.Labana ------------------------------------------------------------ 411

P.dactylifera.C.Rothanah ------------------------------------------------------------ 402

P.dactylifera.C.Khalas ------------------------------------------------------------ 408

P.dactylifera.C.Sukkary ------------------------------------------------------------ 400

P.dactylifera.Rabiah.Male ------------------------------------------------------------ 396

P.dactylifera.BC4.Male* AATAATAAACTAAAATTCAAGTATAAGTTCACAATGACATTTCGCTTAATTTGGTCCAAA 1620

P.dactylifera.C.Khalas*** ------------------------------------------------------------ 492

P.dactylifera.C.Rabiah ------------------------------------------------------------ 492

P.dactylifera.C.Baydh ------------------------------------------------------------ 492

P.dactylifera.C.Lulu** ------------------------------------------------------------ 413

P.dactylifera.C.Labana ------------------------------------------------------------ 411

P.dactylifera.C.Rothanah ------------------------------------------------------------ 402

P.dactylifera.C.Khalas ------------------------------------------------------------ 408

P.dactylifera.C.Sukkary ------------------------------------------------------------ 400

P.dactylifera.Rabiah.Male ------------------------------------------------------------ 396

P.dactylifera.BC4.Male* TCTCTAAATAACCTACATAATATAAATGAGAATACATACCAACGAGTATTTGTACCACTA 1680

P.dactylifera.C.Khalas*** ------------------------------------------------------------ 492

P.dactylifera.C.Rabiah ------------------------------------------------------------ 492

P.dactylifera.C.Baydh ------------------------------------------------------------ 492

P.dactylifera.C.Lulu** ------------------------------------------------------------ 413

P.dactylifera.C.Labana ------------------------------------------------------------ 411

P.dactylifera.C.Rothanah ------------------------------------------------------------ 402

P.dactylifera.C.Khalas ------------------------------------------------------------ 408

P.dactylifera.C.Sukkary ------------------------------------------------------------ 400

P.dactylifera.Rabiah.Male ------------------------------------------------------------ 396

P.dactylifera.BC4.Male* CTTGAATACATAAACCCCTCCTCACAAAACCACTCTCATACGCACACAAATAGCACCATA 1740

P.dactylifera.C.Khalas*** ------------------------------------------------------------ 492

P.dactylifera.C.Rabiah ------------------------------------------------------------ 492

P.dactylifera.C.Baydh ------------------------------------------------------------ 492

P.dactylifera.C.Lulu** ------------------------------------------------------------ 413

P.dactylifera.C.Labana ------------------------------------------------------------ 411

P.dactylifera.C.Rothanah ------------------------------------------------------------ 402

P.dactylifera.C.Khalas ------------------------------------------------------------ 408

P.dactylifera.C.Sukkary ------------------------------------------------------------ 400

P.dactylifera.Rabiah.Male ------------------------------------------------------------ 396

P.dactylifera.BC4.Male* TCTATTCCACAAGTATTAACCTTGATAAGAAAATTGTGCAAGAAACAGCCAGGTGATGAT 1800

P.dactylifera.C.Khalas*** ------------------------------------------------------------ 492

P.dactylifera.C.Rabiah ------------------------------------------------------------ 492

P.dactylifera.C.Baydh ------------------------------------------------------------ 492

P.dactylifera.C.Lulu** ------------------------------------------------------------ 413

P.dactylifera.C.Labana ------------------------------------------------------------ 411

P.dactylifera.C.Rothanah ------------------------------------------------------------ 402

P.dactylifera.C.Khalas ------------------------------------------------------------ 408

P.dactylifera.C.Sukkary ------------------------------------------------------------ 400

P.dactylifera.Rabiah.Male ------------------------------------------------------------ 396

P.dactylifera.BC4.Male* CAAAGTTAAGCTGCAGATTTAAGAAATAAACTTGAAAGGAAAAGAACATGATATCAGATT 1860

P.dactylifera.C.Khalas*** ------------------------------------------------------------ 492

P.dactylifera.C.Rabiah ------------------------------------------------------------ 492

P.dactylifera.C.Baydh ------------------------------------------------------------ 492

P.dactylifera.C.Lulu** ------------------------------------------------------------ 413

P.dactylifera.C.Labana ------------------------------------------------------------ 411

P.dactylifera.C.Rothanah ------------------------------------------------------------ 402

P.dactylifera.C.Khalas ------------------------------------------------------------ 408

P.dactylifera.C.Sukkary ------------------------------------------------------------ 400

P.dactylifera.Rabiah.Male ------------------------------------------------------------ 396

P.dactylifera.BC4.Male* ATAAAGGAAGCGAGCATAAATGATTAAAACTGATCTGCATCGTAGGCACCAAAATGCACA 1920

P.dactylifera.C.Khalas*** ------------------------------------------------------------ 492

P.dactylifera.C.Rabiah ------------------------------------------------------------ 492

P.dactylifera.C.Baydh ------------------------------------------------------------ 492

P.dactylifera.C.Lulu** ------------------------------------------------------------ 413

P.dactylifera.C.Labana ------------------------------------------------------------ 411

P.dactylifera.C.Rothanah ------------------------------------------------------------ 402

P.dactylifera.C.Khalas ------------------------------------------------------------ 408

P.dactylifera.C.Sukkary ------------------------------------------------------------ 400

P.dactylifera.Rabiah.Male ------------------------------------------------------------ 396

P.dactylifera.BC4.Male* TTTCTTAAGTTAATAAATGGAAACAATTGTCCATAACAAATGGAAGCTATTGTAAGTAAC 1980

P.dactylifera.C.Khalas*** ------------------------------------------------------------ 492

P.dactylifera.C.Rabiah ------------------------------------------------------------ 492

P.dactylifera.C.Baydh ------------------------------------------------------------ 492

P.dactylifera.C.Lulu** ------------------------------------------------------------ 413

P.dactylifera.C.Labana ------------------------------------------------------------ 411

P.dactylifera.C.Rothanah ------------------------------------------------------------ 402

P.dactylifera.C.Khalas ------------------------------------------------------------ 408

P.dactylifera.C.Sukkary ------------------------------------------------------------ 400

P.dactylifera.Rabiah.Male ------------------------------------------------------------ 396

P.dactylifera.BC4.Male* AGAACTGGTATCAAGCTGTTTCCAGAGCAAATTGACTCGTTTTCCCTTGACAGGTGAGTG 2040

P.dactylifera.C.Khalas*** ------------------------------------------------------------ 492

P.dactylifera.C.Rabiah ------------------------------------------------------------ 492

P.dactylifera.C.Baydh ------------------------------------------------------------ 492

P.dactylifera.C.Lulu** ------------------------------------------------------------ 413

P.dactylifera.C.Labana ------------------------------------------------------------ 411

P.dactylifera.C.Rothanah ------------------------------------------------------------ 402

P.dactylifera.C.Khalas ------------------------------------------------------------ 408

P.dactylifera.C.Sukkary ------------------------------------------------------------ 400

P.dactylifera.Rabiah.Male ------------------------------------------------------------ 396

P.dactylifera.BC4.Male* AAACAGACATTTTATTGCTAATCTGCCCCTCACAAGTCTGGATATAATAAAATATCCTGA 2100

P.dactylifera.C.Khalas*** ------------------------------------------------------------ 492

P.dactylifera.C.Rabiah ------------------------------------------------------------ 492

P.dactylifera.C.Baydh ------------------------------------------------------------ 492

P.dactylifera.C.Lulu** ------------------------------------------------------------ 413

P.dactylifera.C.Labana ------------------------------------------------------------ 411

P.dactylifera.C.Rothanah ------------------------------------------------------------ 402

P.dactylifera.C.Khalas ------------------------------------------------------------ 408

P.dactylifera.C.Sukkary ------------------------------------------------------------ 400

P.dactylifera.Rabiah.Male ------------------------------------------------------------ 396

P.dactylifera.BC4.Male* TTTAAAAAGAATGGAGTTCAATTGAAACATCAATGAGTGATGATGTCCTAAGCAGGCGTG 2160

P.dactylifera.C.Khalas*** ------------------------------------------------------------ 492

P.dactylifera.C.Rabiah ------------------------------------------------------------ 492

P.dactylifera.C.Baydh ------------------------------------------------------------ 492

P.dactylifera.C.Lulu** ------------------------------------------------------------ 413

P.dactylifera.C.Labana ------------------------------------------------------------ 411

P.dactylifera.C.Rothanah ------------------------------------------------------------ 402

P.dactylifera.C.Khalas ------------------------------------------------------------ 408

P.dactylifera.C.Sukkary ------------------------------------------------------------ 400

P.dactylifera.Rabiah.Male ------------------------------------------------------------ 396

P.dactylifera.BC4.Male* A 2161

P.dactylifera.C.Khalas*** - 492

P.dactylifera.C.Rabiah - 492

P.dactylifera.C.Baydh - 492

P.dactylifera.C.Lulu** - 413

P.dactylifera.C.Labana - 411

P.dactylifera.C.Rothanah - 402

P.dactylifera.C.Khalas - 408

P.dactylifera.C.Sukkary - 400

P.dactylifera.Rabiah.Male - 396

**Figure S3.** Multiple DNA sequence alignment comparing Ibn Majid Long terminal repeat (LTR) retrotransposon nucleotide sequences assembled for (VIR^IM^) allele of different yellow date palm cultivars. Nucleotide sequences from yellow cultivars Rothanah, Rabiah, Labana, Baydh, Sukkary, Khalas and Male originated from Rabiah aligned with BC4 Male* (Hazzouri et al., 2019), Lulu**(Hazzouri et al., 2015) and Khalas*** (Al-Mssallem et al., 2013). The sixteen nucleotides at the beginning of the sequences are part of exon 3 before the Ibn Majid LTR start.


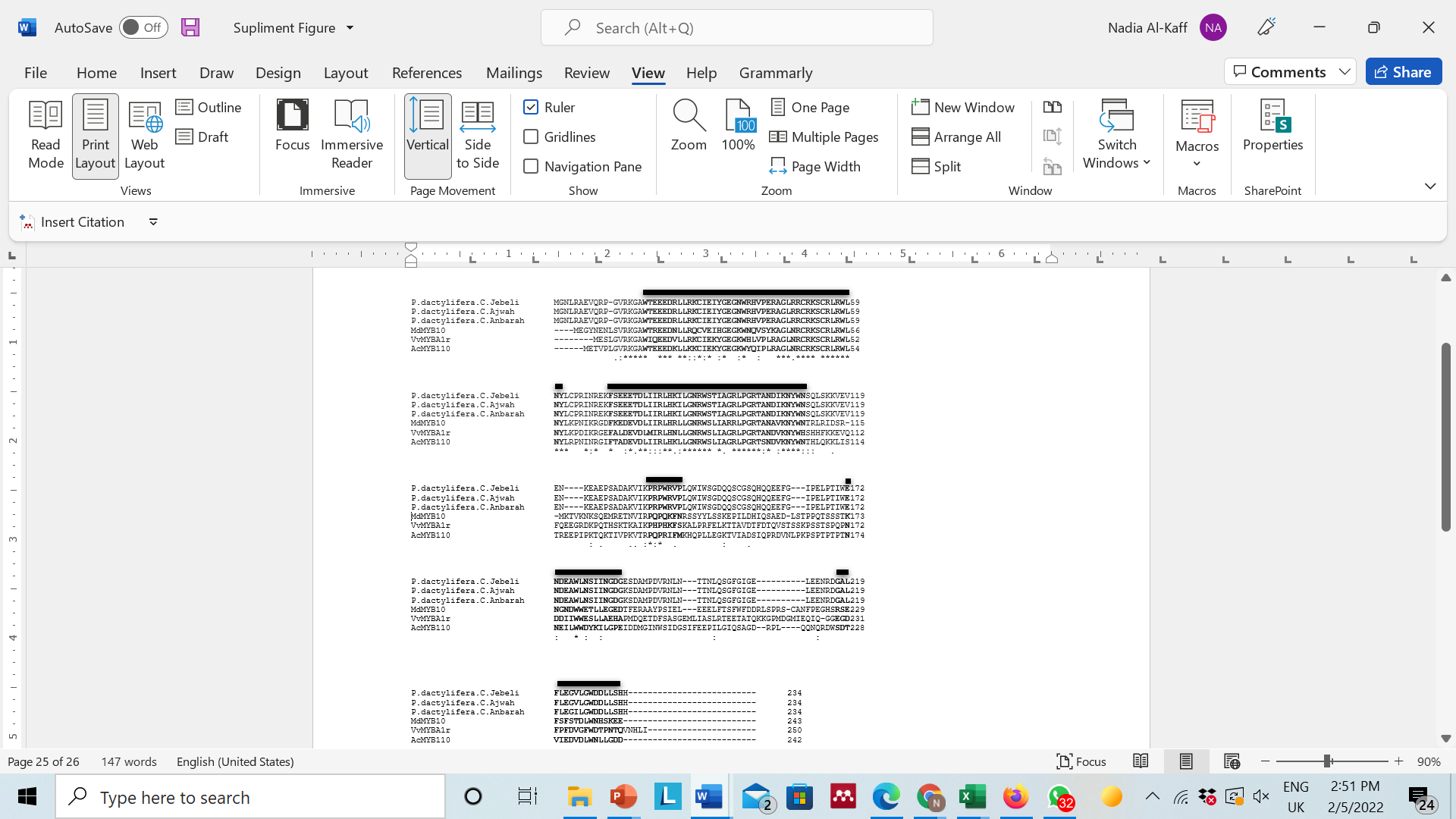


**R2**

**R3**

**S6A**

**S6B**

**S6C**

Figure S4. Assigning date palm VIR^+^ R2R3 MYB C terminus motifs. Amino acid sequences of VIR+ for cultivars Ajwah, Anbarah and Jebeli Aligned to moderate (MdMYB10) and strong (AcMYB110, VvMYBA1r) anthocyanin activators ^51^.

**Table S1.** Plant species, common name, gene ID that resulted from Blast search with VIR^+^ of date palm in the NCBI database ([www.ncbi.nlm.nih.gov](http://www.ncbi.nlm.nih.gov)) and used to produce the phylogenetic tree

|  | | Species | | Common name | Class | NCBI gene ID |
| --- | --- | --- | --- | --- | --- | --- |
| *1* | *A.cepa.MYB1* | | Onion | | Monocot | KX785130 |
| *2* | *A.chinensis.MYB* | | Kiwi | | Dicot | KF157390 |
| *3* | *A.hypogaea.MYB113* | | Peanut | | Dicot | XM_025839637 |
| *4* | *C.maxima.MYB1-like* | | Squash | | Dicot | XM_023141785 |
| *5* | *C.medica.Ruby* | | Citron | | Dicot | ANI87836.1 |
| 6 | *C.melo.MYB90-like* | | Muskmelon | | Dicot | XP_008441809.1 |
| *7* | *C.sinensis.MYB114-like* | | Tea plant | | Dicot | XP_028068989.1 |
| *8* | *E.guineensis.MYB113-like* | | Oil palm | | Monocot | XP_010932017.2 |
|  | *E.guineensis.MYB1-like* | | Oil palm | | Monocot | XP_010932028.1 |
|  | *E.guineensis.VIR* | | Oil palm | | Monocot | XP_010931211.1 |
| *9* | *M.domestica.MYB113-like* | | Apple | | Dicot | NP_001315777.1 |
| *10* | *M.notabilis.MYB113-like* | | Mulberry | | Dicot | XP_024026656.1 |
| *11* | *M.hybrid.MYB10* | | Crabapples | | Dicot | AFP89357.1 |
| *12* | *M.pumila.MYB10* | | Cultivated apple | | Dicot | ADB22516.1 |
| *13* | *M.rubra.MYB1* | | Red mulberry | | Dicot | ADG21957.1 |
| *14* | *O.europaea.MYB113-like* | | Olive | | Dicot | XP_022894857.1 |
| *15* | *P.avium.MYB75-like* | | Sweet cherry | | Dicot | XM_021956282 |
| *16* | *P.bretschneideri.MYB75* | | Chinese white pear | | Dicot | XP_018505935.1 |
| *17* | *P.cerasifera.MYB10.1* | | Cherry plum | | Dicot | AKV89247.1 |
| *18* | *P.dactylifera.C.Khalas**** | | Date palm | | Monocot | LOC103717680 |
|  | *P.dactylifera.C.Khenezi*** | | Date palm | | Monocot | KT734805 |
|  | *P.dactylifera.C.Lulu*** | | Date palm | | Monocot | KT734804 |
|  | *P.dactylifera.BC4.Male** | | Date palm | | Monocot | Pdac_HC_chr4T0137100 |
| *19* | *P.mume****.****PAP1-like* | | Japanese apricot | | Dicot | AUB13346.1 |
| *20* | *P.persica.MYB10* | | Peach | | Dicot | ADK73605.1 |
| *21* | *P.pyrifolia.MYB10* | | Asian Pear | | Dicot | ADN26574.1 |
| *22* | *P.salicina.MYB10* | | Japanese plum | | Dicot | KX349091 |
| *23* | *R.nigrum.MYB10* | | Blackcurrant | | Dicot | KY786107 |
| *24* | *R.rubrum.MYB10* | | Red currant | | Dicot | KY786108 |
| *2*5 | *S.melongena.MYB1* | | Egg plant | | Dicot | KT259043 |
| *26* | *S.oleosum.MYB113-like* | | Blue cherry | | Dicot | XP_030461131.1 |
| *27* | *S.tuberosum.MYBA1* | | Purple Potato | | Dicot | KP317177 |
| *28* | *T.cacao.MYB75* | | Cacao | | Dicot | XM_007032976 |
| *29* | *V.corymbosum.MYB1* | | Northern highbush blueberry | | Dicot | KT225487 |
| *30* | *Z.jujuba.MYB113-like* | | Jujube | | Dicot | XM_016046190 |
| *31* | *V.vinifera.MYB90* | | Grape vine | | Dicot | XP_002274992.2 |
| 32 | *P.trichocarpa.MYB90* | | black cottonwood | | Dicot | XP_024444002.1 |
| 33 | *A.formosa.MYB17* | | Red columbine | | Dicot | ACQ82820.1 |

**Notes** This list used to produce the phylogenetic tree by MUSCLE. Date palm *VIR* gene Sequences used as references are marked with (*),(**) and (***).

A.cepa.MYB1 MYHSNCNYIMAAPACSSSSSSLVRKNNRDVMRKKESDGVRKGAWSREEDELLKKCIQKYG

S.tuberosum.MYB113-like -------------------------MNTPMCASL---GVRKGSWTEQEDFLLRKCIQIYG

S.tuberosum.MYBA1 -------------------------MNTPMCASL---GVRKGSWTEQEDFLLRKCIQIYG

S.melongena.MYB1 -------------------------MNNPPIICTSV-RVRKGSWTEEEDLLLRKCMEKYG

T.cacao.MYB75 -------------------------MYRKPMEGLSL-GVRKGAWTEEEDILLKKCIEKYG

P.pyrifolia.MYB10 -------------------------MEGYNVNL----SVRKGAWTREEDNLLRQCIEIHG

P.bretschneideri.MYB75 -------------------------MEGYNVNL----SVRKGAWTREEDNLLRQCIEIHG

M.pumila.MYB10 -------------------------MEGYNENL----SVRKGAWTREEDDLLRQCIEIHG

M.hybrid.MYB10 -------------------------MEGYNENL----SVRKGAWTREEDNLLRQCVEIHG

M.domestica.MYB113-like -------------------------MEGYNENL----SVRKGAWTREEDNLLRQCVEIHG

P.mume.PAP1-like -------------------------MEGYNL------GVRKGAWTREEDDLLRQCIEKQG

P.persica.MYB10 -------------------------MEGYNL------GVRKGAWTREEDDLLRQCIENHG

P.cerasifera.MYB10.1 -------------------------MEGSNL------GVRKGAWTTEEDDLLRQCIENHG

P.salicina.MYB10 -------------------------MEGYNL------GVRKGAWTREEDDLLRQCIEKQG

P.avium.MYB75-like -------------------------MEGNNL------DVRKGAWTKEEDGLLKQCIENHG

M.notabilis.MYB113-like -------------------------MHTEGCSSTYGRG-EKSAWTREEDILLKKCIDKYG

M.rubra.MYB1 ------------------------MEGSL--------GVRKGAWTVEEDTLLKLYIEKYG

O.europaea.MYB113-like -------------------------MHSM---VSTPVEVRKGSWTEEEDNLLRKCIDKYG

R.rubrum.MYB10 -------------------------MERDFMDEKSSFGVRKGAWTSEEDVLLKNCIDKYG

R.nigrum.MYB10 -------------------------MERDFMDEKSGFGVRKGAWTSEEDVLLKNCIDKYG

A.chinensis.MYB -------------------------MESVTL------GVRKGAWTEEGDKLLKKCIEKYG

C.sinensis.MYB114-like -------------------------MEGVPL------GVRKGAWTEEEDNLLKKCIETNG

V.corymbosum.MYB1 -----------------------------------PLGVRKGAWTEEEDCLLKKCIEKHG

P.trichocarpa.MYB90 ------------------------MVSSL--------GVRKGAWTEEEDILLRKCVEKYG

V.vinifera. ---------------MLLLVIVDYRLRTTKLFMDGPSGVRKGAWTREEDVLLRTCIEKYG

C.medica.Ruby ------------------------MADSL--------GVRKGAWTGEEDDLLRKCIEKYG

S.oleosum. ------------------------MKGAI--------GVRKGAWTEEEDVLLKKCVEKYG

A.formosa.MYB17 -------------------------MRNS--------NVRKGAWNQDEDLLLKRCIEKYG

Z.jujuba.MYB113-like -------------------------MG----------GI---AWTEEEDNLLKKCIRQYG

A.hypogaea.MYB113 -------------------------MG----------GV---AWTEEEDHLLKKCIQQYG

E.guineensis.MYB113-like -------------------------MPSLRAQTRQP-GIRKGAWTVEEDALLRRCVEKYG

C.melo.MYB90-like -------------------------MG----------GI---AWTEEEDYLLKKCIEQYG

C.maxima.MYB1-like -------------------------MG----------GI---AWTEEEDYLLKKCIEQYG

E.guineensis.MYB1-like -------------------------MGNLRAEVQRP-GVRKGAWSEEEDRLLRKCIEKYG

E.guineensis.MYB1 -------------------------MGNLRAEVQRPAGVRKGAWSEEEDRLLRKCVEKYG

P.dactylifera.C.Khenezi** -------------------------MGNLRAEVQRP-XVRKGAWTEEEDRLLRKCIEIYG

P.dactylifera.C.Anbarah -------------------------MGNLRAEVQRP-GVRKGAWTEEEDRLLRKCIEIYG

P.dactylifera.C.Safawi -------------------------MGNLRAEVQRP-GVRKGAWTEEEDRLLRKCIEIYG

P.dactylifera.C.Jebeli -------------------------MGNLRAEVQRP-GVRKGAWTEEEDRLLRKCIEIYG

P.dactylifera.C.Hilwah -------------------------MGNLRAEVQRP-GVRKGAWTEEEDRLLRKCIEIYG

P.dactylifera.C.Shalaby -------------------------MGNLRAEVQRP-GVRKGAWTEEEDRLLRKCIEIYG

P.dactylifera.C.Ajwah -------------------------MGNLRAEVQRP-GVRKGAWTEEEDRLLRKCIEIYG

P.dactylifera.C.Khalas*** -------------------------MGNLRAEVQHP-GVRKGAWTEEEDRLLRKCIEIYG

P.dactylifera.C.Lulu** -------------------------MGNLRAEVQRP-GVRKGAWTEEEDRLLRKCIEIYG

P.dactylifera.BC4.Male* -------------------------MGNLRAEVQRP-GVRKGAWTEEEDRLLRKCIEIYG

P.dactylifera.C.Rothanah -------------------------MGNLRAEVQHP-GVRKGAWTEEEDRLLRKCIEIYG

P.dactylifera.C.Rabiah -------------------------MGNLRAEVQHP-GVRKGAWTEEEDRLLRKCIEIYG

P.dactylifera.C.Baydh -------------------------MGNLRAEVQHP-GVRKGAWTEEEDRLLRKCIEIYG

P.dactylifera.C.Khalas -------------------------MGNLRAEVQHP-GVRKGAWTEEEDRLLRKCIEIYG

P.dactylifera.Rabiah.Male -------------------------MGNLRAEVQHP-GVRKGAWTEEEDRLLRKCIEIYG

P.dactylifera.C.Labana -------------------------MGNLRAEVQRP-GVRKGAWTEEEDRLLRKCIEIYG

P.dactylifera.C.Sukkary -------------------------MGNLRAEVQRP-GVRKGAWTEEEDRLLRKCIEIYG

:*. : * **. : *

A.cepa.MYB1 EGKWSSVPSRAGLKRCRKSCRLRWLNYLSPAIHRGKFKDDEIDLIFRLHKLLGNRWSLIA

S.tuberosum.MYB113-like EGKWHLVPARAGLNRCRKSCRLRWLNYLRPHIKRGDFAPDEVDLILRLHKLLGNRWSLIA

S.tuberosum.MYBA1 EGKWHLVPARAGLNRCRKSCRLRWLNYLRPHIKRGDFAPDEVDLILRLHKLLGNRWSLIA

S.melongena.MYB1 EGKWHLVPARAGLNRCRKSCRLRWLNYLRPHIKRGDFASDEVDLILRLHKLLGNRWSLIA

T.cacao.MYB75 EGKWHQVPSKAGLNRCRKSCRLRWLNYLKPNIKRGDFAADEVDLIIRLHKLLGNRWSLIA

P.pyrifolia.MYB10 EGKWNQVSYKAGLNRCRKSCRQRWLNYLKPNIKRGDFKEDEVDLILRLHKLLGNRWSLIA

P.bretschneideri.MYB75 EGKWNQVSYKAGLNRCRKSCRQRWLNYLKPNIKRGDFKEDEVDLILRLHRLLGNRWSLIA

M.pumila.MYB10 EGKWRQLPNKAGLNTCRKSCRLRWLNYLKPNIKRGDFKEDEVDLIIRLHRLLGNRWSLIA

M.hybrid.MYB10 EGKWNQVSYKAGLNRCRKSCRQRWLNYLKPNIKRGDFKEDEVDLIIRLHRLLGNRWSLIA

M.domestica.MYB113-like EGKWNQVSYKAGLNRCRKSCRQRWLNYLKPNIKRGDFKEDEVDLIIRLHRLLGNRWSLIA

P.mume.PAP1-like EGKWHQVPYKAGLSRCRKSCRLRWLNYLKPNIKRGDFT-EEVDLIIRLHKLLGNRWSLIA

P.persica.MYB10 EGKWHQVPNKAGLNRCRKSCRLRWMNYLKPNIKRGEFAEDEVDLIIRLHKLLGNRWSLIA

P.cerasifera.MYB10.1 EGKWHQVPNKAGLNRCRKSCRLRWLNYLKPNIKRGDFAEDEVDLIIRLHKLLGNRWSLIA

P.salicina.MYB10 EGKWHQVPYKAGLSRCRKSCRLRWLNYLKPNIKRGDFMEDEVDLIIRLHKLLGNRWSLIA

P.avium.MYB75-like EGKWHQVPYKAGLKRCRKSCRLRWLNYLKPNIKRGEFAEDEVDLIVRLHKLLGNRWSLIA

M.notabilis.MYB113-like EGKWQLAPSRAGLNRCRKSCRLRWMNYLKPDIKRGEFTADEVALILRLHKLLGNRWAMIA

M.rubra.MYB1 EGKWHQVPPRAGLNRCRKSCRLRWLNYLKPNIKRGEFKADEVDLMIRLHKLLGNRWSMIA

O.europaea.MYB113-like EGKWHQVPLRAGLNRCRKSCRLRWLNYLSPNIKRGDFTPDEDDLIIRLHNLLGNRWSLIS

R.rubrum.MYB10 EGKWHQVPFRAGLNRCRKSCRLRWLNYLRPNIKRGEFTFDEVDLIIRLHKLLGNRWSLIA

R.nigrum.MYB10 EGKWHQVPFRAGLNRCRKSCRLRWLNYLRPNIKRGEFTFDEVDLIIRLHKLLGNRWSLIA

A.chinensis.MYB EGKWHQVPLRSGLNRCRKSCRMRWLNYLRPNINRGNFTADEVDLIIRLHKLLGNRWSLIA

C.sinensis.MYB114-like EGKWHQVPFKAGLNRCRKSCRLRWLNYLRPNIKRGSFGVDEVDLIVRLHKLLGNRWSLIA

V.corymbosum.MYB1 EGKWHQVPYRAGLNRCRKSCRLRWLNYLRPNIKRGNFAVDEVDLIIRLHKLLGNRWSLIA

P.trichocarpa.MYB90 EGRWCQIPLQAGLNRCRKSCRMRWLNYLKPNVNRGQFSVGEVDLIIRLHKLLGNRWSLIA

V.vinifera. EGKWHQVPFRSGLNRCRKSCRLRWLNYLRPDIKRGKFKTDEVDLIIRLHKLLGNRWALIA

C.medica.Ruby EAKWHQVPLRAGLHRCRKSCRLRWLNYLKPNIKRGEFAADEVDLILRLHKLLGNRWSLIA

S.oleosum. EGNWHLVPHRAGLNRCRKSCRLRWLNYLKPNIKRGAFQEDEVDMIIRLHKLLGNRWSLIA

A.formosa.MYB17 EGNWHQVPMRAGLNRCRKSCRLRWLNYLHPNIKRGGFKEDEIDLIMKLHKLLGNRWSLIA

Z.jujuba.MYB113-like EGKWHRVPLLAGLKRCRKSCRLRWLNYLRPNIKRGQFSHEEVDLIIKLHRLLGNRWSLIA

A.hypogaea.MYB113 EGKWHRVPLLAGLNRCRKSCRLRWLNYLRPNIKRGNFAEEEVEMIVKLHKLLGNRWSLIA

E.guineensis.MYB113-like ATEWRHVPLRAGLNRCRKSCRLRWLNYLRPGINRGSFGEDETDLIVRLHKLLGNRWSLIA

C.melo.MYB90-like EGKWHRVPQLAGLNRCRKSCRLRWLNYLRPNIKRGSFTPQEVDLILNLHNLLGNRWSIIA

C.maxima.MYB1-like EGKWHRVPQLAGLNRCRKSCRLRWLNYLRPNIKRGSFTPEEVELILNLHNILGNRWSIIA

E.guineensis.MYB1-like EGKWRHVPQMAGLRRCRKRCRLRWLNYLRRRINRATFAEEETDLIVKLHKLLGNRWSLIV

E.guineensis.MYB1 EGNWRHVPQRAGLRRCRKSCRLRWLNYLSPRINREKFSEEETDLIIRLHKLLGNRWSLIA

P.dactylifera.C.Khenezi** EGNWRHVPERAGLRRCRKSCRLRWLXYLCPRINREKFSEEETDLIIRLHKILGNRWSTIA

P.dactylifera.C.Anbarah EGNWRHVPERAGLRRCRKSCRLRWLNYLCPRINREKFSEEETDLIIRLHKILGNRWSTIA

P.dactylifera.C.Safawi EGNWRHVPERAGLRRCRKSCRLRWLNYLCPRINREKFSEEETDLIIRLHKILGNRWSTIA

P.dactylifera.C.Jebeli EGNWRHVPERAGLRRCRKSCRLRWLNYLCPRINREKFSEEETDLIIRLHKILGNRWSTIA

P.dactylifera.C.Hilwah EGNWRHVPERAGLRRCRKSCRLRWLNYLCPRINREKFSEEETDLIIRLHKILGNRWSTIA

P.dactylifera.C.Shalaby EGNWRHVPERAGLRRCRKSCRLRWLNYLCPRINREKFSEEETDLIIRLHKILGNRWSTIA

P.dactylifera.C.Ajwah EGNWRHVPERAGLRRCRKSCRLRWLNYLCPRINREKFSEEETDLIIRLHKILGNRWSTIA

P.dactylifera.C.Khalas*** EGNWRHVPGRAGLRRCRKSCRLRWLNYLCPRINREKFSEEETDLIIRLHKILGNRWSTIA

P.dactylifera.C.Lulu** EGNWRHVPERAGLRRCRKSCRLRWLNYLXPRINREKFSEEETDLIIRLHKILGNRWSTIA

P.dactylifera.BC4.Male* EGNWRHVPERAGLRRCRKSCRLRWLNYLCPRINREKFSEEETDLIIRLHKILGNRWSTIA

P.dactylifera.C.Rothanah EGNWRHVPERAGLRRCRKSCRLRWLNYLCPRINREKFSEEETDLIIRLHKILGNRWSTIA

P.dactylifera.C.Rabiah EGNWRHVPERAGLRRCRKSCRLRWLNYLCPRINREKFSEEETDLIIRLHKILGNRWSTIA

P.dactylifera.C.Baydh EGNWRHVPERAGLRRCRKSCRLRWLNYLCPRINREKFSEEETDLIIRLHKILGNRWSTIA

P.dactylifera.C.Khalas EGNWRHVPERAGLRRCRKSCRLRWLNYLCPRINREKFSEEETDLIIRLHKILGNRWSTIA

P.dactylifera.Rabiah.Male EGNWRHVPERAGLRRCRKSCRLRWLNYLCPRINREKFSEEETDLIIRLHKILGNRWSTIA

P.dactylifera.C.Labana EGNWRHVPERAGLRRCRKSCRLRWLNYLCPRINREKFSEEETDLIIRLHKILGNRWSTIA

P.dactylifera.C.Sukkary EGNWRHVPERAGLRRCRKSCRLRWLNYLCPRINREKFSEEETDLIIRLHKILGNRWSTIA

* . :** *** ** **: ** ::* * * ::..**.:*****: *

A.cepa.MYB1 GRIPGRTANDIKNFWNTHLSKKKL--------------TTHEFPKNQDKVGSSIHHHRVQ

S.tuberosum.MYB113-like GRLPGRTANDVKNYWNTHFQKKLN-IIAPPPPRPRPNHHLQIKHKSITVNKNEIIRPQPR

S.tuberosum.MYBA1 GRLPGRTANDVKNYWNTHFQKKLNIIAPPPPPRPRPNHHLQIKHKSITVNKNEIIRPQPR

S.melongena.MYB1 GRLPGRTANDVKNYWNTNLLRKFT-----------IAPQKINNTCKDIISTNEIIRPQPR

T.cacao.MYB75 GRLPGRTANDVKNYWNTHLLKKFN----------PSKQKARDDDQNPSNPNNIVIKPRPR

P.pyrifolia.MYB10 RRLPGRTANDVKNYWNTRLRIDSR--------------MKTLKNKSQETRKTNVIRPQPQ

P.bretschneideri.MYB75 RRLPGRTANDVKNYWNTRLGIDSR--------------MKTLKNKSQETRKTNVIRPQPQ

M.pumila.MYB10 RRLPGRTANAVKNYWNTRLRIDSR--------------MKTVKNKSQEMRETNVIRPQPQ

M.hybrid.MYB10 RRLPGRTANAVKNYWNTRLRIDSR--------------MKTVKNKSQEMRETNVIRPQPQ

M.domestica.MYB113-like RRLPGRTANAVKNYWNTRLRIDSR--------------MKTVKNKSQEMRETNVIRPQPQ

P.mume.PAP1-like GRLPGRTANDVKNYWNTRLRTDSR--------------LKKLKDKTQETIKIIVIRPQPR

P.persica.MYB10 GRLPGRTANDVKNYWNTRLRTDSR--------------LKKVKDKPQETIKTIVIRPQPR

P.cerasifera.MYB10.1 GRLPGRTANDVKNYWNTRLRMDSR--------------LKKVKDKPQETIKAIVIRPQPR

P.salicina.MYB10 RRLPGRTANDVKNYWNTRLRTDYC--------------MKKMKDKSQETIKT-IIRPQPR

P.avium.MYB75-like GRLPGRTSNGVKNYWNTRLRTNSL-------------VKRTTKEKSQETIKTMVIRPQPR

M.notabilis.MYB113-like GRIPGRTANDVKNYWNTHLRKKVA---------LFPHDKNTKNTNNDIIAKSNVFKPRPR

M.rubra.MYB1 GRLPGRTANDVKNYWNTHLRKNAI------------SRIKDGGEKAQQTSKVNIIKPRPR

O.europaea.MYB113-like GRLPGRTANDVKNHWNSHVQKKVL-------------AQDEATRKAQKTTKTTILKPRPR

R.rubrum.MYB10 GRIPGRTANDVKNYWNTNLRKKMI-----------------SQPRVEITEKTRVIRPQPR

R.nigrum.MYB10 GRIPGRTANDVKNYWNTNLRKKMI-----------------SQPRVEITEKTKVVRPQPR

A.chinensis.MYB GRLPGRTSNDVKNYWNTHLQKKLI------------------------------TYPRAQ

C.sinensis.MYB114-like GRLPGRTANDVKNYWNTHLQKKLI------------PQIEVVKVKTPRMMETKAIRPRPR

V.corymbosum.MYB1 GRLPGRTSNDVKNYWNTHLKEKST---------DQSGEVQKSKTTLNTTERTTIIRPQPR

P.trichocarpa.MYB90 GRLPGRTANDVKNYWNTNLRKKVV----------SSTREAQTEPEPKAITKDNIIKPRPR

V.vinifera. GRLPGRTSNDVKNYWNTHLSKKFF--------------GKAVKDVADKTAKVTVIKPRPR

C.medica.Ruby GRLPGRTANDVKNFWNTHLRKKVD---------KCCKNKKEMKAKAEKVEKINIIKPQPR

S.oleosum. GRLPGRTANDVKNYWNTRLAEKFV-----------YHKPKEDSRPGKCATKVNVIRPRPW

A.formosa.MYB17 GRLPGRTANDIKNYWNSHLSKKME--------------FCHKEYQPSIMPKTEVIRPHPR

Z.jujuba.MYB113-like GRLPGRTANDVKNYWNCHLSKKLN-------------AQETEQNNKNDGNMVNVTRPQPR

A.hypogaea.MYB113 GRLPGRTANDVKNYWNCHLSKKLN--------------AMEGEDRSQATKNVEVIRPQAR

E.guineensis.MYB113-like GRLPGRTANDVKNYWNSHLSKKHD----------------VRDHKERSKHDDKVLKPRPQ

C.melo.MYB90-like GRLPGRTANDIKNYWNCHLSKKLN--------------GQVVEKPNSIKQGSIFGKPSKW

C.maxima.MYB1-like GRLPGRTANDIKNYWNCHLSKKVN--------------GHQGGDQKPNSTK--------W

E.guineensis.MYB1-like GRLPGRTANDIKNYWNTHLGKKVE----------------VENKKAQPRADARVIKPRPW

E.guineensis.MYB1 GRLPGRTANDIKNYWNTHLGKKVE----------------VENKKAEPSADAKVIKPRPW

P.dactylifera.C.Khenezi** GRLPGRTANDIKNYWNSQLSKKVE----------------VENKEAEPSADAKVIKPRPW

P.dactylifera.C.Anbarah GRLPGRTANDIKNYWNSQLSKKVE----------------VENKEAEPSADAKVIKPRPW

P.dactylifera.C.Safawi GRLPGRTANDIKNYWNSQLSKKVE----------------VENKEAEPSADAKVIKPRPW

P.dactylifera.C.Jebeli GRLPGRTANDIKNYWNSQLSKKVE----------------VENKEAEPSADAKVIKPRPW

P.dactylifera.C.Hilwah GRLPGRTANDIKNYWNSQLSKKVE----------------VENKEAEPSADAKVIKPRPW

P.dactylifera.C.Shalaby GRLPGRTANDIKNYWNSQLSKKVE----------------VENKEAEPSADAKVIKPRPW

P.dactylifera.C.Ajwah GRLPGRTANDIKNYWNSQLSKKVE----------------VENKEAEPSADAKVIKPRPW

P.dactylifera.C.Khalas*** GRLPGRTANDIKNYWNSQLSKKVE----------------VENKKAEPSADAKVIKPQPW

P.dactylifera.C.Lulu** GRLPGRTANDIKNYWNSQLSKKVE----------------VENKKAEPSADAKVIKPRPW

P.dactylifera.BC4.Male* GRLPGRTANDIKNYWNSQLSKKVE----------------VENKKAEPSADAKVIKPQPW

P.dactylifera.C.Rothanah GRLPGRTANDIKNYWNSQLSKKVE----------------VENKKAEPSADAKVIKPRPW

P.dactylifera.C.Rabiah GRLPGRTANDIKNYWNSQLSKKVE----------------VENKKAEPSADAKVIKPRPW

P.dactylifera.C.Baydh GRLPGRTANDIKNYWNSQLSKKVE----------------VENKKAEPSADAKVIKPRPW

P.dactylifera.C.Khalas GRLPGRTANDIKNYWNSQLSKKVE----------------VENKKAEPSADAKVIKPRPW

P.dactylifera.Rabiah.Male GRLPGRTANDIKNYWNSQLSKKVE----------------VENKKAEPSADAKVIKPRPW

P.dactylifera.C.Labana GRLPGRTANDIKNYWNSQLSKKVE----------------VENKKAEPSADAKVIKPRPW

P.dactylifera.C.Sukkary GRLPGRTANDIKNYWNSQLSKKVE----------------VENKKAEPSADAKVIKPRPW

*:****:* :**.** .. . .

A.cepa.MYB1 LKETTEDEDD-------TYRKG--------KIEAIYKPQ--------------PKR----

S.tuberosum.MYB113-like NFSNVKKNNS-------HWCNNKSMITNTLDKDDKRCKEIVVNISEKPTGENTSSI----

S.tuberosum.MYBA1 NFSNVKKNNS-------HWCNNKSMITNTLDKDDKRCKEIVVNISEKPTGENTSSI----

S.melongena.MYB1 KYLSSIKKNN-------LTNNN----VIVDKEERCKEIT-------SDKQTTDASM----

T.cacao.MYB75 TL----SKKS-------FPVQLGG--TNINDNNSEAATASHNLLADADNSYYIPAD----

P.pyrifolia.MYB10 KF----IKSS-------YYLSS--------KEPILEHIQ-------SAEDLSTPSQTSSP

P.bretschneideri.MYB75 KF----IKSS-------YYLSS--------KEPILEHIQ-------SAEDLSTPSQTSSS

M.pumila.MYB10 KF----NRSS-------YYLSS--------KEPILDHIQ-------SAEDLSTPPQTSSS

M.hybrid.MYB10 KF----NRSS-------YYLSS--------KEPILDHIQ-------SAEDLSTPPQTSSS

M.domestica.MYB113-like KF----NRSS-------YYLSS--------KEPILDHIQ-------SAEDLSTPPQTSSS

P.mume.PAP1-like SF----IKSS-------NCFSS--------KEPILDHIQ-------TVENFSTPSQTSPS

P.persica.MYB10 SF----IKSS-------NCLSS--------KEPILDHIQ-------TVENFSTPSQTSPS

P.cerasifera.MYB10.1 SF----IKSS-------NCLSS--------KEPILDHIQ-------TVENFSTPSQSSPS

P.salicina.MYB10 RF----TKSS-------NCLSF--------KEPILDHTQ-------LEENFSTTSQTSTS

P.avium.MYB75-like SF----TKSS-------NCLSFKE--PMLDHTQLEENF--------STPSQTSPST----

M.notabilis.MYB113-like TFKKQPSLTNKNNKDTKYWWESLL------DDITFDDGVANSVSSTGLLDHGQPNN----

M.rubra.MYB1 TF----AKNL-------TWFGGK-------PTIMAASFQPKDNVISDLPPAPLP------

O.europaea.MYB113-like TF----KRIA-------PFSSREN--ITFEPNISVTDQNLDT----PSPSSSKPVD----

R.rubrum.MYB10 TF----SKNN-------LSLLMRT--PNSHSQVEITNNNKNKNNINGNVPTSSPLLLSMP

R.nigrum.MYB10 TF----SKNN-------LSLLMRT--PNSHSQVEITNNNKNKNNINGNVPTSSPSSLSMP

A.chinensis.MYB PI----PKTQ-------KTIVPKG------TEAQPRAHP-------KSPPRPSPPS----

C.sinensis.MYB114-like TF----SKNL-------IWLKS--------KTTAIANIETRNNLFKQLSPPLSPPR----

V.corymbosum.MYB1 TF----PKNR-------RVLMG--------STVIADNIQTRDPNLSNPSQTQPPRD----

P.trichocarpa.MYB90 NF-----KNL-------CWLRAGK--GT--PFINVGSQYGDDLCK-PYSTIAFPPS---D

V.vinifera. TF----NKNL-------SYLKGKA--AKESDVQSADHT--------LSKPYSTPPL----

C.medica.Ruby TF----AKNS-------QWLKGKGMTSNNLQLGDYNLGKQSTPSDHHHHHQQQQEN----

S.oleosum. TL----SKSS-------AWFMG--------KATLVASWTP------PGENPGQPSPVLSR

A.formosa.MYB17 TF----NANL-------VRSRGKFILKNQQPQQQKQKQK-QQQLEQEQYDAVVPTE----

Z.jujuba.MYB113-like NI----EKSC--------------------SRRPCFKPPEDA----GSTSTPVPCI----

A.hypogaea.MYB113 NI----GTSS-------SMKRRR-------GIQGESSSPIESDLLAQQEISGMSSTMSFD

E.guineensis.MYB113-like TI----PRTW-------IWSRD--------HLSSASEIQQE-----ESEMQEIPSS----

C.melo.MYB90-like K-----PLQE-------ESSKSKG--KEYVDDDDQNNNESQGILVENNQNQNTPIV----

C.maxima.MYB1-like K-----PIQE-------ESSKSKE--KEYIEDQNGEKQG----ILIQNQNQNVPIV----

E.guineensis.MYB1-like RV----PLQW-------IWSR---------DQQSFGSQH-------QQKELGIPEL---P

E.guineensis.MYB1 KV----PLQW-------FWSE---------DQQSCGSQQ-------PQEEFGIPEL---P

P.dactylifera.C.Khenezi** RV----PLQW-------IW-SG--------DQQSCGSQH-------QQEEFGIPEL---P

P.dactylifera.C.Anbarah RV----PLQW-------IW-SG--------DQQSCGSQH-------QQEEFGIPEL---P

P.dactylifera.C.Safawi RV----PLQW-------IW-SG--------DQQSCGSQH-------QQEEFGIPEL---P

P.dactylifera.C.Jebeli RV----PLQW-------IW-SG--------DQQSCGSQH-------QQEEFGIPEL---P

P.dactylifera.C.Hilwah RV----PLQW-------IW-SG--------DQQSCGSQH-------QQEEFGIPEL---P

P.dactylifera.C.Shalaby RV----PLQW-------IW-SG--------DQQSCGSQH-------QQEEFGIPEL---P

P.dactylifera.C.Ajwah RV----PLQW-------IW-SG--------DQQSCGSQH-------QQEEFGIPEL---P

P.dactylifera.C.Khalas*** RV----PLQW-------IW-SG--------DQQSCGSQH-------QQEKFGIPVL---E

P.dactylifera.C.Lulu** RV----PLQW-------IW-SG--------DQQSCGSQH-------QQEKFGIPVL---E

P.dactylifera.BC4.Male* RV----PLQW-------IW-SG--------DQQSCGSQH-------QQEKFGIPVL---E

P.dactylifera.C.Rothanah RV----PLQW-------IW-SG--------DQQSCGSQH-------QQEKFGIPVL---E

P.dactylifera.C.Rabiah RV----PLQW-------IW-SG--------DQQSCGSQH-------QQEKFGIPVL---E

P.dactylifera.C.Baydh RV----PLQW-------IW-SG--------DQQSCGSQH-------QQEKFGIPVL---E

P.dactylifera.C.Khalas RV----PLQW-------IW-SG--------DQQSCGSQH-------QQEKFGIPVL---E

P.dactylifera.Rabiah.Male RV----PLQW-------IW-SG--------DQQSCGSQH-------QQEKFGIPVL---E

P.dactylifera.C.Labana RV----PLQW-------IW-SG--------DQQSCGSQH-------QQEKFGIPVL---E

P.dactylifera.C.Sukkary RV----PLQW-------IW-SG--------DQQSCGSQH-------QQEKFGIPVL---E

A.cepa.MYB1 ----------------HSSSEAW-----NKAVMVQPEESTAMANETNE-----CLLTDLM

S.tuberosum.MYB113-like ----------------DDGVQWW------TNLLENCNEIEEEVAVTNF-----EKTPTML

S.tuberosum.MYBA1 ----------------DDGVQWW------TNLLENCNEIEEEVAVTNF-----EKTPTML

S.melongena.MYB1 ----------------DNGDQWWKSLLENFNDDAVEGEEEAVTNYEKT-------LTSLL

T.cacao.MYB75 ----------------NDQIIWW------ENLLINDNDAVDVQHAAAS--FNNSNTKSGT

P.pyrifolia.MYB10 T---------------KNGNDWW------ETLLEGEDTFERAPCPSIE--LEEELFTTFW

P.bretschneideri.MYB75 T---------------KNGNDWW------ETLFEGEDTFERAACPSIE--LEEELFTTFW

M.pumila.MYB10 T---------------KNGNDWW------ETLLEGEDTFERAAYPSIE--LEEELFTSFW

M.hybrid.MYB10 T---------------KNGNDWW------ETLLEGEDTFERAAYPSIE--LEEELFTSFW

M.domestica.MYB113-like T---------------KNGNDWW------ETLLEGEDTFERAAYPSIE--LEEELFTSFW

P.mume.PAP1-like T---------------KNGNDWW------ETFLDDEDAFERATCYGLA--LEEEEFTSFW

P.persica.MYB10 T---------------KNGNDWW------ETFLDDEDVFERATCYGLA--LEEEEFTSFW

P.cerasifera.MYB10.1 T---------------KNGNDWW------ETFLDDEDVFEKATCYGLA--LEEEEFTSFW

P.salicina.MYB10 T---------------RIGSDWW------ETFLDDKDATETATGSGLG--LDEELLASFW

P.avium.MYB75-like ----------------RIGNDWW------DTFLDDNDALETATSSGPE--LDEELLTSFW

M.notabilis.MYB113-like ----------------GNIANFL------SELEADQPALSISPRPTDITDSATSTCVNL-

M.rubra.MYB1 ----------------SENSVKW-----GENLFDDKEAGDEIGTYDVGGLNEEPIATFRW

O.europaea.MYB113-like ----------------DECSQWW---------------------------------SNLL

R.rubrum.MYB10 RDSSSSPLSLSKPLDSRDDIDWW----------------ENLISWSED-----EPISNLW

R.nigrum.MYB10 RDSSSSPLSLSKPLDSRDDIDWW----------------ENLISWSED-----EPISNLW

A.chinensis.MYB ----------------NNEILWW-------------DNKTVSPQIDNI-GIHWSIDGSIF

C.sinensis.MYB114-like ----------------DDGISWW-----ENMFVDLEINKEITLSIDGS-------NEEKW

V.corymbosum.MYB1 ----------------DDGTLWW------DDMLFDYEISRGMMTWTNDGSNEEAMMVDNG

P.trichocarpa.MYB90 TD--------------EVERMWWESLLDDKEINLTNRNSCQNSCLGSGSTANQEPINSLF

V.vinifera. ----------------SEDGISW-----LDSLFSGKEGHKESTCFRNG--SGKESISSFW

C.medica.Ruby ----------------ETESVWW------ESFLFGDELDQQGISSLLSRPEEESTTANIF

S.oleosum. Q---------------DKESTWW------ENLFTNSGPDEGLLTSRST--LEGQPDANSW

A.formosa.MYB17 ----------------AELSTWW-----------NDLANDLNISVPNA--------KEVI

Z.jujuba.MYB113-like ----------------EQSQIYL--------------EDNGYHTWNEEYGAVGGFSTNIQ

A.hypogaea.MYB113 AD--------------GQSH---------HNMLESQLENNIYSCLEHQ-DIVGELPMDFR

E.guineensis.MYB113-like ----------------SKNHKTW-----------VDAQIIGVPNVENV------ITKEMQ

C.melo.MYB90-like ------------------------------------IEQNSSMSLGNM------QMDLFQ

C.maxima.MYB1-like -------------------------------------EQSGSIRMENI-------QMDYQ

E.guineensis.MYB1-like TT--------------LGNDEAW-----LNSIINGDGENSAMPDIGNV------NTMNLQ

E.guineensis.MYB1 TI--------------SENDEAW-----LNSIMNGDRENSAVPDVGNG------STMNLQ

P.dactylifera.C.Khenezi** TI--------------WENDEAW-----LNSIINGDGESDAMPDVRNL------NTTNLQ

P.dactylifera.C.Anbarah TI--------------WENDEAW-----LNSIINGDGKSDAMPDVRNL------NTTNLQ

P.dactylifera.C.Safawi TI--------------WENDEAW-----LNSIINGDGKSDAMPDVRNL------NTTNLQ

P.dactylifera.C.Jebeli TI--------------WENDEAW-----LNSIINGDGESDAMPDVRNL------NTTNLQ

P.dactylifera.C.Hilwah TI--------------WENDEAW-----LNSIINGDGESDAMPDVRNL------NTTNLQ

P.dactylifera.C.Shalaby TI--------------WENDEAW-----LNSIINGDGESDAMPDVRNL------NTTNLQ

P.dactylifera.C.Ajwah TI--------------WENDEAW-----LNSIINGDGKSDAMPDVRNL------NTTNLQ

P.dactylifera.C.Khalas*** ------------------------------------------------------------

P.dactylifera.C.Lulu** ------------------------------------------------------------

P.dactylifera.BC4.Male* ------------------------------------------------------------

P.dactylifera.C.Rothanah ------------------------------------------------------------

P.dactylifera.C.Rabiah ------------------------------------------------------------

P.dactylifera.C.Baydh ------------------------------------------------------------

P.dactylifera.C.Khalas ------------------------------------------------------------

P.dactylifera.Rabiah.Male ------------------------------------------------------------

P.dactylifera.C.Labana ------------------------------------------------------------

P.dactylifera.C.Sukkary ------------------------------------------------------------

A.cepa.MYB1 KENSDNPVSLDYEFDMTDQALLLDAFN------DWEELLNS-------------------

S.tuberosum.MYB113-like --LHEEISPPLINGE---GNSMQQGQSH-----DWDDFSTD-----IDLWNLLN------

S.tuberosum.MYBA1 --LHEEISPPLINGE---GNSMQQGQSH-----DWDDFSTD-----IDLWNLLN------

S.melongena.MYB1 --HEEISSPPLNGGG---NSMQQEQCD------NWDDFFAD-----IDLWNLLD------

T.cacao.MYB75 --GDQTVLNSIDKYE---EIGERTMVVEVGNNSSWDELFRD-----VQLWNVFNPEPEDY

P.pyrifolia.MYB10 --FDDRLSARS-------CANFPEEGQ------SRSEFSFS-----MDLWNHSKEE----

P.bretschneideri.MYB75 --FDDRLSARS-------CANFPEEGQ------SRSEFSFS-----MDLWNHSKEE----

M.pumila.MYB10 --FDDRLSPRS-------CANFPEGR-------SRSEFSFS-----TDLWNHSKEE----

M.hybrid.MYB10 --FDDRLSPRS-------CANFPEGX-------SRSEFXFS-----TDLWNHSKEE----

M.domestica.MYB113-like --FDDRLSPRS-------CANFPEGQ-------SRSEFSFS-----TDLWNHSKEE----

P.mume.PAP1-like --VDDMPQSKRQ------CTNASERGL------GGGDFSFN-----VDLWNHSKQ-----

P.persica.MYB10 --VDDMPQSKRQ------CTNVSEEGL------GRGDFSFN-----VDFWNH--------

P.cerasifera.MYB10.1 --VDDMPQSKRQ------CTNVTEEGL------GTGDFSFN-----VDFWNH--------

P.salicina.MYB10 --VDDDMPQSTRT-----CVNFSEEGL------SRGDFSFS-----VDLWNHSNEE----

P.avium.MYB75-like --DDDDMPQSTRTCI---NFSEEEL--------SRGDFSFN-----LDLWNHSKEE----

M.notabilis.MYB113-like --VEDDYGHQN----------------------SWTDFIMD-----VELWGLPSTDQGNA

M.rubra.MYB1 --AEAAPAETV-------GTPLDEFGPS-----FWAEFPSN-----LDVWDFLDP-----

O.europaea.MYB113-like --DSVKIDGEPEPGS---NAISLDKD-------IWELFEF--------------------

R.rubrum.MYB10 --AEEEVGGRGNEST---LVQEDQSGLLNN---NNESPSFD-----MDIWNFLNVEKEMV

R.nigrum.MYB10 --AEEEVGGRGNEST---LVQEDQSGLLNN---NNESPSFD-----MDIWNFLNVEKEMV

A.chinensis.MYB --EEPILGNLQSPGD---SFLQQNQS-------DWSDIFLED----VNLWDLLGDD----

C.sinensis.MYB114-like --HEKETQGILAIGD---SSVQGES--------DWNDIFIDK----MDLWDL--------

V.corymbosum.MYB1 --EEAKSGTQGVGGDHYSCVQEDQS--------DWSNIFMDN----VDLWDIL-------

P.trichocarpa.MYB90 --VEANPPGGIMIGD---VFSDQGQN-------RWGDISFD-----ADLWSLIDTEIDQQ

V.vinifera. ---DEEISAATKVGE---TFVEENES-------DWNHFSFD-----MDLWDLLNA-----

C.medica.Ruby --AEKSPVVTKVKEN---RVIEAGQSC------TTDDFAFD-----EELCDLLNAN----

S.oleosum. --PRESMEERQN---------------------GWNELSFD-----ADVWELLGNSPQAV

A.formosa.MYB17 --PTNSTVENREAMNPFEKTIFDDI--------AWDDFLATF------------------

Z.jujuba.MYB113-like --FEQVREEDETNGKW-----------------DLDDFILD-----LDLWNDSF------

A.hypogaea.MYB113 --FEGGVEEIINAGE---GSSGQW---------DWDDLLLD-----MDLYKDFATN----

E.guineensis.MYB113-like --TDNRVIQDDNKDD---ESLTGVG--------GWEDLLQD-----IDIWGDLGTI----

C.melo.MYB90-like --FDQQVLKAMEDED---GCNKRE---------LWDDWISE-----MDLWIDSL------

C.maxima.MYB1-like --FDQEVVAMAD-GD---GCNKRE---------LWDDWVSE-----MDLWIDSL------

E.guineensis.MYB1-like --NGFGIGELEENRD---GALFMEGEACLLT--RKVEVSPYVRVNCREALKRTGIGLLKI

E.guineensis.MYB1 --NEFGIGGLEENRD---GAVFLEGVL------GWDDLLWATI-----------------

P.dactylifera.C.Khenezi** --SGFGIGELEENRD---GALFLEGVL------GWDDLLSHH------------------

P.dactylifera.C.Anbarah --SGFGIGELEENRD---GALFLEGIL------GWDDLLSHH------------------

P.dactylifera.C.Safawi --SGFGIGELEENRD---GALFLEGIL------GWDDLLSHH------------------

P.dactylifera.C.Jebeli --SGFGIGELEENRD---GALFLEGVL------GWDDLLSHH------------------

P.dactylifera.C.Hilwah --SGFGIGELEENRD---GALFLEGVL------GWDDLLSHH------------------

P.dactylifera.C.Shalaby --SGFGIGELEENRD---GALFLEGVL------GWDDLLSHH------------------

P.dactylifera.C.Ajwah --SGFGIGELEENRD---GALFLEGVL------GWDDLLSHH------------------

P.dactylifera.C.Khalas*** ------------------------------------------------------------

P.dactylifera.C.Lulu** ------------------------------------------------------------

P.dactylifera.BC4.Male* ------------------------------------------------------------

P.dactylifera.C.Rothanah ------------------------------------------------------------

P.dactylifera.C.Rabiah ------------------------------------------------------------

P.dactylifera.C.Baydh ------------------------------------------------------------

P.dactylifera.C.Khalas ------------------------------------------------------------

P.dactylifera.Rabiah.Male ------------------------------------------------------------

P.dactylifera.C.Labana ------------------------------------------------------------

P.dactylifera.C.Sukkary ------------------------------------------------------------

A.cepa.MYB1 ----

S.tuberosum.MYB113-like ----

S.tuberosum.MYBA1 ----

S.melongena.MYB1 ----

T.cacao.MYB75 V---

P.pyrifolia.MYB10 ----

P.bretschneideri.MYB75 ----

M.pumila.MYB10 ----

M.hybrid.MYB10 ----

M.domestica.MYB113-like ----

P.mume.PAP1-like ----

P.persica.MYB10 ----

P.cerasifera.MYB10.1 ----

P.salicina.MYB10 ----

P.avium.MYB75-like ----

M.notabilis.MYB113-like EMQ-

M.rubra.MYB1 ----

O.europaea.MYB113-like ----

R.rubrum.MYB10 A---

R.nigrum.MYB10 A---

A.chinensis.MYB ----

C.sinensis.MYB114-like ----

V.corymbosum.MYB1 ----

P.trichocarpa.MYB90 ----

V.vinifera. ----

C.medica.Ruby ----

S.oleosum. I---

A.formosa.MYB17 ----

Z.jujuba.MYB113-like ----

A.hypogaea.MYB113 ----

E.guineensis.MYB113-like ----

C.melo.MYB90-like ----

C.maxima.MYB1-like ----

E.guineensis.MYB1-like KKKA

E.guineensis.MYB1 ----

P.dactylifera.C.Khenezi** ----

P.dactylifera.C.Anbarah ----

P.dactylifera.C.Safawi ----

P.dactylifera.C.Jebeli ----

P.dactylifera.C.Hilwah ----

P.dactylifera.C.Shalaby ----

P.dactylifera.C.Ajwah ----

P.dactylifera.C.Khalas*** ----

P.dactylifera.C.Lulu** ----

P.dactylifera.BC4.Male* ----

P.dactylifera.C.Rothanah ----

P.dactylifera.C.Rabiah ----

P.dactylifera.C.Baydh ----

P.dactylifera.C.Khalas ----

P.dactylifera.Rabiah.Male ----

P.dactylifera.C.Labana ----

P.dactylifera.C.Sukkary ----

**Figure S5.** Multiple protein sequence alignment comparing sequence of different R2R3 MYB genes from other plants species.
